# Supplementary figures and images for: Plausible pathway for a host-parasite molecular replication network to increase its complexity through Darwinian evolution
Source: PLoS Comput Biol. 2022 Dec 1;18(12):e1010709. doi: 10.1371/journal.pcbi.1010709 (PMC9714742; doi:10.1371/journal.pcbi.1010709)

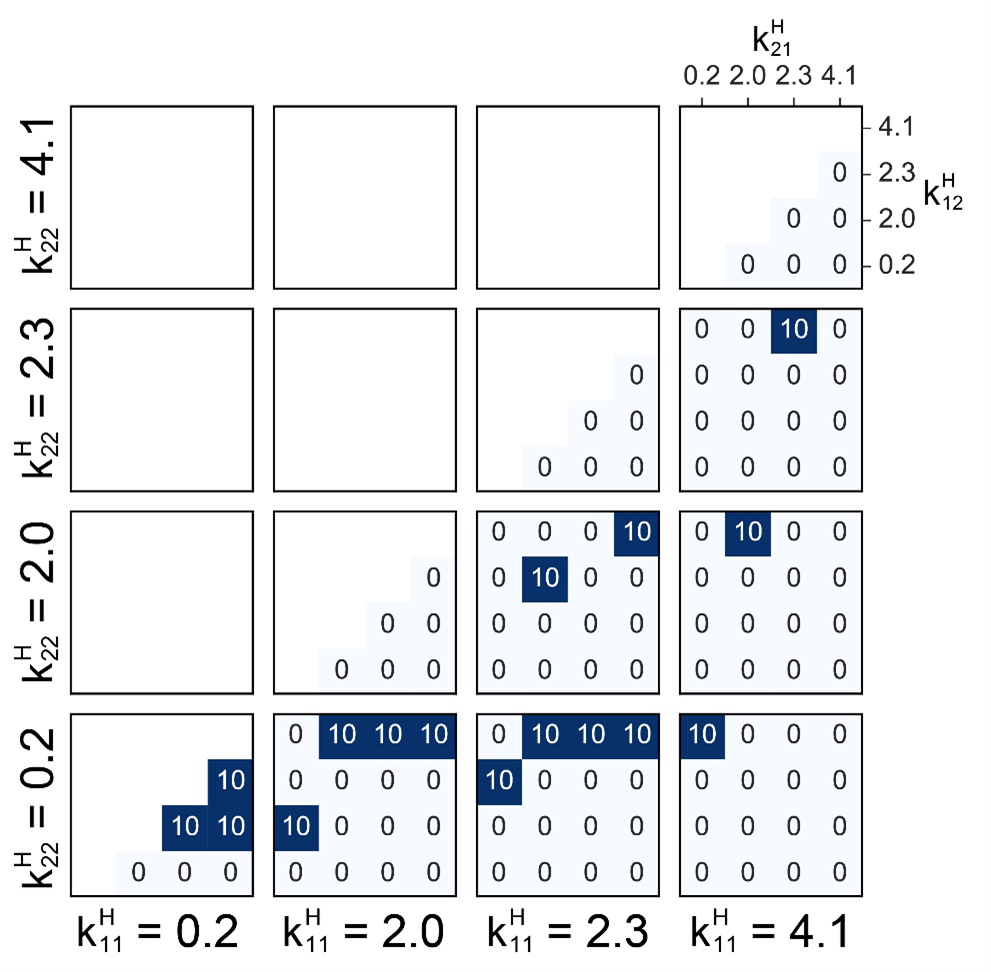

Supplement: S1 Fig — The simulation procedure was the same as that shown in Fig 3B except for using smaller (0.2) and larger (4.1) parameter values and a smaller number [10] of independent simulations. (TIF) [file pcbi.1010709.s001.tif]

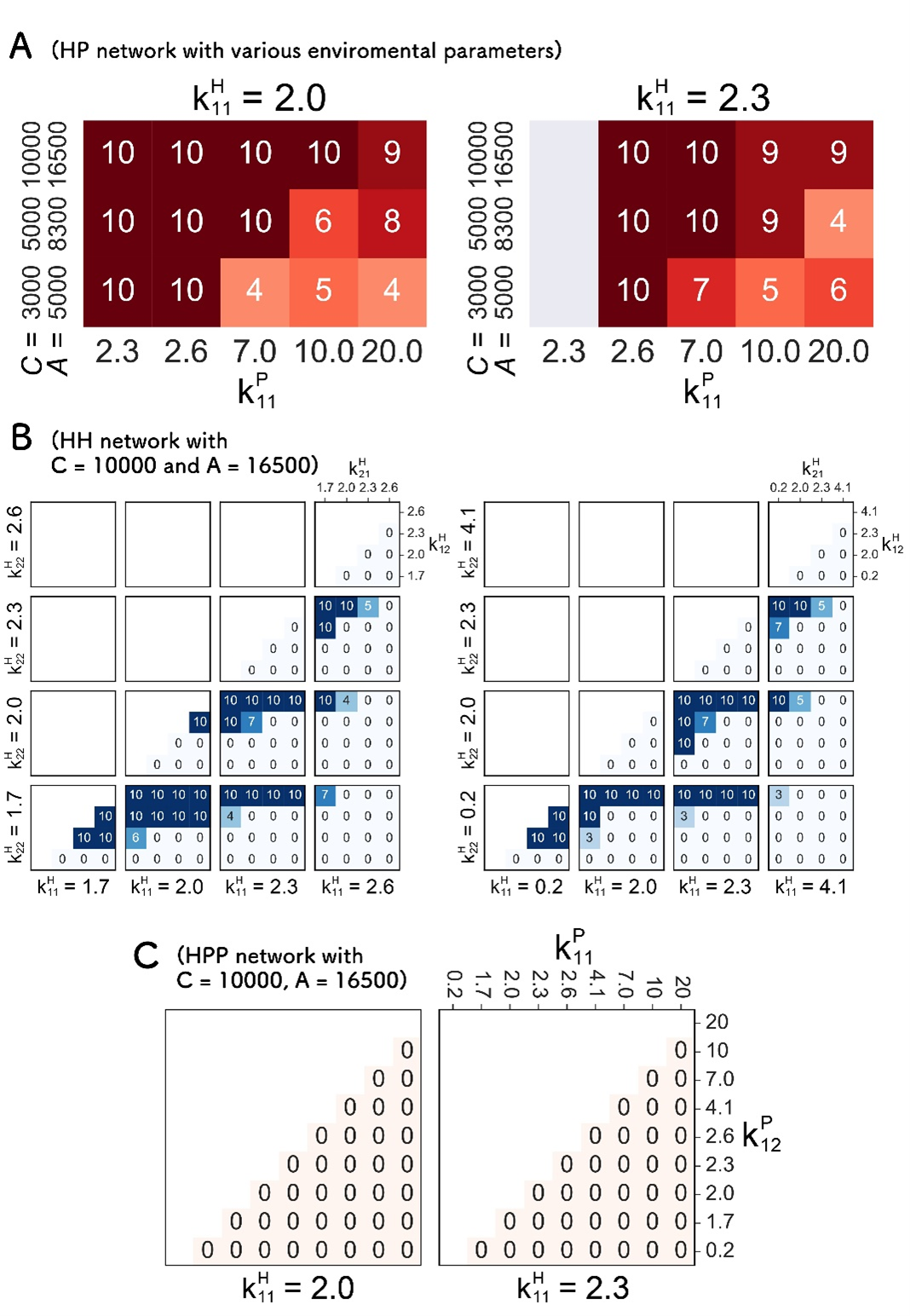

Supplement: S2 Fig — The number of compartments and the frequency of fusion-division were increased to 10,000 and 16,500, respectively. The number of runs in which all three replicators (Hosts 1 and 2, and the parasite) were sustained for 100 rounds out of 10 independent simulations are shown. (A) HP network. The replication coefficient for the host self-replication is fixed at 2.0 or 2.3. (B) HH network. (C) HPP network. (TIF) [file pcbi.1010709.s002.tif]

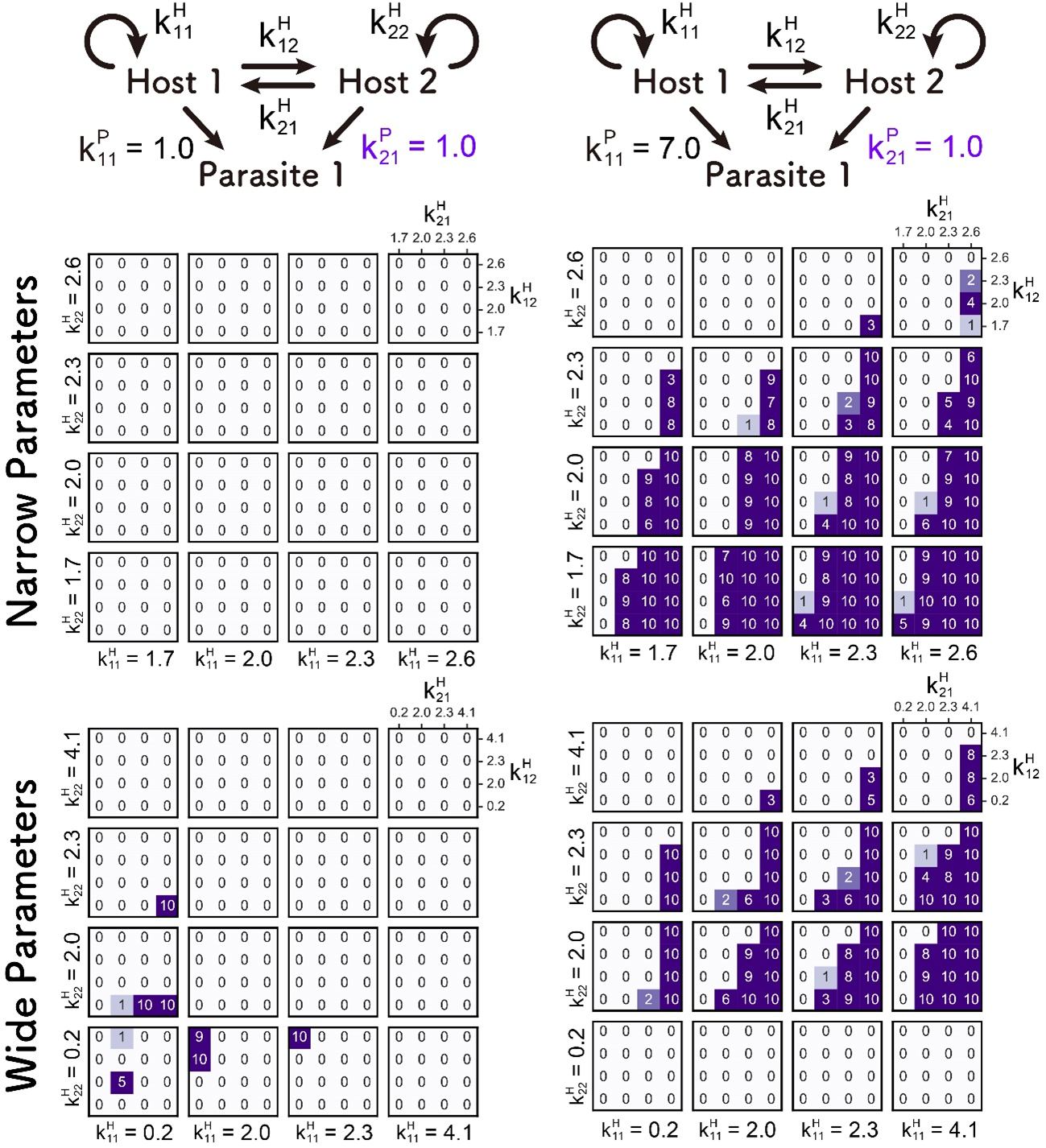

Supplement: S3 Fig — The simulations of the HHP network were conducted by the same method as Fig 5 except for employing an intermediate k21P value (1.0). The number of runs in which all three replicators (Hosts 1 and 2, and Parasite 1) were sustained for 100 rounds in 10 independent simulations are shown. (TIF) [file pcbi.1010709.s003.tif]

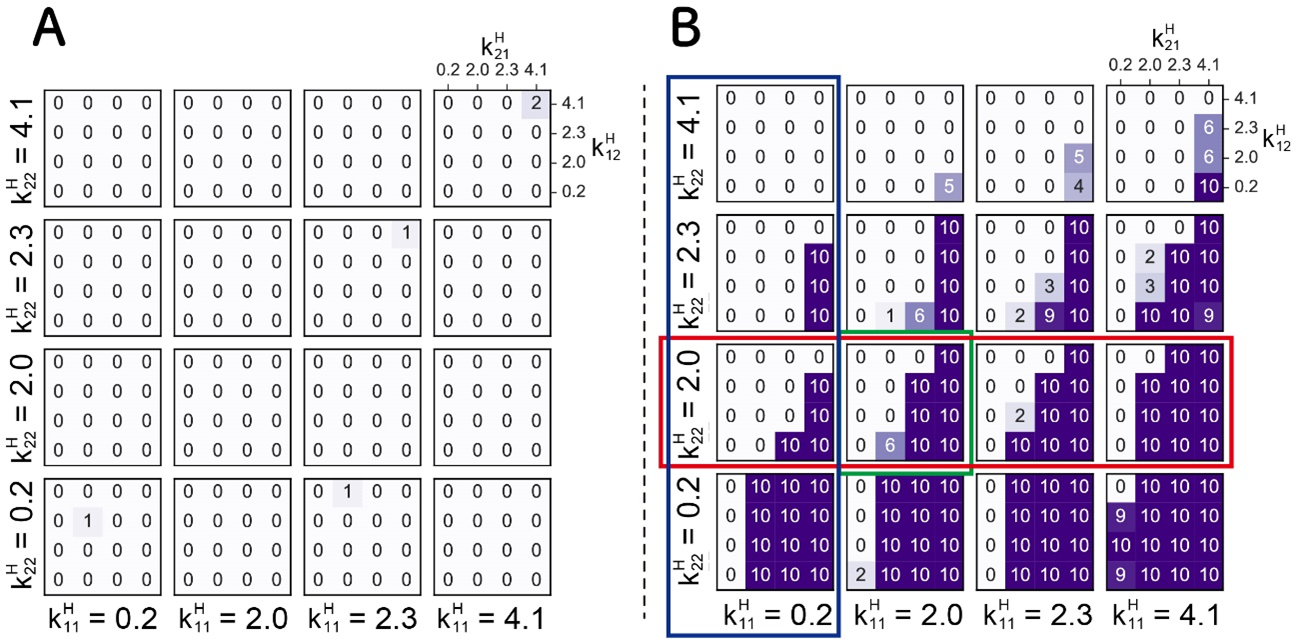

Supplement: S4 Fig — The simulations of the HHP network were conducted in the symmetrical (A) or asymmetrical cases (B) by the same method as Fig 5 except for employing extreme parameter values (0.2 and 4.1). The number of runs in which all three replicators (Hosts 1 and 2, and the parasite) were sustained for 100 rounds in 10 independent simulations are shown. (TIF) [file pcbi.1010709.s004.tif]

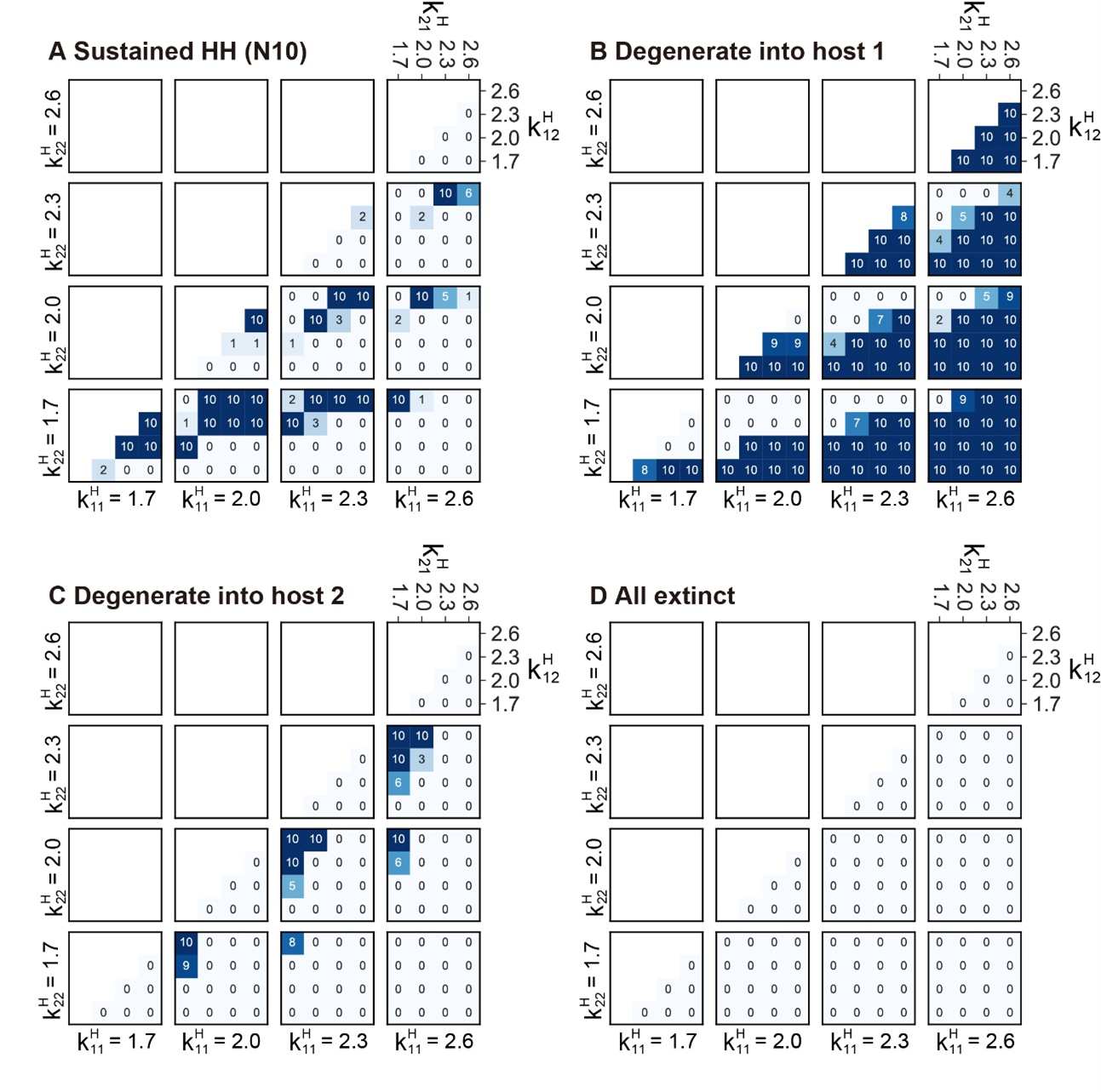

Supplement: S5 Fig — Simulations were conducted as described in Fig 3B for 10 times. (TIF) [file pcbi.1010709.s005.tif]

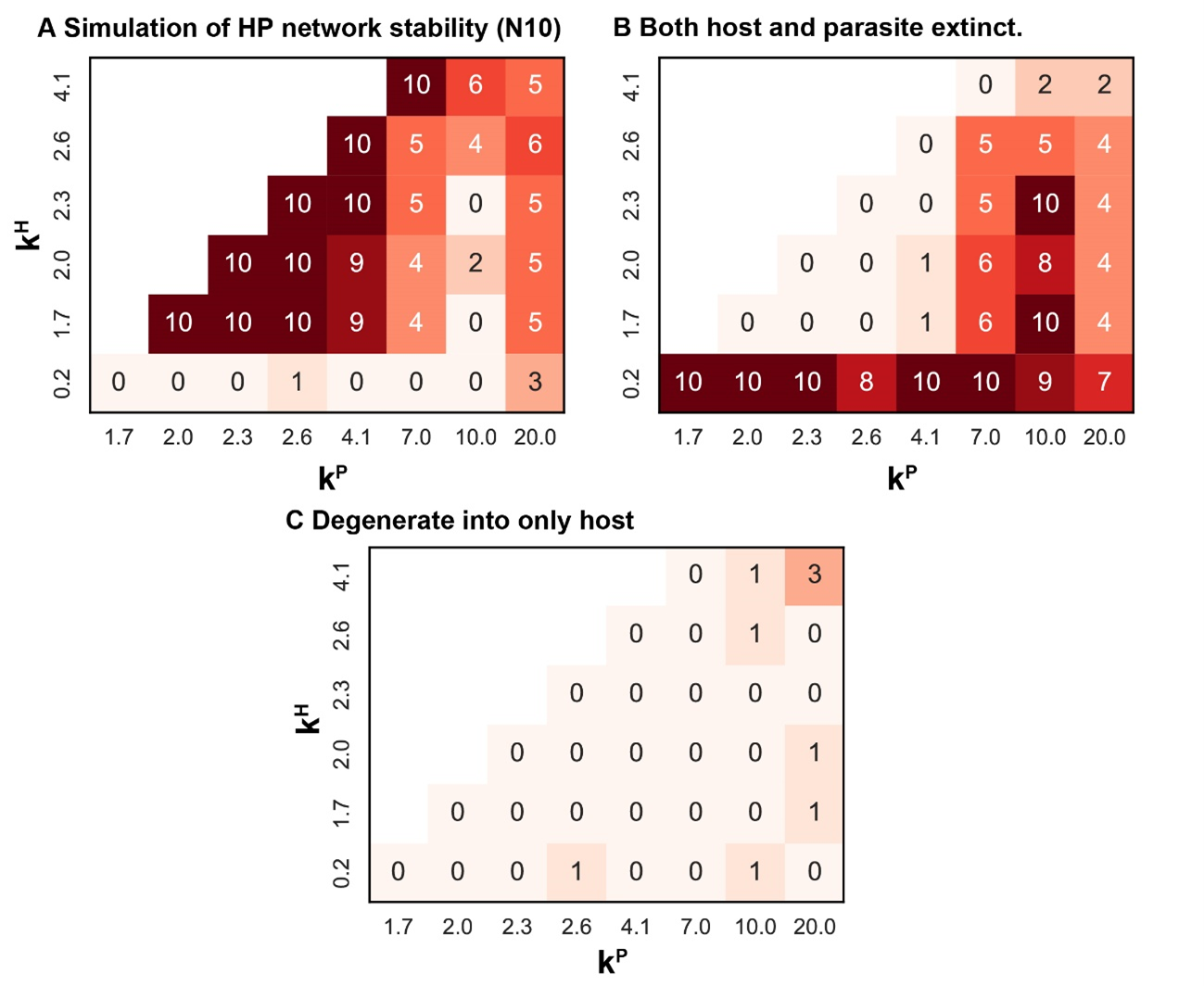

Supplement: S6 Fig — Simulations were conducted as described in Fig 3F for 10 times. (TIF) [file pcbi.1010709.s006.tif]

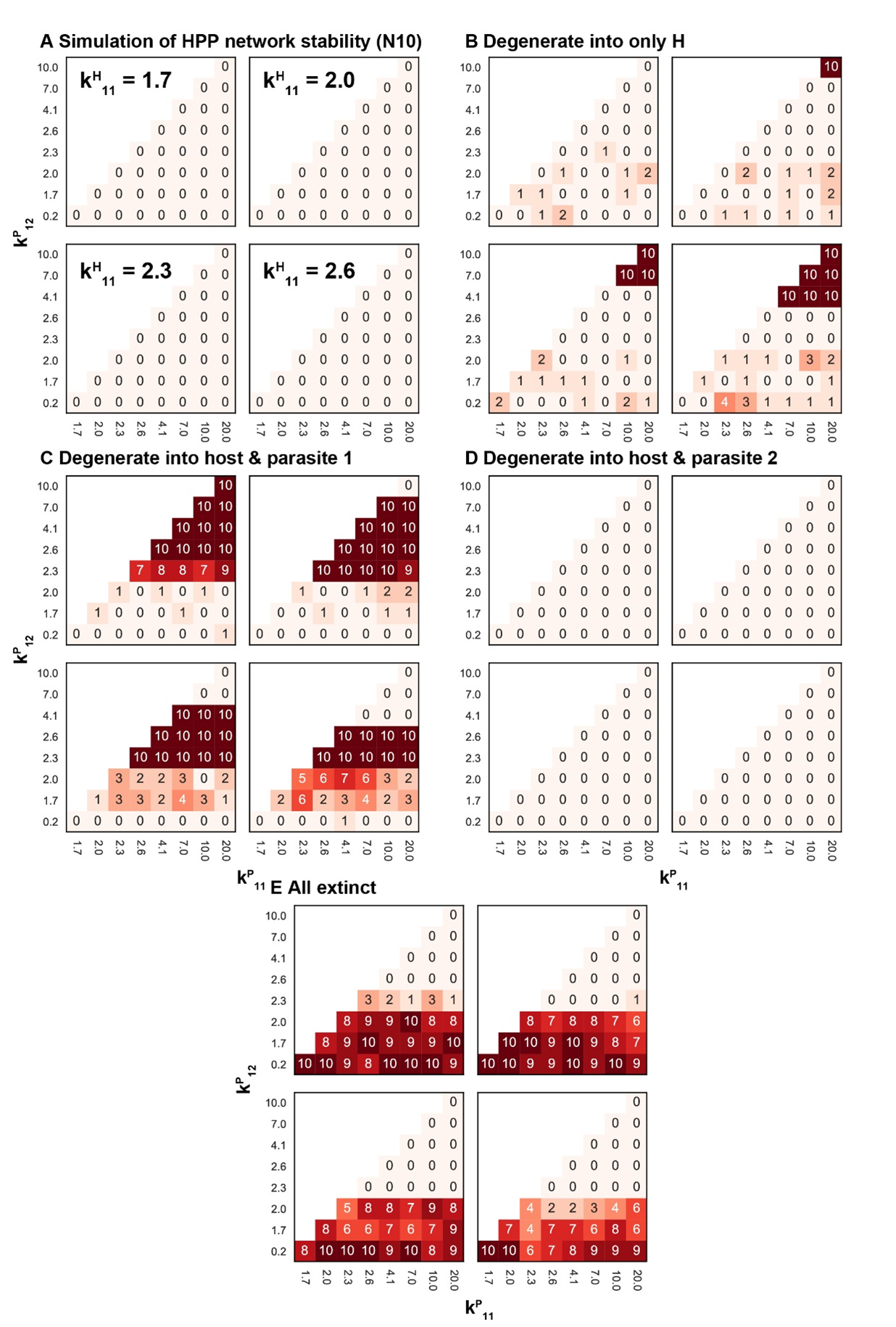

Supplement: S7 Fig — Simulations were conducted as described in Fig 4B for 10 times. (TIF) [file pcbi.1010709.s007.tif]

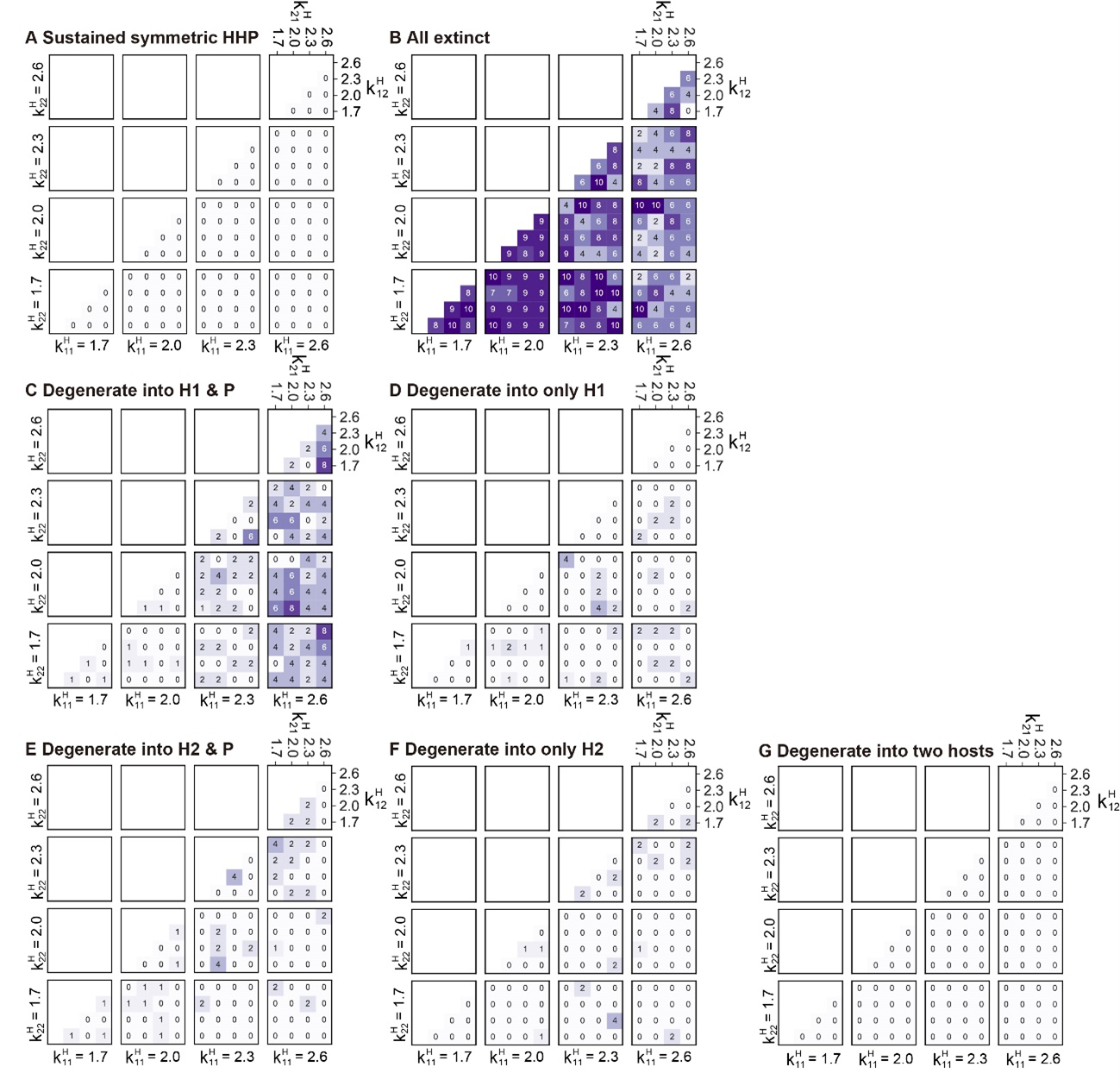

Supplement: S8 Fig — Simulations were conducted as described in Fig 5B for 10 times. (TIF) [file pcbi.1010709.s008.tif]

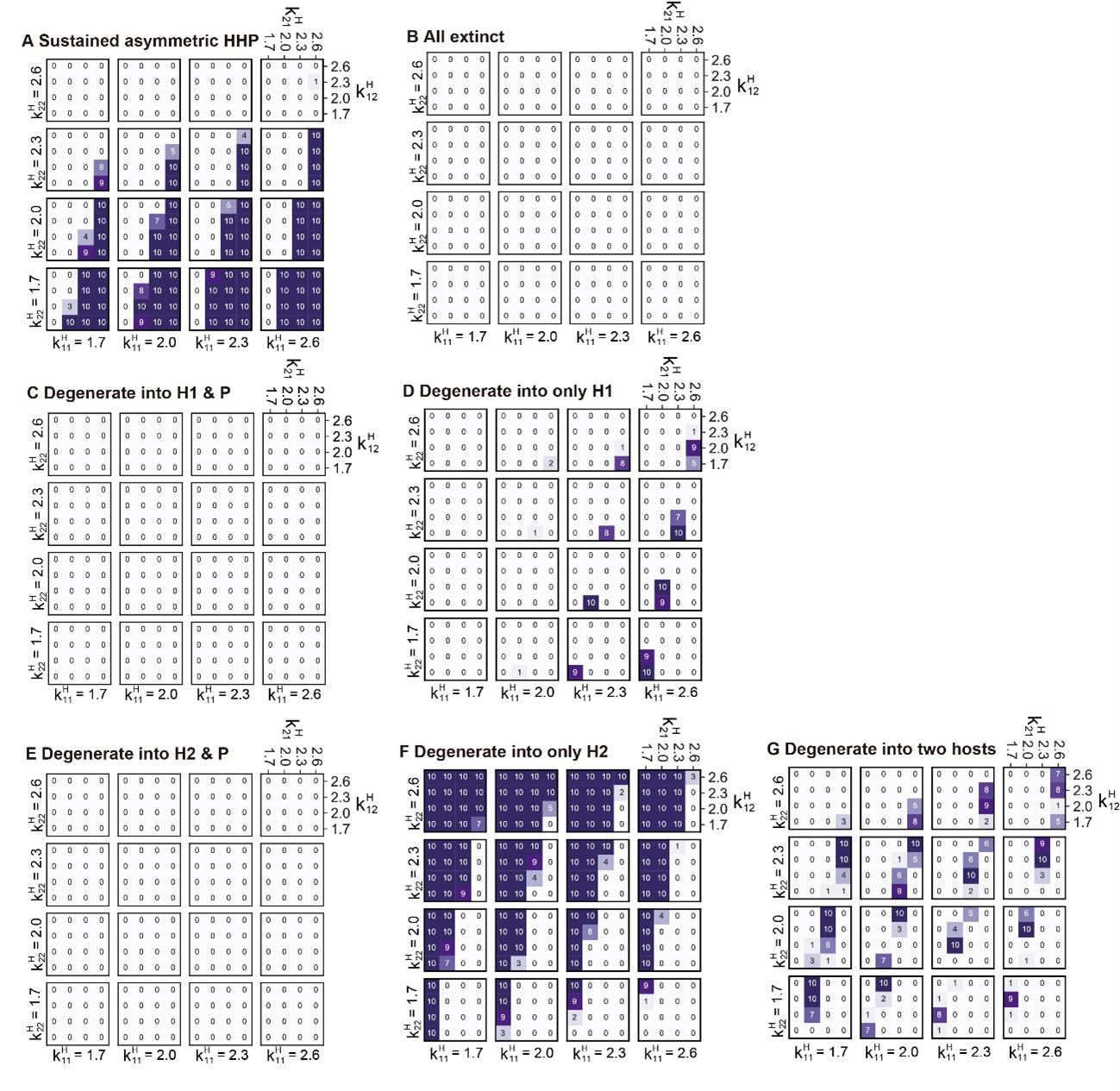

Supplement: S9 Fig — Simulations were conducted as described in Fig 5D for 10 times. (TIF) [file pcbi.1010709.s009.tif]

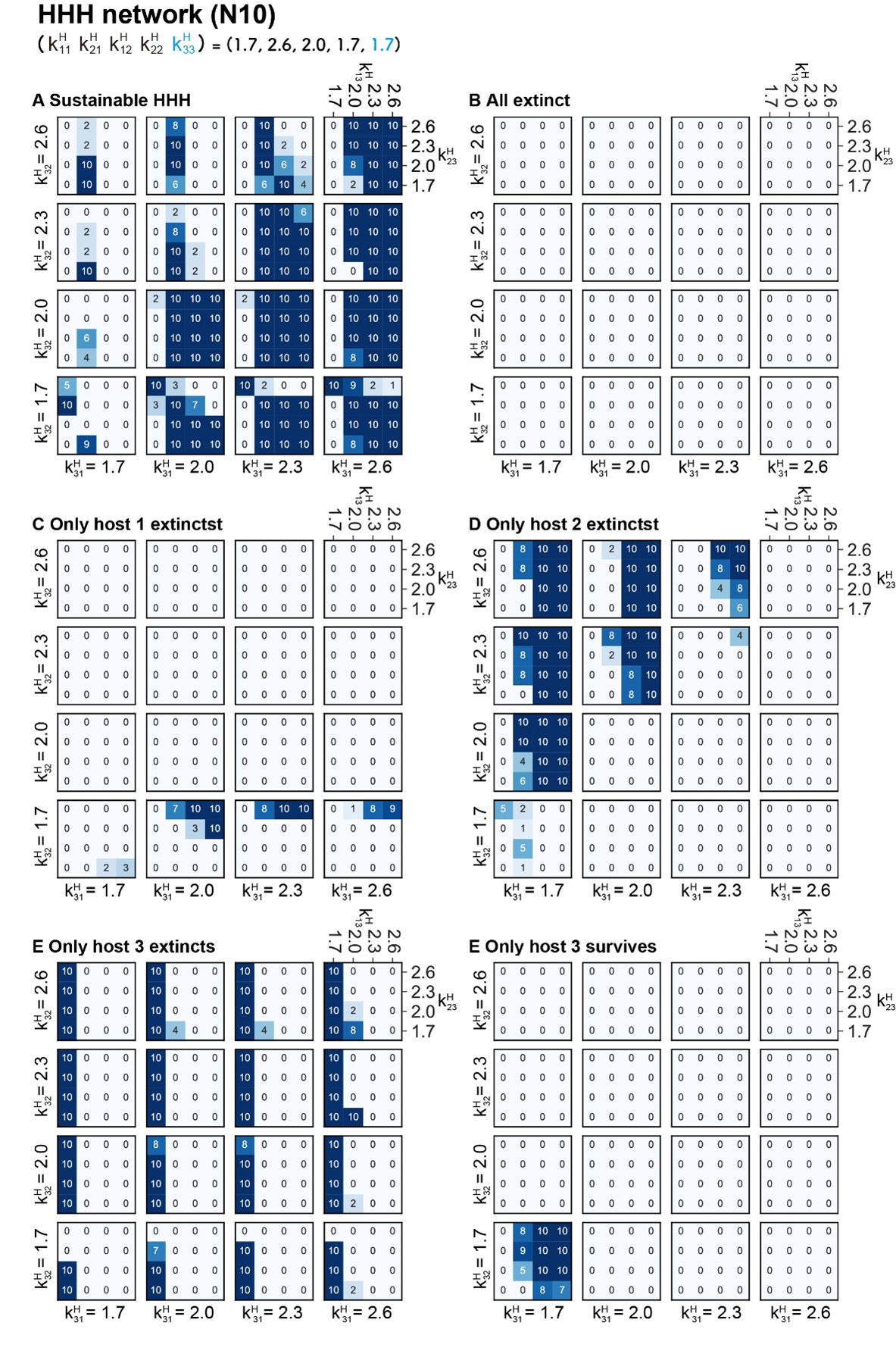

Supplement: S10 Fig — Simulations were conducted as described in Fig 6B for 10 times. (TIF) [file pcbi.1010709.s010.tif]

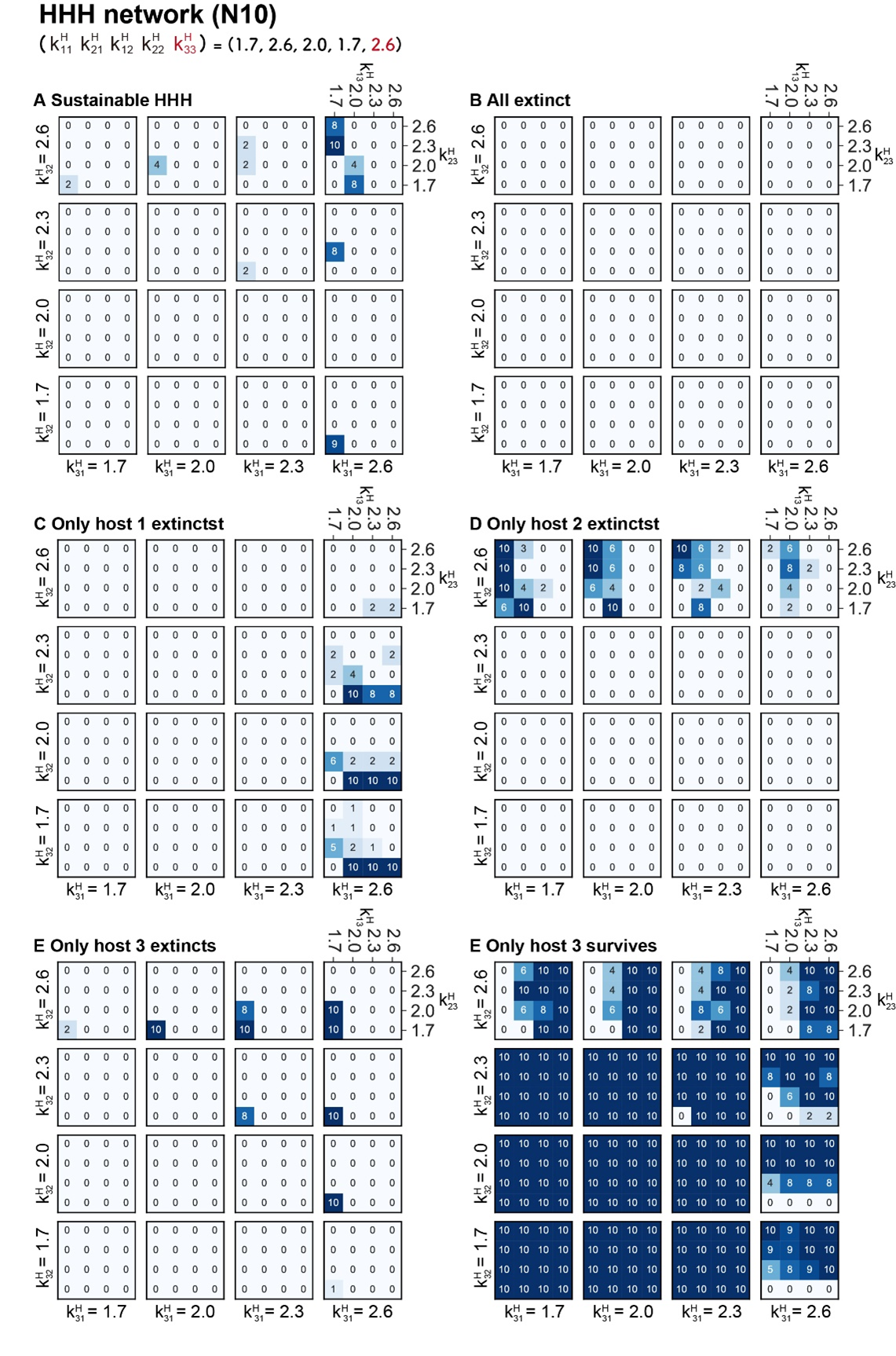

Supplement: S11 Fig — Simulations were conducted as described in Fig 6C for 10 times. (TIF) [file pcbi.1010709.s011.tif]

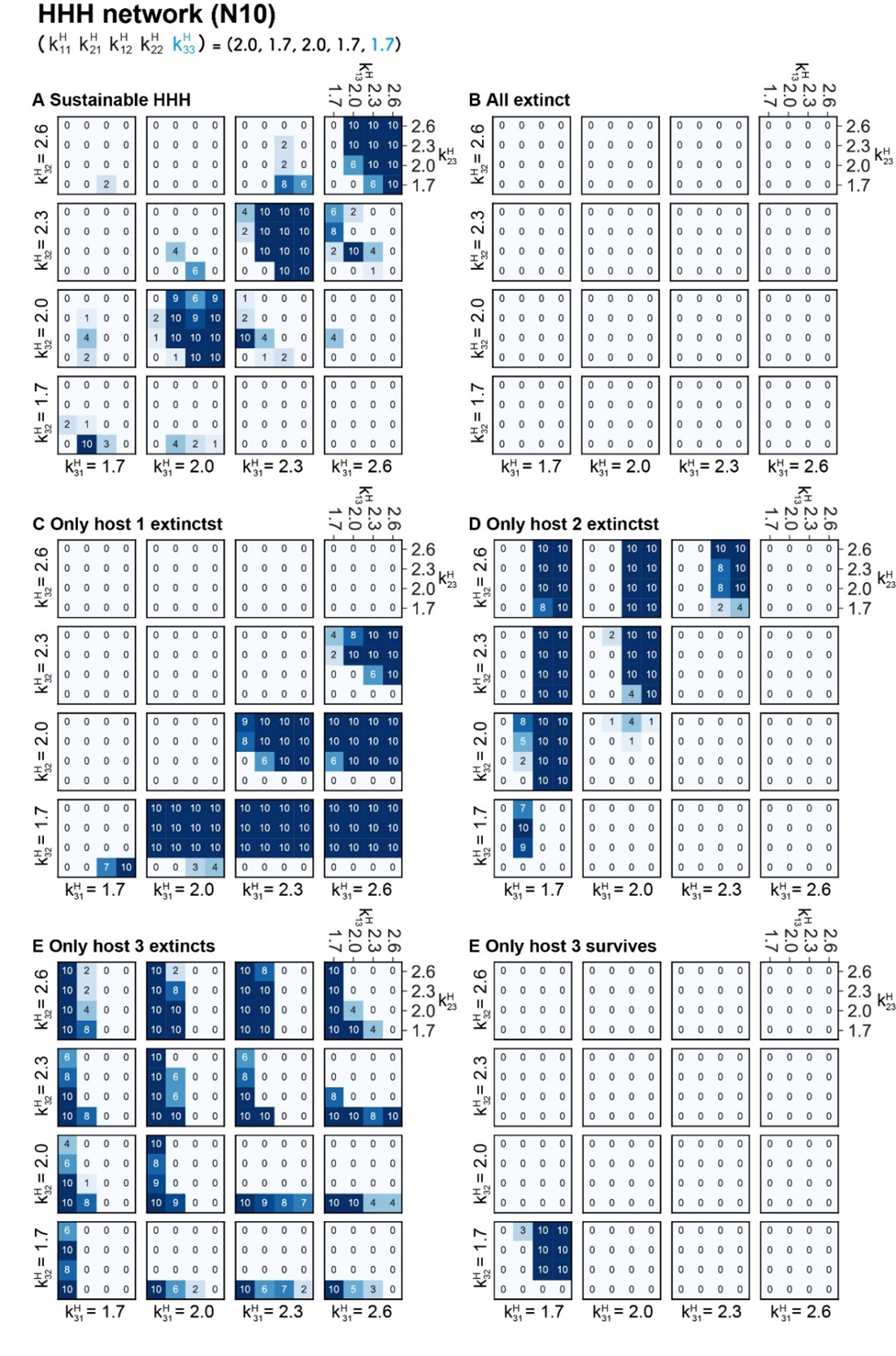

Supplement: S12 Fig — Simulations were conducted as described in Fig 6D for 10 times. (TIF) [file pcbi.1010709.s012.tif]

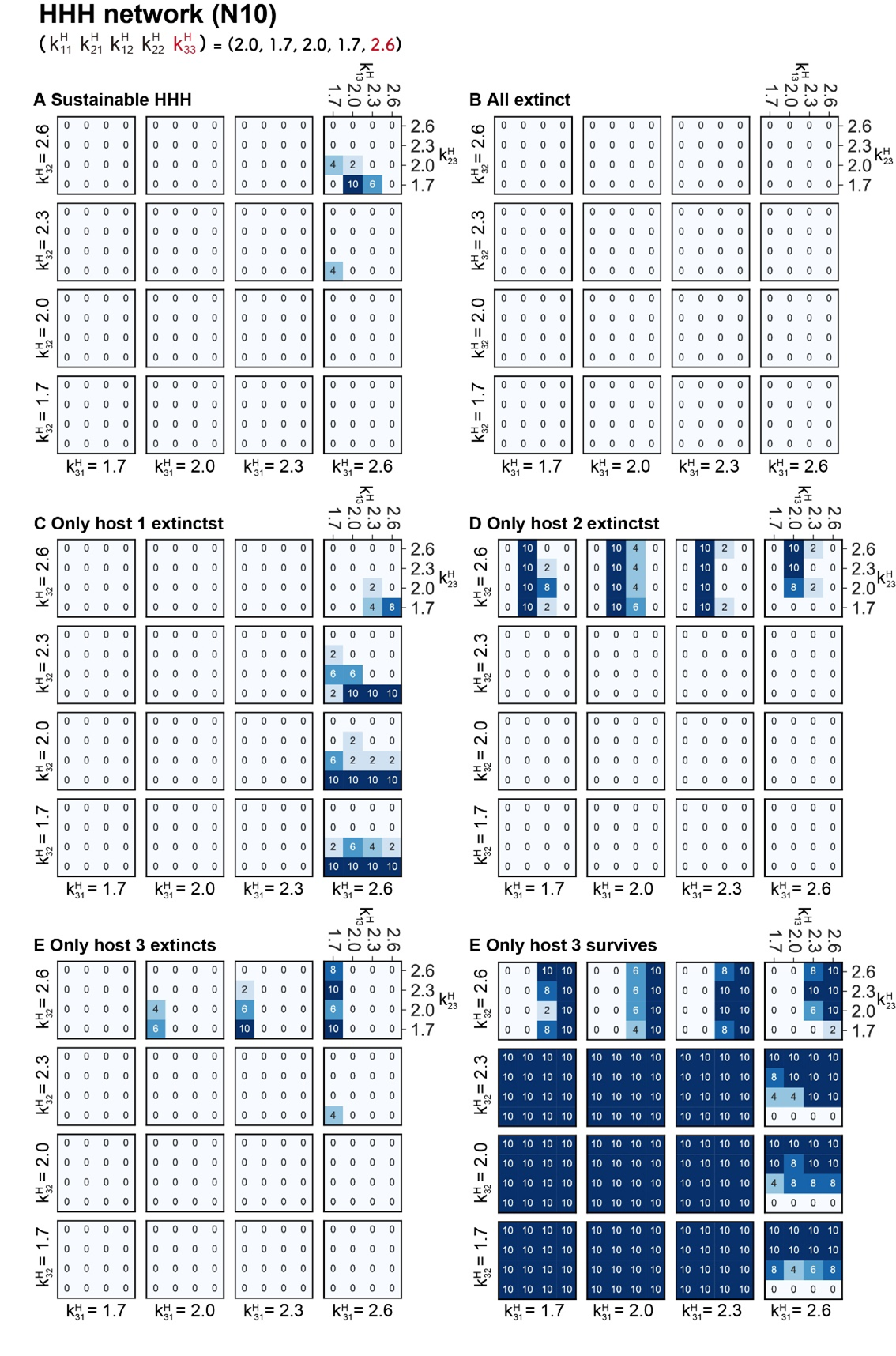

Supplement: S13 Fig — Simulations were conducted as described in Fig 6E for 10 times. (TIF) [file pcbi.1010709.s013.tif]

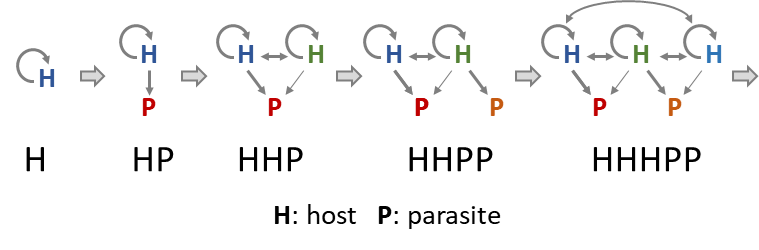

Supplement: S14 Fig — (TIF) [file pcbi.1010709.s014.tif]
