# Supplementary material for: Plausible pathway for a host-parasite molecular replication network to increase its complexity through Darwinian evolution
Source: PLoS Comput Biol. 2022 Dec 1;18(12):e1010709. doi: 10.1371/journal.pcbi.1010709 (PMC9714742; doi:10.1371/journal.pcbi.1010709)
Supplement: S1 Text — (DOCX) [file pcbi.1010709.s017.docx]

**Derivation of eq. 3**

As described in the methods section, the differential equation for host replication is as follows:

$$\frac{dH_{i}}{dt}=H_{i}\left( \sum_{j} k_{ji}^{H}H_{j} \right)\left( 1-\frac{\sum H_{j}+\sum P_{j}}{N} \right)$$

For the two-step experiment conducted in Fig. 9A, replication was stopped before the carrying capacity was reached; thus, the effect of the carrying capacity, $\left( 1-\frac{\sum H_{j}+\sum P_{j}}{N} \right)$, was neglected. Then, we obtained $\frac{dH_{i}}{dt}=H_{i}\left( \sum_{j} k_{ji}^{H}H_{j} \right)$*.* In this equation, the first factor, $H_{i}$, represents the template RNA to be replicated, and the second factor, $\left( \sum_{j} k_{ji}^{H}H_{j} \right)$, represents the total replication enzymes provided by all host RNAs. To estimate $k_{12}^{H}$ and $k_{21}^{H}$, the coefficients of Host^1^_exp_ and Host^2^_exp_, we conducted a two-step reaction, as shown in Fig. 9, in which the replication enzymes were produced from only the first RNA (RNA I), and both RNA I and II were replicated using replicase. For example, for a reaction with host *i* as RNA I and host *j* as RNA II, the rate of host replication in the second reaction should be $\frac{dH_{i}}{dt}=H_{i}\left( k_{ii}^{H}\bar{H_{i}} \right)$ and $\frac{dH_{j}}{dt}=H_{j}\left( k_{ij}^{H}\bar{H_{i}} \right)$, where $\bar{H_{i}}$ represents host *i* concentrations in the first reaction, and thus they are constant values. Solving these equations gives $H_{i}=H_{i}^{t=0}e^{k_{ii}^{H}\bar{H_{i}}}$ and $H_{j}=H_{j}^{t=0}e^{k_{ij}^{H}\bar{H_{i}}}$, where $H_{i}^{t=0}$ and $H_{j}^{t=0}$ represent the concentrations of RNAs *i* and *j*, respectively, before the second reaction. In the two-step replication experiment, we measured the increase in the ratios from 0 to 1 h in the second reaction as fold values (Fig. 9B). We defined the common logarithms of the fold value as $v_{ij}^{h}$, where the subscripts *i* and *j* represent the RNA species used as RNA I and II, respectively. Accordingly, the above equations yield ${v_{ij}^{i}=k}_{ii}^{H}\bar{H_{i}}\log e$ and ${v_{ij}^{j}=k}_{ij}^{H}\bar{H_{i}}\log e$. Therefore, we obtained $k_{ij}^{H}= k_{ii}^{H}\frac{v_{ij}^{j}}{v_{ij}^{i}}$. Similarly, $k_{ij}^{P}= k_{ii}^{H}\frac{v_{ij}^{j}}{v_{ij}^{i}}$ is obtained when the replicator *j* is a parasite.

RNA sequences

> Original host

GGGAACCCCCCUUCGGGGGGUCACCUCGCGCAGCGGGCUACGCGAGGGAGCCACGCUGCGAAGCAGCGUGGCGGUUCUCGUGCGUCACCGAAACGCACGAAGGUCGCGCCUCUUCACGAGGCGUCACCUGGGAGAGCGCGAAAGCGCUAGCCCGUGCUCUAGCUCUAGAAGGUCUCGAGAUCUCCUCUAGAGAUAAUUUUGUUUAACUCUAAGAAGGAGAUAUACACAUGCCUAAGACAGCAUCUUCGCGUAACUCUCUCAGCGCACAAUUGCGCCGAGCCGCGAACACAAGAAUUGAGGCUGAAGGUAACCUCGCACUUUCCAUUGCCAACGAUUUACUGUUGGCCUAUGGUCAGUCGCCAUUUAACUCUGAGGCUGAGUGUAUUUCAUUCAGCCCGAGAUUCGACGGGACCCCGGAUGACUUUAGGAUAAAUUAUCUUAAAGCCGAGAUCAUGUCGAAGUAUGACGACUUCAGCCUAGGUAUUGAUACCGAAGCUGUUGCCUGGGAGAAGUUCCUGGCAGCAGAGGCUGAAUGUGCUUUAACGAACGCUCGUCUCUAUAGGCCUGACUACAGUGAGGAUUUCAAUUUCUCACUGGGCGAGUCAUGUAUACACAUGGCUCGUAGAAAAAUAGCCAAGCUAAUAGGAGAUGUUCCGUCCGUUGAGGAUAUGUUGCGUCACUGCCGAUUUUCUGGCGGUGCUACAACAACGAAUAACCGUUCGUACAGUCAUCCGUCCUUCAAGUUUGCACUUCCGCAAGCGUGUACGCCUCGGGCUUUGAAGUAUGUUUUAGCUCUCAGAGCUUCUACACAUUUCGAUAUCAGAAUUUCUGAUAUUAGCCCUUUUAAUAAAGCAGUUACCGUACCUAAGAACAGUAAGACAGAUCGUUGUAUUGCUAUCGAACCUGGUUGGAAUAUGUUUUUCCAACUGGGUAUCGGUGGCAUUCUACGCGAUCGGUUGCGUUGCUGGGGUAUCGAUCUGAAUGAUCAGACGAUAAAUCAGCGCCGCGCUCACGAAGGCUCCGUUACUAAUAACUUAGCAACGGUUGAUCUCUCAGCGGCAAGCGAUUCUAUGUCUCUUGCCCUCUGUGAGCUCUUAUUGCCCCGAGGCUGGUUUGAGGUUCUUAUGGACCUCAGAUCACCUAAGGGGCGAUUGCCUGACGGUAGUGUUGUUACCUACGAGAAGAUUUCUUCUAUGGGUAACGGUUACACAUUCGAGCUCGAGUCGCUUAUUUUUGCUUCUCUCGCUCGUUCCGUUUGUGAGAUACUGGACUUAGACUCGUCUGAGGUCACUGUUUACGGAGACGAUAUUAUUUUACCGUCCCGUGCAGUCCCUGCCCUCCGGGAAGUUUUUAAGUAUGUUGGUUUUACGACCAAUACUAAAAAGACUUUUUCCGAGGGGCCGUUCAGAGAGUCGUGCGGCAAGCACUACUAUUCUGGCGUAGAUGUUACUCCCUUUUACAUACGUCACCGUAUAGUGAGUCCUGCCGAUUUAAUACUGGUUUUGAAUAACCUAUAUCGGUGGGCCACUAUUGACGGCGUAUGGGAUCCUAGGGCCCAUUCUGUGUACCUCAAGUAUCGUAAGUUGCUGCCUAAACAGCUGCAACAUAAUACUAUACCUGACGGUUACGGUGAUGGUGCCCUCGUCGGAUCGGUCCUAAUCAAUCCUUUCGCGAAAAACCGCGGGUGGAUCCGGUACGUACCGGUGAUUACGGACCAUACAAGGGACCAAGAGCGCGCUGAGUUGGGGUCGUAUCUCUACGACCUCUUCUCGCGUUGUCUCUCGGAAAGUAACGAUGGGUUGCCUCUUAGGGGUCCAUCGGGUUGCGAUUUUGCUGAUCUAUUUGCCAUCGAUCAGCUUAUCUGUAGGAGUGAUCCUACGAAGAUAAGCAGGCCUACCGGUAAAUUCGAUAUACAGUACAUCGCGUGCUGUUGUUCGGGUUGUUGUUAGGCUUGCGGCCGCACUCGAGAGAUCUAGAGCAUCACGGUCGAACUCCCGUACGAGGUGCCCGCACCUCGUCCCCCCCUUCCGGGGGGGUCCCC

> Representative Host^1^exp

GGGAACCCCCCUUCGGGGGGUCACCUCGCGCAGCGGGCUACGCGAGGGAGCCACGCUGCGAAGCAGCGUGGCGGUUCUCGUGCGUCACCGAAACGCACGAAGGUCGCGCCUCUUCGCGAGGCGUCACCUGGGAGAGCGCGAAAGCGCUAGCCCGUGCUCUAGCUCUAGAAGGUCUCGAGAUCUCCUCUAGAGAUAAUUUUGUUUAACUCUAAGAAGGAGAUAUACACAUGCCUAAGACAGCAUCUUCGCGUAACUCUCUCAGCGCACAAUUGCGCCGAGCCGCGAACACAAGAAUUGAGGCUGAAGGUAACCUCGCACUUUCCAUUGCCAACGAUUUACUGUUGGCCUAUGGUCAGUCGCCAUUUAACUCUGAGGCUGAGUGUAUUUCAUUCAGCCCGAGAUUCGACGGGACCCCGGAUGACUUUAGGAUAAAUUAUCUUAAAGCCGAGAUCAUGUCGAAGUAUGACGACUUCAGCCUAGGUAUUGAUACCGAAGCUGUUGCCUGGGAGAAGUUCCUGGCAGCAGAGGCUGAAUGUGCUUUAACGAACGCUCGUCUCUAUAGGCCUGACUACAGUGAGGAUUUCAAUUUCUCACUGGGCGAGUCAUGUAUACACAUGGCUCGUAGAAAAAUAGCCAAGCUAAUAGGAGAUGUUCCGUCCGUUGAGGAUAUGUUGCGUCACUGCCGAUUUUCUGGCGGUGCUACAACAACGAAUAACCGUUCGUACAGUCAUCCGUCCUUCAAGUUUGCACUUCCGCAAGCGUGUACGCCUCGGGCUUUGAAGUAUGUUUUAGCUCUCAGAGCUUCUACACAUUUCGAUAUCAGAAUUUCUGAUAUUAGCCCUUUUAAUGAAGCAGUUACCGUACCUAAGAACAGUAAGACAGAUCGUUGUAUUGCUAUCGAACCUGGUUGGAAUAUGUUUUUCCAACUGGGUAUCGGUGGCAUUCUACGCGAUCGGUUGCGUUGCUGGGGUAUCGAUCUGAAUGAUCAGACGAUAAAUCAGCGCCGCGCUCACGAAGGCUCCGUUACUAAUAACUUAGCAACGGUUGAUCUCUCAGCGGCAAGCGAUUCUAUGUCUCUUGCCCUCUGUGAGCUCUUAUUGCCCCGAGGCUGGUUUGAGGUUCUUAUGGACCUCAGAUCACCUAAGGGGCGAUUGCCUGACGGUAGUGUUGUUACCUACGAGAAGAUUUCUUCUAUGGGUAACGGUUACACAUUCGAGCUCGAGUCGCUUAUUUUUGCUUCUCUCGCUCGUUCCGUUUGUGAGAUACUGGACUUAGACUCGUCUGAGGUCACUGUUUACGGAGACGAUAUUAUUUUACCGUCCCGUGCAGUCCCUGCCCUCCGGGAAGUUUUUAAGUAUGUUGGUUUUACGACCAAUACUAAAAAGACUUUUUCCGAGGGGCCGUUCAGAGAGUCGUGCGGCAAGCACUACUAUUCUGGCGUAGAUGUUACUCCCUUUUACAUACGUCACCGUAUAGUGAGUCCUGCCGAUUUAAUACUGGUUUUGAAUAACCUAUAUCGGUGGGCCACUAUUGACGGCGUAUGGGAUCCUAGGGCCCAUUCUGUGUACCGCAAGUAUCGUAAGUUGCUGCCUAAACAGCUGCGACAUAAUACUAUACCUGACGGUUACGGUGAUGGUGCCCUCGUCGGAUCGGUCCUAAUCAAUCCUUUCGCGAAAAACCGCGGGUGGAUCCGGUACGUACCGGUGAUUACGGACCAUACAAGGGACCAAGAGCGCGCUGAGUUGGGGUCGUAUCUCUACGACCUCUUCUCGCGUUGUCUCUCGGAAAGUAACGAUGGGUUGCCUCUUAGGGGUCCAUCGGGUUGCGAUUUUGCUGAUCUAUUUGCCAUCGAUCAGCUUAUCUGUAGGAGUGAUCCUACGAAGAUAAGCAGGCCUACCGGUAAAUUCGAUAUACAGUACAUCGCGUGCUGUUGUUCGGGUUGUUGUUAGGCUUGCGGCCGCACUCGAGAGAUCUAGAGCAUCACGGUCGAACUCCCGUACGAGGUGCCCGCACCUCGUCCCCCCCUUCCGGGGGGGUCCCC

> Representative Host^2^exp

GGGAACCCCCCUUCGGGGGGUCACCUCGCGCAGCGGGCUACGCGAGGGAGCCACGCUGCGAAGCAGCGUGGCGGUUCUCGUGCGUUACCGAAACGCACGAAGGUCGCGCCUCUUCACGAGGCGUCACCUGGGAGAGCGCGAAAGCGCUAGCCCGUGCUCUAGCUCUAGAAGGUCUCGAGAUCUCCUCUAGAGAUAAUUUUGUUUAACUCUAAGAAGGAGAUAUACACAUGCCUAAGACAGCAUCUUCGCGUAACUCUUUCAGCGCACAAUUGCGCCGAGCCGCGAACACAAGAAUUGAGGCUGAAGGUAACCUCGCACUUUCCAUUGCCAACGAUUUACUGUUGGCCUAUGGUCAGUCGCCAUUUAACUCUGAGGCUGAGUGUAUUUCAUUCAGCCCGAGAUUCGACGGGACCCCGGAUGACUUUAGGAUAAAUUAUCUUAAAGCCGAGAUCAUGUCGAAGUAUGACGACUUCAGCCUAGGUAUUGAUACCGAAGCUGUUGCCUGGGAGAAGUUCCUGGCAGCAGAGGCUGAAUGUGCUUUAACGAACGCUCGUCUCUAUAGGCCUGACUACAGUGAGGAUUUCAAUUUCUCACUGGGCGAGUCAUGUAUACACAUGGCUCGUAGAAAAAUAGCCAAGCUAAUAGGAGAUGUUCCGUCCGUUGAGGAUAUGUUGCGUCACUGCCGAUUUUCUGGCGGUGCUACAACAACGAAUAACCGUUCGUACAGUCAUCCGUCCUUCAAGUUUGCACUUCCGCAAGCGUGUACGCCUCGGGCUUUGAAGUAUGUUUUAGCUCUCAGAGCUUCUACACAUUUCGAUAUCAGAAUUUCUGAUAUUAGCCCUUUUAAUAAAGCAGUUACCGUACCUAAGAACAGUAAGACAGAUCGUUGUAUUGCUAUCGAACCUGGUUGGAAUAUGUUUUUCCAACUGGGUAUCGGUGGCAUUCUACGCGAUCGGUUGCGUUGCUGGGGUAUCGAUCUGAAUGAUCAGACGAUAAAUCAGCGCCGCGCUCACGAAGGCUCCGUUACUAAUAACUUAGCAACGGUUGAUCUCUCAGCGGCAAGCGAUUCUAUGUCUCUUGCCCUCUGUGAGCUCUUAUUGCCCCGAGGCUGGUUUGAGGUUCUUAUGGACCUCAGAUCACCUAAGGGGCGAUUGCCUGACGGUAGUGUUGUUACCUACGAGAAGAUUUCUUCUAUGGGUAACGGUUACACAUUCGAGCUCGAGUCGCUUAUUUUUGCUUCUCUCGCUCGUUCCGUUUGUGAGAUACUGGACUUAGACUCGUCUGAGGUCACUGUUUACGGAGACGAUAUUAUUUUACCGUCCCGUGCAGUCCCUGCCCUCCGGGAAGUUUUUAAGUAUGUUGGUUUUACGACCAAUACUAAAAAGACUUUUUCCGAGGGGCCGUUCAGAGAGUCGUGCGGCAAGCACUACUAUUCUGGCGUAGAUGUUACUCCCUUUUACAUACGUCACCGUAUAGUGAGUCCUGCCGAUUUAAUACUGGUUUUGAAUAACCUAUAUCGGUGGGCCACUAUUGACGGCGUAUGGGAUCCUAGGGCCCAUUCUGUGCACCUCAAGUAUCGUAAGUUGCUGCCUAAACAGCUGCAACAUAAUACUAUACCUGACGGUUACGGUGAUGGUGCCCUCGUCGGAUCGGUCCUAAUCAAUCCUUUCGCGAAAAACCGCGGGUGGAUCCGGUACGUACCGGUGAUUACGGACCAUACAAGGGACCAAGAGCGCGCUGAGUUGGGGUCGUAUCUCUACGACCUCUUCUCGCGUUGUCUCUCGGAAAGUAACGAUGGGUUGCCUCUUAGGGGUCCAUCGGGUUGCGAUUUUGCUGAUCUAUUUGCCAUCGAUCAGCUUAUCUGUAGGAGUGAUCCUACGAAGAUAAGCAGGCCUACCGGUAAAUUCGAUAUACAGUACAUCGCGUGCUGUUGUUCGGGUUGUUGUUAGGCUUGCGGCCGCACUCGAGAGAUCUAGAGCAUCACGGUCGAACUCCCGUACGAGGUGCCCGCACCUCGUCCCCCCCUUCCGGGGGGGUCCCC

> Representative Parasite^1^_exp_

GGGAACCCCCCUUCGGGGGGUCACCUCGCGCAGCGGGCUGCGCGAAGGAGCCACGCUGCGAAGCAGUGUGGCGGUUCUCGUGCGUUACCGAAACGCACGAAGGUCGCGCCUCUUCACGAGGCGUCACCUGGGAGAGCGCGAAAGCGCUAGCCCGUGAUUCGUCACGGUCGAACUCCCGUACGAGGUGCCCGCACCUCGUCCCCCCCUUCCGGGGGGGUCCCC
