# Supplementary material for: Plausible pathway for a host-parasite molecular replication network to increase its complexity through Darwinian evolution
Source: PLoS Comput Biol. 2022 Dec 1;18(12):e1010709. doi: 10.1371/journal.pcbi.1010709 (PMC9714742; doi:10.1371/journal.pcbi.1010709)
Supplement: Attachment — Submitted filename: report.pdf [file pcbi.1010709.s019.pdf]

Dear Authors, dear Editor:

I have now read and assessed the manuscript titled “Plausible pathway for a host-parasite molecular replication network to increase its complexity through Darwinian evolution”. I think that this paper contains very interesting science, and I hope that some version of it is published in a very good journal. To do so, I think that the Authors need to tackle some major issues that affect the work.

These issues range from the methodological to the interpretation of the results and experiments. I think that some issues must necessarily be tackled in order to attain a version of the manuscript that can be published. On the other hand, some conflicting points seem to arise from a different understanding of the dynamics that happen during the experiments. The Authors seem to write from a “population dynamics” perspective, which is grounded on currently accepted views of Darwinian evolution. It is my opinion that such views are incomplete and, crucially, that the “population dynamics”-limit is reached under conditions that, I think, are not met in this experiment. As I will explain, this causes problems in the interpretation of the experiments.

I acknowledge that these later issues are a matter of opinion and interpretation. It would not be fair for me to discredit, on such basis, the great scientific work done by the Authors. Furthermore, I would expect that most reviewers with a strong biological background would accept the “population dynamics” account by the Authors without hesitation. However, I think that the conflict between interpretations raises some important questions, and that the paper would be greatly improved if these issues were tackled or at least discussed.

In this manuscript the Authors combine computational and experimental methods to asses how a small ecosystem might increase its complexity over time. The ecosystems studied consist of hosts (which might cooperate or compete with each-other) and parasites (which benefit from hosts but do not help them in return). The ecosystem dynamics are encoded by logistic equations whose coefficients determine how intense competition and cooperation are. The Authors study numerically the stability of equations for several values of the coefficients, thus finding combinations of parameters for which host-host, host-parasite, host-host-host, and host-parasite-host configurations are stable. They find that adding a parasite increases the ranges of parameters leading to stable coexistence, and thus determine that the most likely route for the ecosystem to gain complexity is, starting from a single host, to then acquire a parasite, and then yet another host. The Authors speculate that this mechanism could be iterated for larger ecosystems to gain parasitic and host species alternately. In a final, important part of the paper, the Authors compare this route to complexification with results from earlier experiments that they had performed. They conclude that the proposed route was observed in their previous data, and perform new experiments to argue that the final configuration (allegedly, with two hosts and a parasite) is stable.

As pointed out above, several issues exist that affect critical phases of the chain of reasoning presented by the Authors.

On the one hand, the methodology for the numerical experiments is not completely clear, and, in my opinion, leaves the door open to the possibility that the Authors are not assessing the stability of the system correctly. This requires a lot of clarification. Methodological problems also affect the only one simulation presented to show the route to complexification (which I will note HPH for short) in action.

On the other hand, I have some serious issues with interpreting that HPH is what is actually going on in the experiments. These issues are mostly derived from my interpretation of Darwinian dynamics in these experiments. These issues make me doubt the interpretation that their configuration is stable, as the Authors attempt to argue with their new experiments.

In the following, I present bullet points with specific issues. These points are presented in a section-wise manner: Introduction, Methods, Results. Within each section, bullets appear in the same order as in the text.

Introduction:

- Abstract (lines 42-45) and lines 59-61:
  - I think that it is important to discuss what processes (including Darwinism) led to the complexification of prebiotic chemistry. However, in the way that the Authors frame this issue, it generates the expectation that the paper will make more explicit contributions towards clarifying this crucial step.

- In lines 96-98:
  - Introducing for the first time our different interpretations of the dynamics: Note that competition among RNA species as described by population dynamics (which leads to competitive exclusion) is attained in a certain limit. Among the conditions to reach this limit is that species are well delimited. This is not the case in the presented experiments, in which broad coexistence between RNA-sequence variants is persistently observed—contradicting competitive exclusion. These apparent issues disappear if we adopt a quasispecies interpretation of the experiments.

- I think that the following papers might be important for this work:
  - Seoane LF, Solé R. Information theory, predictability and the emergence of complex life. *R. Soc. Open Sci.*, **5**(2), p.172221 (2018).
  - Seoane LF, Solé R. How Turing parasites expand the computational landscape of digital life. arXiv preprint arXiv:1910.14339.
  - Hickinbotham SJ, Stepney S, Hogeweg P. Nothing in evolution makes sense except in the light of parasitism: evolution of complex replication strategies. *R. Soc. Open Sci.*, **8**(8), p.210441 (2021).

Methods:

I have two main issues with the computational methods of this paper. On the one hand, I do not feel very satisfied with the number of replicas carried out for each combination of parameters. On the other hand, the description of the simulations is rather obscure. There are some parts that I could not understand. Actually, my attempts to reproduce some critical steps led me to dynamics that might artificially alter the stability that the Authors attempt to study.

Regarding the number of replicas: The model described is relatively simple, and it should be possible to run even thousands of instances for each combination of parameters within a very reasonable time. Furthermore, the Authors write that a huge amount of the simulations do not even make it pass the first round of Replication, Selection, and Fusion-Division. I think that the amount of replicas (10 or even 3 in some cases) is very low for a computational study. Below, I interleave some ideas that might help speed up the simulations.

First, in this regard (and perhaps I am mistaken), might the Authors be using a very slow programming language? Languages such as Python or R, which are often used in implementing computational biological models, are often thousands of times slower than lower-level languages such as C++ or ForTran.

A list of points that should be addressed follows:

- In lines 459-460:
  - There are three sums in each equation. Two of these sums (those of hosts) run over the same sets, while the sum over parasites runs over a different set. Perhaps it would help to use different indexes for hosts and parasites.

- Similarly, two different sets of k_{ij} appear: One set links hosts to hosts and another one links hosts to parasites. It would make things easier for the reader if the difference between these two sets were explicitly marked by using different symbols. As the equations are written, we could assume that a same number of hosts and parasites exist, and that they have the same coefficients k_{ij}.
- A similar comment applies to the left-hand-side of these equations: By labeling both hosts and parasites with i, it seems that they belong in a same set—which is not the case.

- In lines 463 and 469-470:
  - "N is the carrying capacity of each compartment." and "The sizes of all compartments are the same.". I hence assume that N is the same for all compartments, but this is not explicitly stated and both sentences are far apart (the connection might get lost).

- In lines 472-475:
  - After C compartments have been simulated, only C_S of them are used. This means that $C-C_S$ compartments have been simulated only to be discarded. Since the selection rate is $S=0.25$, this means that the Authors are discarding 3 out of every 4 simulations that they run. If, instead of discarding compartments after the replication phase, they would do it before, then the number of replicas of the simulations could be increased 4-fold with the same current setup (i.e., there would be up to 40 or 12 repetitions for each combination of parameters).
  - It is stated that "empty compartments were supplied up to the fixed total number of compartments". I assume that $C-C_S$ empty compartments are supplied, but this is not clear from the text. In my first reading I assumed that C new empty compartments had been provided, and that they would be filled from combinations of earlier compartments. I am still not convinced that this second interpretation is not the right one—if it were, it would clarify some of the following points.

- In lines 477-483:
  - This paragraph is particularly obscure in the details. I doubt that the exact same experiment would be reproduced by a reader. Many details are missing. Also, depending on some of these details, it might be possible to increase the efficiency of the code (resulting in more replicas for each combination of parameters).
    - Assuming $C-C_S$ empty compartments were provided and a selection rate of $S=0.25$, at the beginning of the fusion-division phase most of the compartments are empty. In my best understanding of this paragraph, two compartments are chosen randomly. It is most likely that these two compartments will be empty—thus the Authors are combining empty compartments very often, which is inefficient. Back to the pipeline, the two selected compartments are combined and then (I think?) returned to the pool. This processes is repeated $A=3000$ times.
    - I have simulated a similar, yet simplified process. I initialized $C_S=750$ compartments with random numbers and 2250 empty compartments. Then, I mixed the content of randomly chosen couples of compartments by taking the average, a process which I repeated $A=5000$ times. After one such process, it was typical that ~ 280 compartments would remain empty. Then, $C_S=750$ of the total compartments would be selected for a next round, carrying over some of the empty compartments. The pool would be filled up with 2250 new additional empty compartments. I have tracked the number of empty compartments over time and found the dynamics in the plots below.
    - There are two reasons why the number of empty compartments changes over time (eventually, leading to an "all empty" state). On the one hand, each round, a set of empty compartments can be selected and increase the number of empty

        compartments in the next round. On the other, mixing non-empty with empty compartments tends to dilute the concentrations over time. This last problem should not affect so much the dynamics simulated in the manuscript, as diluted concentrations can come back up during the replication step (but I am not so sure about this, since certain interpretations of the experiments could actually worsen this problem). In any case, I am not so sure that these issues do not change the likelihood of ending up with empty compartments. My main worry is that the implemented dynamics are not only affected by the stability of the equations, but perhaps also by a highly ad-hoc resampling process.
        - I assume that this model attempts to replicate the wet-lab experiments from which the empirical data is obtained. It would be useful to have a succinct description of these experiments to see how the different phases of the model match up with the experiments.
    - This paragraph still presents some additional problems:
        - In line 480-481: What does it mean precisely that "the number of replicators in the compartment was approximated from the concentrations"?
        - In lines 481-483—specially troublesome:
            - How are the replicators redistributed randomly into two new compartments? Is this decided in a one by one case for each of the replicators (e.g. for each replicator a coin is flipped)? (Note that the implementation of flipping a coin for each replicator would be very slow. This could contribute towards inefficiency and low number of replicas.)
            - The two compartments into which the replicators are redistributed: are they the same as the ones that got mixed up? Or are they two new randomly selected compartments from the pool? If so, are the two original compartments emptied when the replicators are redistributed to the new compartments? If not, at this step the number of replicators is being artificially multiplied by 2.
            - I have run simulations of several variations of my toy mixture model taking into account the possible answers to these questions. I have observed transitions to "all empty" (as in the plot below) in all cases except when the number of replicators is artificially duplicated.

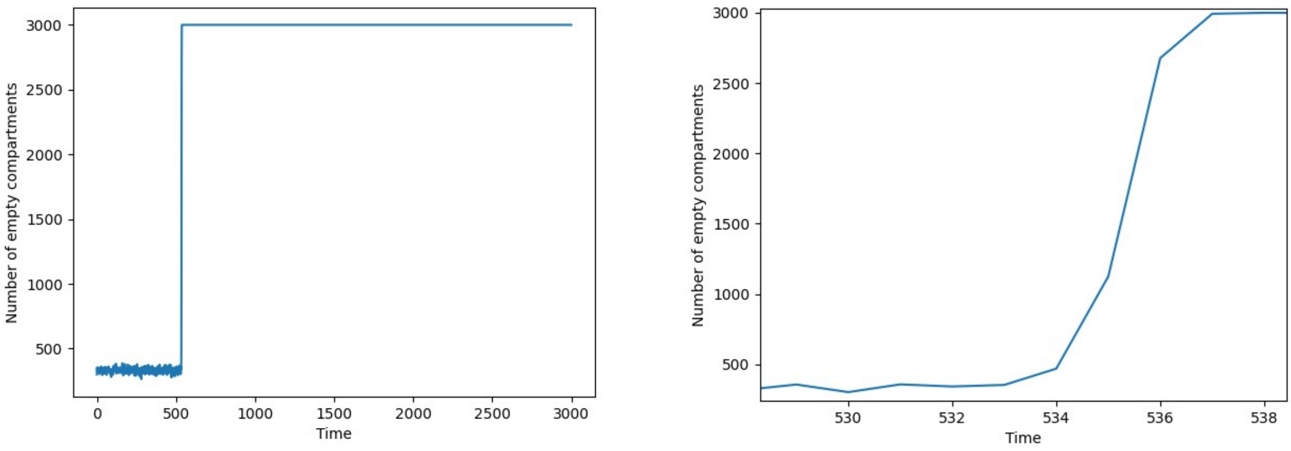

- In lines 503-505:
    - Values of the coefficients are listed. It does not hurt to indicate explicitly which are the *k_ij* for hosts and the *k_ij* for parasites, and (as commented above) to make these symbols different.

Results:

- In lines 151-152:
    - I assume that, in this sentence, the Authors refer to the experiment described in section “Parameter estimation of the representative RNAs”, at the end of the manuscript. It would help if this section were mentioned explicitly. This manuscript bases a lot of their results in earlier experiments (from Refs. [29-31]), and it sometimes becomes difficult to track what comes from there.

- In line 176:
    - As in the previous point. It would help the reader if explicit references existed to the papers or sections where these parameters were obtained.

- In lines 187-189:
    - I am sorry, I don’t understand this sentence.

- Fig. 4:
    - I think that this figure can be eliminated.

- In line 221:
    - Watch out for subscripts.

- In Fig. 5:
    - Is panel D necessary?

- In section “Computer simulation of the transition of the networks”:
    - I find this section very confusing. On the one hand, I think that it is not well explained and that these simulations might be affected by the issues mentioned before—regarding sampling, division-fusion, etc. On the other hand, I do not understand the results, or I think that the obtained results seem meager.
    - In lines 303-305:
        - These lines are very confusing. I don’t know what is a round, a run, and a time.
        - I find it very odd that all replicators die off in 97 (out of 100?) runs. I think that the implementation of the dynamics (as discussed in my issues with the Methods section) might be playing a very crucial role.
        - Can we sustain the claim that HPH is a pathway to complexification if 97 out of 100 replicas simply died?
    - In Fig. 7:
        - According to the methods (line 498), hosts mutate with a chance 0.5 such that, at each iteration, half of the existing hosts should become a new host. But we do not see mutants popping up at each iteration. On the one hand, such a high mutation rate is not realistic if we wanted to remain in the “population dynamics” limit. On the other hand, what is going on with the simulations at this point? Why don’t we observe the maximum number of species (3) in every round?
        - Also from the Methods (lines 499-500) and Results (lines 298-300), “the total number of replicator species is restricted to less than three”. Might this be what is limiting, artificially, the appearance of new species and alternative routes to stability? If new mutants are prevented from popping up, we are biasing the system towards HPH for complexification. Indeed, we observe HH coexisting for hundreds of rounds. We even observe HHH for dozes of rounds, if I’m interpreting Fig. 7 correctly. Shouldn’t these configurations be rather unstable?

- Some limitations and comments about this simulation:
  - I understand that the authors limited the species to 3 for computational issues (it becomes unfeasible to study the stability of their model as more species are introduced).
  - On the other hand, I understand that they needed to limit the number of parameter values that they evaluated in order to asses the stability. However, this later point is not necessary when simulating the dynamics in this section. Actually, as the experiments make very clear (and I will discuss below) it is reasonable that coefficients for new hosts and parasites will not take values over the discrete set of parameters, but over the continuum in which parameters live. Actually, through Darwinian selection, it is more likely that sets of parameters will be observed within the region where dynamics are stable.
  - If we remove these artificial constraints, the picture in which the HPH route to complexification is preferred does not seem so clear. With such high mutation rates, it is more likely that many more hosts and parasites emerge and decay at every time-step. If the mutation rate were lower, it remains very likely that stable HH ecosystems form following Darwinian elimination of unstable host partners.
  - All in all, I am not convinced that this simulation strengthens the hypothesis that HPH is the most reliable route to complexification—most of all because it is based on 1 out of 3 simulations versus the 97 simulations that did not even remain stable.

- In section "Host and parasitic RNAs that may form HP and HHP networks in the previous evolutionary experiments":
  - In this section, it shows up my greatest discrepancy with how the Authors interpret their experiments. They seem to adopt a "population dynamics" perspective (and I apologize if I am wrong about this point). In turn, Fig. 8 strongly suggests, in my opinion, that we are looking at a quasispecies dynamic. It is difficult for me to view each sequence as a different species of host or parasite popping up into existence, and doing so feels like attempting to force the data into the computational model.
  - What we seem to have, instead, is a cloud of mutants that are accessible from each-other as connected in a genotype-genotype network. Hosts are at distances 3 and 4 from the original RNA sequence, and they are at only 7 mutations from each other. Meaning that both hosts should be relatively accessible from each other. What are the mutation rates of the sequences in this experiment? How many unique sequences are observed in each round? From my experience with similar experiments, I bet that mutation rates are very high and that there are a lot of different unique sequences. I further bet that there, in the population, a lot of the intermediate steps between both hosts are abundantly represented. Meanwhile, the hosts are at a distance 11 in the dendogram. Does this make sense, provided they are only 7 mutation apart? This suggests that a phylogeny is not appropriate to observe this kind of Darwinian process—rather, as I said, a genotype-genotype network seems more appropriate.
  - If this view is adopted, several of the preceding issues disappear. For example, there is no competitive exclusion because each sequence is generating nearby neighbors in the network by frequent mutation. Viable mutants are fairly close to their ancestors, thus they naturally contribute to cross-reproduction as modeled by the k_{ij} between different hosts. Also, coefficients of new mutants might be close to older ones, favoring stable configurations that are selected in a Darwinian fashion. Note that, if we insist on the population dynamics perspective, the experiments show rather stable coexistences between several hosts, which should be unstable according to the stability analysis. Do the Authors believe that there is a parasite in Fig. 8 for each possible host, and that they have been introduced as reported in Sup. Fig. S5? Isn't it much more parsimonious to

assume that we are looking at a quasispecies and that a broad range of parasitic strains is sustained together with a similarly broad range of able strains?

  - As I said above, I am very sympathetic to this work and I hope that some version of it gets published in a very good journal. Unfortunately, I have the strong feeling that the Authors are forcing a partial interpretation of Darwinian dynamics that, I think, simply does not apply in this experiment. The limit in which population dynamics applies has no crossover between competing species, while here it is obvious that one host can be reached from the other in a few rounds of the experiment.

- In Fig. 9:
  - In a final comment regarding the population dynamics versus quasispecies interpretation of the data:
    - The experiment reported in Fig. 9 runs for 25 rounds. In Fig. 8B, that seems to be the time that it took the original RNA strain to jump from the ancestral branch to branch 2. (This is only apparent, since we do not have access to intermediate samplings of the experiment.) Still in Fig. 8B, we then observe a fast diversification in rounds 33 and 39, which is less time than the 25 previous rounds.
    - I find it very likely that a similar phenomenon might go on, should the experiment in Fig. 9 be continued for a few more rounds. Furthermore, while the stability is attributed to the HPH configuration, I think that it is more likely that both hosts and the parasite are actually accompanied by an unobserved cloud of mutants, and that their stability stems from quasispecies dynamics. These hosts and parasite, in principle, already belonged to a rather mature quasispecies, which they might have been able to recreate.
