# Supplementary material for: Plausible pathway for a host-parasite molecular replication network to increase its complexity through Darwinian evolution
Source: PLoS Comput Biol. 2022 Dec 1;18(12):e1010709. doi: 10.1371/journal.pcbi.1010709 (PMC9714742; doi:10.1371/journal.pcbi.1010709)
Supplement: Attachment — Submitted filename: report2.pdf [file pcbi.1010709.s021.pdf]

Dear Authors, dear Editor:

I have now read the revised version of this manuscript, as well as the detailed responses by the Authors, and the report by the other Referee. There are several points about this manuscript that I find troublesome. Much of the disagreement comes from different interpretations of the experimental data, but I do not think that it is fair to use my leverage as a Referee to attempt to impose a viewpoint. I also feel that these issues will not be resolved by any amount of further reviewing. I add comments about these issues below, hoping that they might improve our understanding of the experiments and evolutionary dynamics in general. Apart from these points in question, I think that the work is mostly technically sound.

There are other aspects of the paper with which I take issue, and which, I think, can be solved with further minor editing. My comments on this respect are attached below as well, hoping that they will be addressed before a foreseeable publication of the paper.

I hope that my comments are useful in improving the manuscript.

Best regards.

Major concerns:

The reference indicated by the other Referee (Takeuchi & Hogeweg, 2008) takes away a great part of the novelty that I though this work presented. I was not familiar with that paper before. Honestly, I have had manuscripts rejected for much less overlap with earlier research.

However, I feel that rejecting on such a basis would be unfair. Things are re-discovered all the time, and the literature is often too vast to know every piece of work. I do take issue with the fact, pointed out by the other Referee, that the Authors seemed to misrepresent or diminish the findings by Takeuchi and Hogeweg in the earlier version. That could not come from a lack of familiarity with their paper. However, willingly or not, this has now been corrected.

Once that that part of the novelty has been somehow taken away, we are left with the actual contribution of this manuscript: To work out mathematically, and with allegedly realistic parameters, a situation that has purportedly taken place in the reported experiment. Unfortunately, I do not think that what the Authors model is what has happened in their experiment at all. Again, this is a matter of interpretation, and we might not have got sufficient empirical evidence to settle the question.

I think that the modeling effort and the discussion is illuminating, and that it should be available for future debate. I think that the mechanism proposed by Takeuchi and Hogeweg, and modeled here, might be operating at some scale in evolutionary dynamics. I also think that some (much more complex) version of this mechanism might be operating within the evolutionary history of this experiment. However, I do not think that the empirical evidence can support that the reductionist view presented here is what has indeed happened in the experiment.

To tackle my earlier issues regarding interpretation, the Authors say that they have now included a discussion in terms of quasispecies. That is not true. The Authors have made some superficial comments about quasispecies—not a discussion of the experiment from this framework. It would be unfair to ask the Authors to include a discussion with which they are not comfortable—in any case, I think that this was a nice opportunity that has been missed.

Focusing on Fig. 8, very likely we do have a huge cloud of mutants around few, very fit RNA strains. That would be a unique quasispecies. It is a very big simplification to take an individual strain as a representative of each branch, and propose that we have seen a sequence H $\rightarrow$ HP $\rightarrow$ HPH. Within each branch, we do have very abundant nearby representatives. The crossed replication coefficients in Fig. 9 are of a similar order of magnitude as the auto-replication

coefficients—and this is for representatives across branches, just imagine how large the contribution of nearby mutants might be. What is more likely going on is that vast clouds of host- and parasite-mutants sprawl from very early and simultaneously. This would result, almost from the beginning, in large networks including host- and parasite-variants that coexist over time—in a well sustained coexistence, indeed. Then, selection would operate, potentially reducing the abundance of variants into more stable subsets. This picture is radically different from the $H \rightarrow HP \rightarrow HPH$ pathway—which appears meager from this perspective. This is what I would expect from a discussion of the experiment in terms of quasispecies. The comments by the Authors do not address any point from this picture.

Let us run some numbers from the data on mutation rate that the Authors have provided. According to empirical data, we would have ~0.02 mutations in each single sequence replication (focusing on hosts only). The experiment has 10^8 compartments. A strain that is present in all compartments would thus generate 2 million strings with mutations after replicating only once (let us call this “one replication step”). (Note that, likely, there are more than one single strain in each compartment. The variety that I calculate is thus a very low estimate.) The resulting mutated strings are likely to have one mutation only, thus they cover a fraction of genotype space at distance 1. Each Replication Cycle of the experiments (Fig. 2B) very likely contained several such replication steps (is there an estimate of this number?). Each such replication step would generate such huge variety (~2 million mutated variants, at the very least), and this novelty will be compounded with each step within one Replication Cycle. Mutations from the second generation are unlikely to walk back, thus their descendants will likely be at distance 2 from the original sequence. It is enough to have 7 replication steps within each Replication Cycle (which seems possible) to be able to explore a neighborhood at distance 7 of the original sequence.

I think that the criterion used by the Authors to argue that their two branches would not belong to a same quasispecies is flawed. Genotype space is vast, and very unlikely to be covered by any cloud of mutants. This does not mean that parts of that space are not mutually accessible in a few steps. The second host (H_2) is definitely accessible in a very short time from the first host (H_1). How did H_2 evolve? Given Fig. 8B, I see two distinct possibilities: i) The whole branch 2 is actually present throughout the experiment (only not at detectable concentrations after round 24). This would add up to the interpretation that we have a single quasispecies at any time, with variants that can spring to prominence at any moment. ii) Within a few replication steps, H_2 evolved from H_1, thus illustrating the fact that both branches are mutually accessible. This would also point out why dendrograms might not be the best ceoncptualization (since H_2 in this case would evolve from H_1, not from others in branch 2).

This reflection is included just as a speculation, to illustrate the rich picture that is left out. Again, I do not think that we will bridge the gulf that separates or views, and I still think that the scientific work in this paper is sound (specially the experiments) and that the discussion can still be useful (my view might be the incorrect one after all).

However, I wanted to end this part of the review with a final comment. In their response, the Authors wrote: “However, we would like to emphasize here that the two different interpretations are not the focus of this study, nor do they affect the conclusion of this study, a plausible route of host and parasite replicator network. As the reviewer noted, this was a matter of interpretation.” I agree that the two interpretations are not the focus of the study. I disagree strongly with the statement that they do not affect the conclusion of this study. Very likely, the route to complexification in the experiment is not what the Authors have modeled. The framework adopted does affect the probability that the $H \rightarrow HP \rightarrow HPH$ has actually taken place. Take the last sentence of the Introduction: “We confirmed that the most plausible pathway (the addition of a parasite, followed by the addition of a parasite-resistant host) occurred in our previous evolutionary experiment.” I am sorry, this has very likely not happened.

Minor concerns:

While the manuscript has improved notably, there are some parts which I still find obscure. I think that the paper would turn out better if they were addressed, if the Authors wished to do so:

- In the abstract, the Authors wrote: “These results provide experimental evidence that a single replicator spontaneously develops into multi-replicator networks through coevolution with parasitic replicators, which might be the first complexification step toward the emergence of life.”
    - In my first report, I took issue with this sentence. The Authors replied: “As per our understanding, the reviewer’s concern is the origin of a new RNA species that forms replicator networks.”, then proceeded to reply to anything but my main concern.
    - No, that was not my issue. My trouble with this sentence is that it suggests that the paper will deal with the emergence of life (as clearly stated in that sentence), and that is not a topic discussed in this paper at all. I feel that this intends to mislead the reader, honestly.

- The first paragraph of the first subsection within Methods might be improved:
    - The first sentence does not make much sense.
    - In line 547, these are “differential” equations.

- I think that the Authors have improved much on the description of the technical aspects. But some issues remain:
    - What are the units of H_i and P_h? The Authors say that they are concentrations: Why not operate with numbers straight away? Logistic equations are perfectly valid if we let these numbers vary over a continuum.
    - This is important because: The Authors say that H_i and P_h are concentrations, and that, upon fusion, they are added. What does it mean precisely to add up concentrations? Concentrations are intensive quantities. Merging together two substances of a same size that contain a concentration of 3 parts per million of RNA each does not result in a concentration of 6 parts per million.
    - My intuition is that these equations represent numbers of copies of each host or parasite, not concentrations. In that case, the procedure discussed by the Authors makes sense. The need to round off these numbers would disappear if, instead of flipping a coin for each molecule, they would draw random numbers from binomial distributions. This would not only be faster, it is also not an approximation, but exact within the stochasticity of the process.

- In my first report, I introduced a toy model to suggest that the stochasticity during the process to select compartments for merging and selection could be affecting the stability of the model.
    - I am now partly convinced that this is not the case, given that the Authors have provided many more replicas of each numerical experiment.
    - However, I find it very odd that the Authors first dismiss this possibility, and then use that precise same argument to explain why so many of their experiments in subsection “Computer simulations of transition of networks” end up extinct. Honestly, I am left with the feeling that the Authors do not have a great grip of their own work.

- In Fig. 7:
    - Could you please clarify what the gray trajectories are? Are we actually observing a transition HPP $\rightarrow$ HPH, instead of HP $\rightarrow$ HPH?
