# Supplementary material for: Plausible pathway for a host-parasite molecular replication network to increase its complexity through Darwinian evolution
Source: PLoS Comput Biol. 2022 Dec 1;18(12):e1010709. doi: 10.1371/journal.pcbi.1010709 (PMC9714742; doi:10.1371/journal.pcbi.1010709)
Supplement: Attachment — Submitted filename: 220708_pont-by-point_clean.docx [file pcbi.1010709.s020.docx]

Point-by-point responses

# Editor’s comment:

As with all papers reviewed by the journal, your manuscript was reviewed by members of the editorial board and by several independent reviewers. In light of the reviews (below this email), we would like to invite the resubmission of a significantly-revised version that takes into account the reviewers' comments. Following the comments by Reviewer 1, please make sure that a revised version of the manuscript accurately positions your work with respect to Takeuchi and Hogeweg (2008).

**Response:**

We are thankful to you and the reviewer for investing time and effort in the diligent reading of the manuscript and providing insightful comments, which have improved our manuscript significantly. Following the reviewers’ comments, we have thoroughly rewritten the manuscript. The revised version includes appropriate citation of the previous study by Takeuchi and Hogeweg, as suggested by reviewer 1. We believe that the revised manuscript addresses all concerns of the reviewers.

# Reviewer 1’s comments

**Major comments:**

**Comment 1-1:**

I have concerns about the academic fairness and scientific novelty of the manuscript. I start with academic fairness. In the abstract, the manuscript states, “The analysis shows that the most plausible complexification pathway from a single host replicator is the addition of a parasitic replicator, followed by the addition of a new host replicator that is resistant to the parasite.” This pathway of complexification is identical to that demonstrated in Takeuchi and Hogeweg (2008), which is quoted below for convenience (in the quote, “catalysts” refer to host replicators):

‘Thus, the presence of catalysts entails a “niche” (ecological functionality) for parasites. In the current system, the C-catalyst creates such a niche, and this enables the evolution of the G-parasite. Moreover, once the C-catalyst-G-parasite organization is established, it creates yet another niche, i.e., a niche for a phenotype that can escape from the G-parasite. Acquiring such a phenotype, the A-catalyst evolves. Finally, the establishment of such an alternative catalyst, in turn, creates a niche for a phenotype that can parasitize this alternative catalyst. This can cause the evolution of the U-parasite. ... In summary, the results of this section demonstrated a chain reaction of niche generation and speciation in an emergent ecosystem.’ However, the manuscript does not acknowledge that Takeuchi and Hogeweg (2008) describes the same pathway for the evolution of complexity as reported in this study. Instead, the manuscript states, “Furthermore, recent theoretical studies conducted by Takeuchi and Hogeweg showed that parasitic replicators induced diversification of RNA-like replicators through evolutionary arms race in compartmentalized structures and allowed the formation of more complex inter-dependent molecular networks” (Line 74-77). The authors’ description of Takeuchi and Hogeweg (2008) makes it impossible for the reader to see that the pathway demonstrated by the authors’ model is the same as that demonstrated by the model of Takeuchi and Hogeweg (2008). I think this equivalence needs to be explicitly stated in the manuscript (as well as the difference, as I mention at the end of my major comments).

**Response 1-1:**

First, we are thankful for your time and effort in reading and commenting on our manuscript, which has significantly improved it. After reading your comments, we noticed that our previous manuscript lacks a sufficient description of Takeuchi and Hogeweg’s previous study (2008). We recognize that the previous study addressed the same issue, and, hence, we have included appropriate citations of the study. We agree that we should have clearly described that the previous study by Takeuchi and Hogeweg as it was the first to address the possible complexification process of primitive replicators with parasitic entities. Accordingly, we have rewritten the Abstract, Introduction, and Discussion sections thoroughly, as shown below (see responses 1-4).

**Comment 1-2:**

In addition, Takeuchi and Hogeweg (2008) is published more than ten years ago. I do not think such a study is usually considered “recent”, as the authors write in the manuscript.

**Response 1-2:**

We have made the necessary corrections in the revised manuscript (see responses 1-4).

**Comment 1-3:**

Another related issue is in how the manuscript formulates the central question of this study. The manuscript states, “These experimental results support the idea that coevolution between host and parasitic replicators can drive diversification and complexification. However, it is still unknown how such complexification is possible and competitive exclusion among RNA species is circumvented” (Line 96-98). This question has been tackled by Takeuchi and Hogeweg (2008), which theoretically demonstrates a pathway for the evolution of complexity in an RNA replicator system and the importance of parasites and population structure for the evolutionary stability of ecological diversity. However, the manuscript introduces the above question as if no one has attempted to address it, which is wrong.

**Response 1-3:**

We admit that the description in the previous manuscript was inappropriate. We have corrected the parts in the revised manuscript (see Responses 1-4).

**Comment 1-4:**

The authors might have written the above things because they had inadvertently missed the points of Takeuchi and Hogeweg (2008). However, just in case that is not the case, I would like to add the following. If one covers up previous studies to glorify one’s own studies, it will damage one’s academic credibility in the long term, even if one might get away with the short-term benefit of more publications.

**Response 1-4:**

We are thankful for your comment. Of course, we had no intention to disregard the previous study, and we now understand that the previous description method was inappropriate. Following the reviewer’s comments, we have rewritten the Abstract, the second and fourth paragraphs of the Introduction, and part of the Discussion section as follows (revised parts are underlined).

**Abstract**

“How the complexity of primitive self-replication molecules develops through Darwinian evolution remains a mystery with regards to the origin of life. Theoretical studies have proposed that coevolution with parasitic replicators increases network complexity by inducing inter-dependent replication. Particularly, Takeuchi and Hogeweg proposed a complexification process of replicator networks by successive addition of a parasitic replicator followed by the addition of a new host replicator that is resistant to the parasitic replicator. However, the feasibility of such complexification with biologically relevant molecules is still unknown owing to the lack of an experimental model. Here, we investigated the plausible complexification pathway of host-parasite replicators using both an experimental host-parasite RNA replication system and a theoretical model based on the experimental system. We first analyzed the parameter space that allows for sustainable replication in various replication networks ranging from a single molecule to three-member networks using computer simulation. The analysis shows that the most plausible complexification pathway from a single host replicator is the addition of a parasitic replicator, followed by the addition of a new host replicator that is resistant to the parasite, consistent with the previous study by Takeuchi and Hogeweg. We also provide evidence that the pathway actually occurred in our previous evolutionary experiment. These results provide experimental evidence that a single replicator spontaneously develops into multi-replicator networks through coevolution with parasitic replicators, which might be the first complexification step toward to the emergence of life.”

**Introduction**

**Second paragraph:**

“Complexity is an ambiguous concept, and there are several measures for determining the complexity of a replication system, such as the amount of information encoded in a replicator (12), the number of traits of a replicator (13), the difficulty in achieving traits (14), the potential to cope with environmental uncertainty (15) and the number of replicators organized as a replication network (16,17). Here, we focus on one of the measures, the number of replicators in a replication network (i.e., network complexity). One of the possible pathways for a replicator to develop this complexity is the diversification of replicators and formation of inter-dependent, cooperative replication networks among them, such as a hypercycle (2,18–22). A major hurdle for inter-dependent network formation is parasitic replicators, which destroy the cooperative replication network (18,23). To date, theoretical (24–28) and experimental (29,30) studies have revealed that spatial structures such as compartmentalization repress parasitic replicators. Furthermore, Takeuchi and Hogeweg theoretically demonstrated that parasitic replicators induced diversification of RNA-like replicators through an evolutionary arms race in compartmentalized structures and allowed the formation of more complex inter-dependent molecular networks (16). In their simulation, an RNA species developed ecological complexity by successive addition of a parasitic RNA, followed by the addition of an RNA that is resistant to the parasitic RNA into the replicator network. The study suggests that parasitic replicators induced diversification of RNA-like replicators through evolutionary arms race in compartmentalized structures and allowed the formation of more complex inter-dependent molecular networks(16). In their simulation, an RNA species develops ecological complexity by successive addition of a parasitic RNA, followed by the addition of an RNA that is resistant to the parasitic RNA into the replicator network. The study suggests that parasitic replicators, which have been considered as an obstacle to complexification, may play an important role in complexification in compartmentalized structures. One of the remaining challenges is the plausibility of such complexification within a realistic parameter space that is achievable with biologically relevant molecules such as RNA and proteins. ”

**Fourth paragraph:**

“According to the previous theoretical study by Takeuchi and Hogeweg (16), such complexification is explained by the successive appearance of a parasitic species, which function as a niche for a new host species. This study examined whether our experiment can support the complexification process. To this end, we first constructed a theoretical model of a compartmentalized host-parasite replication system, which is conceptually the same as that developed by Takeuchi and Hogeweg but partially generalized for application in our experimental method and parameters. We then investigated the parameter space that allows for the sustainable replication of all replicators in each host-parasite replication network in up to three-member networks using computer simulation. We also conducted an evolutionary simulation by introducing new replicators with different parameters. These simulations were consistent with those of the previous study showing that the most plausible complexification pathway of a single host involves the successive addition of a parasite first and then a new host that is resistant to the parasite. Furthermore, we confirmed that the most plausible pathway (the addition of a parasite, followed by the addition of a parasite-resistant host) occurred in our previous evolutionary experiment.”

**The first and second paragraphs in the Discussion**

“In this study, we investigated the plausible complexification pathway of host-parasite replication networks using computer simulation and experiments. First, we examined the parameter space that allowed sustainable replication of all members in replication networks from two- (HH and HP) to three-member networks (HHH, HHP, and HPP). Sustainable parameter spaces are broader in HP and HHP networks for the range of the parameters we used, suggesting the plausibility of complexification from a single replicator to HP and then to HHP networks. We further confirmed that the dominant RNAs isolated from the previous evolutionary experiments had parameter sets that sustained HP and HHP networks, suggesting that the transition of replication networks actually occurred during the evolutionary experiment, consistent with the previously proposed scenario by Takeuchi and Hogeweg (16). These results provide both theoretical and experimental evidence that support the validity of the previously proposed complexification scenario by Takeuchi and Hogeweg (16) and also provide evidence that the spontaneous development of a complex reaction network through Darwinian evolution is feasible within the parameter space that is achievable with RNA and proteins and that coevolution with parasitic replicators plays an important role in the complexification.

To date, the conditions required for the coexistence of multiple replicators have been studied using various theoretical models (15,34–37). The sustainable conditions observed in this study are consistent with those of previous studies. For example, the sustainability of an HHP network that requires a parasite and asymmetric parasite resistance between the two hosts (Fig. 5C) is consistent with the idea that the parasites play a role as a “niche” to sustain different types of host species (15,36,37). In addition, the sustainability of HH networks that require larger cross-replication than self-replication (Fig. 3C) is not a new concept because it is fundamentally the same as the cooperative relationship found in hypercyclic networks (17), in which an information carrier cannot replicate by itself. This consistency with previous theoretical studies, however, does not diminish the importance of this study because the novelty of this study is not to provide a new concept for the coexistence theory but to provide experimental evidence that such pathways and parameters can be realized by the action of biologically relevant molecules, such as RNA and proteins. In this study, we found that the RNAs and the encoded replicase protein were able to have replication parameters that permit sustainable HP and HHP networks under compartmentalized conditions. We also found that experimentally obtained RNAs are on the edge of the sustainable parameter space (shown in the magenta square in Fig. 5D), which implies that a slight change in one of the parameters easily destroys the sustainability. The analysis of this study using an experimental model and relevant computer simulation revealed the realistic yet fragile nature of molecular replication networks.”

**Comment 1-5:**

The other major issue I have is that I do not see the scientific novelty of the modelling results as stated in the manuscript. I am not talking about the modelling results per se, but talking about the novelty as stated in the manuscript. In Lines 405-409, the manuscript states, “the novelty of this study is not to provide a new concept for the coexistence theory but to reveal realistic pathways and parameters for the complexification in biochemical replicator systems.” First, this quote indicates that the authors themselves do not think this study provides a new theoretical concept, which is in agreement with what I wrote above in relation to Takeuchi and Hogeweg (2008).

The second part of the above quote says that this study reveals more realistic pathways and parameters than known before. However, I do not see how this is achieved in this study. The authors’ model mimics a synthetic evolving system engineered by the authors. Mimicking a particular experimental system certainly makes it easier to test modelling results experimentally. However, in my opinion, that by itself does not necessarily make modelling results more realistic or relevant to the question of the origins of life than the other models because the level of abstraction employed in the authors’ model is similar to that of many other models, and there is not enough evidence indicating that the particular experimental system mimicks prebiotic reality.

**Response 1-5:**

The sentence, “the novelty of this study is … to reveal realistic pathways and parameters for the complexification in biochemical replicator systems,” in the previous manuscript might have been unclear. In this sentence, we did not intend to state that the pathway and parameters depicted in our study were realistic or relevant to the question of the origin of life. We aimed to state that the pathway and parameters were realistic in the sense that they could be realized with a biochemical replicator system composed of biologically relevant molecules, RNA, and proteins. We believe that the words, “realistic” and “biochemical replicator systems” in the sentence were unclear. We have rewritten the sentence in the revised manuscript as follows (the revised parts are underlined):

“This consistency with previous theoretical studies, however, does not diminish the importance of this study because the novelty of this study is not to provide a new concept for the coexistence theory but to provide experimental evidence that the pathways and parameters can be realized by the action of biologically relevant molecules, such as RNA and proteins.”

**Comment 1-6:**

The manuscript also states that a subset of the model parameters is based on experimental measurements. However, that does not necessarily make the model more realistic than the others either, for the following reasons. First, in Lines 165-169, the manuscript states that one of the two conditions in which two host replicators can coexist is that rate constants are balanced (k_11=k_12, k_21=k_22). The values of k_11, k_21 etc., were taken from experimentally measured values. However, it is evidently unrealistic that each pair of rate constants is exactly identical to one another, even if particular values of rate constants were obtained through experiments. There will be errors in the measurements too.

**Response 1-6**:

We used the word “realistic” in the sense that the parameter values were achievable with biologically relevant molecules, RNA, and proteins. We believe that this is an advantage of our model compared with other purely theoretical models. However, we agree that the two RNA species with identical parameters are unrealistic. To clarify the meaning of “realistic” in this context, we have added the following sentence in the corresponding result section.

“Notably, the “balanced replication” condition, which requires identical parameters, is unlikely with realistic molecules.”

**Comment 1-7:**

Another reason why using experimentally measured parameters does not necessarily make the model more realistic is that the model still uses multiple parameters that have not been experimentally measured, e.g., mutation rates and the ratio of mutating into parasites or hosts. Consequently, the results of the model do not necessarily match those of the experiments. For example, the manuscript states, “Starting from a single host species (k 11 = 2.0), we performed 3000 rounds of serial replication cycles for 100 times and found that in most of the runs (97 runs), all replicators were diluted out, while in three runs, the HHP network was formed.” Did the experiments also result in the evolution of the surviving network only in three out of 100 replicates? If so, is this fact explicitly reported, e.g., in Bansho et al. 2016 or Furubayashi et al. 2020?

**Response 1-7:**

We admit that the mutation rate and parasite appearance rate used in the previous evolutionary simulation were not realistic. We revised the values to more realistic values and conducted a new evolutionary simulation, which has been presented in the revised manuscript. In this case, the disappearance rate was still high (796 out of 1000 simulations). This high disappearance (i.e., low survival) rate is seemingly inconsistent with our experiment, in which we never observed the extinction of RNA species for more than 240 rounds of continuous replication experiments. We believe that this discrepancy can be attributed to the much smaller number of compartments (3000) in the simulation than in the experiment (10^8^). This hypothesis was supported by our new evolutionary simulation performed with smaller and larger number of compartments (1000 and 5000, respectively), in which the disappearance rate increased (936 disappearance in 1000 simulations) or decreased (470 disappearance in 1000 simulations), respectively. These new simulations and explanations have been added to the revised manuscript as follows.

**“****Computer simulation of transition of networks**

Next, we investigated the possible evolutionary transitions of networks using computer simulations. We introduced a mutation step into the serial replication cycle immediately before the replication step. During the mutation step, a new host or parasite appeared at a certain rate in one of the compartments if the total number of replicator species in the system was less than three. A new host was generated from existing hosts at a rate of 0.02 per replication, and a new parasite was generated from hosts and parasites at rates of 0.001 and 0.002 per replication, respectively. These generation rates were partially based on experimental data (see Methods for details). A new host or parasite had replication coefficients randomly chosen from 1 to 3 or 0 to 10 as a type of double-precision floating-point number, respectively.

Starting with a single host species ($k_{11}^{H}$ = 2.0), we performed 1000 rounds of serial replication cycles 1000 times. We counted the number of networks that were maintained for more than 100 rounds during the replication cycles and found that HPP, HHP, and HHH networks were maintained in 19, 218, and 14 runs, respectively (Fig. 7A). This finding indicated that the HHP network was easier to maintain than other three-member networks, which was consistent with our sustainability analysis (Figs. 4-6). To investigate the network from which the HHP network was formed, we examined the network immediately preceding the 218 HHP networks. Most (153) of the HHP network was preceded by HP networks (Fig. 7A), supporting the hypothesis that the transition from HP to HHP network is the most plausible route. Fig 7B shows a typical example of the population dynamics in the simulation that reached the HHP network. An HP network was formed around round 520, and then it became an HHP network around round 560.

Next, we investigated the coefficients of 218 HHP networks maintained in the evolutionary simulation. According to the sustainability analysis shown in Fig. 5, the HHP network was sustainable when two hosts showed asymmetric resistance to the parasite, and non-resistant hosts replicated more than resistant hosts (i.e., $k_{11}^{P}$ > $k_{21}^{P}$ and $k_{11}^{H}$ + $k_{21}^{H}$> $k_{12}^{H}$ + $k_{22}^{H}$). We found that 170 out of 218 HHP networks satisfied this condition. This consistency between evolutionary-determined and stable parameter values has been suggested previously to support the reliability of sustainable conditions (35).

During 1000 rounds of replication cycles, all replicators disappeared in 796 of 1000 simulations. This high rate of disappearance was seemingly contradictory to the experimental results, in which we have never experienced disappearance of all RNA species (32). This contradiction can be explained by the differences in the number of compartments. In this evolutionary simulation, we used 3000 compartments, whereas the experiment had approximately 10^8^ compartments. If the number of compartments decreases, the chance of accidental extinction of RNA species should increase. To verify the effect of the compartment number, we performed the same evolutionary simulation with smaller (1000) and larger (5000) number of compartments, in which the disappearance rate increased (936 disappearance in 1000 simulations) or decreased (470 disappearance in 1000 simulations), respectively. This finding supports that the larger disappearance rate in the simulation can be explained by the smaller number of compartments.”

**Figure 7. Computer simulation of the evolutionary transition of replication networks.**

The evolutionary transition was simulated by introducing a mutagenesis step in the serial replication cycle, as shown in Fig. 2, for 1000 rounds. A) The number of networks maintained for more than 100 rounds during replication cycles in 1000 simulations was counted. B) A typical trajectory of the concentrations in one of the simulations that resulted in the formation of the HHP network.

**Methods**

**Evolutionary simulation**

A mutation step was introduced immediately before the replication step to simulate the evolutionary transition shown in Fig. 7. In this step, a new host is generated from existing hosts at a rate of 0.02 per replication, based on the mutation rate of Qβ replicase (36) and the typical RNA size (2000 nt). A new parasite is generated from a parasite at a rate of 0.002 per replication, because of approximately 1/10-fold smaller RNA size. Since we could not find reliable data for the generation rate of a new parasite from a host, we used the value of 0.001, which was smaller than the generation rate of a new parasite from the parasite. To reduce the computational cost, the total number of replicator species was restricted to less than three. In other words, a new species could appear in the mutation step only when the total number of replicator species is one or two. For computational ease, only one type of new replicator can appear in one round for both the host and parasites (i.e., the simultaneous appearance of two types of hosts was not allowed, but the simultaneous appearance of one type of host and one type of parasite was allowed).

When the total number of replicator species was less than three, the mutation process was conducted as follows. First, the number of replicates at the replication step was counted for each replicator in each compartment. Second, the probability of the appearance of a new replicator was calculated by multiplying the replication number with the mutation rate. If the probability defined for each replicator in each compartment was less than a random value $\in[0, 1]$, then a new host or parasite appeared in the compartment. The probability was typically very low, and thus, only one new host or parasite appeared in each round in most cases. However, for simplicity, when two new hosts or two new parasites simultaneously appeared, we assumed that the two new species were the same (i.e., had the same coefficients). Third, the coefficients of a new host (a new host *i* replicated by host *j* ($k_{ji}^{H}$)) were randomly chosen from 1 to 3 as a type of double-precision floating-point number. The coefficients of a new parasite *h* replicated by host *j* ($k_{jh}^{P}$) were randomly chosen from 0 to 10 as a double-precision floating-point number. The simulation was started from a single host that self-replicated with a coefficient of 2.0 and continued for 1000 rounds of serial replication cycles. The number of compartments ($C$) was 3,000, the selection rate ($S$) was 0.25, the fusion-division frequency ($A$) was 5,000, and the carrying capacity ($N$) was 100. The code for the simulation has been uploaded to GitHub (https://github.com/Dokunuma/PrebioDivSim).

**Comment 1-8:**

To increase the plausibility of the modelling results, I think it is better to test a wide range of parameters rather than using a selected few or to let parameters be determined by evolution as suggested by Hogeweg (https://doi.org/10.1007/978-3-030-71737-7_2). In fact, the authors do test parameter values that appear to be selected without experimental measurements according to the manuscript. In my opinion, what makes this type of modelling results more realistic is evolutionary stability rather than setting a subset of parameters to experimentally measured values.

**Response 1-8:**

Thank you for pointing this out to us. The unsupervised evolutionary approach for finding reliable parameter values in the suggested paper is very useful and is applicable to our evolutionary simulation. If the parameter values reached after the evolutionary simulation match the coexistence conditions, the plausibility of the modeling results would be supported. To test this, we performed evolutionary simulations with more continuous parameter values (instead of discrete few values, as described in the previous manuscript) in Fig. 7 and found that the finally obtained parameters in the surviving HHP network satisfied the coexistence conditions, such as asymmetric parasite replication and a higher replication rate of the parasite-susceptible host than that of the parasite-resistant host. This result has been described in the section “Computer simulation of the transition of the networks” in the result section as shown in Responses 1-7 (see the paragraph that starts from “Next, we investigated the coefficients…”).

**Comment 1-9:**

There is another reason that makes me think that the modelling results described in the manuscript are not novel. In Line 163, the manuscript reports one of the two conditions required for two host replicators to coexist (HH network), namely that two hosts replicate cooperatively. That cooperation is necessarily has been known since the 70s through the hypercycle theory of Eigen and Schuster. In Line 405, the manuscript states that the HH network is “similar” to the hypercycle. What is the difference? Given that the hypercycle can survive in a well-mixed system in the absence of parasites, one can easily expect that it can also survive in a loose-compartment system because the main effect of loose compartmentation is to make a system more resistant to parasites, but the system lacks parasites. So, in my opinion, there is hardly any novelty in this part of the authors’ results.

**Response 1-9:**

As per our understanding, the HH network is slightly different from a hypercycle in that each host can replicate by itself without the other host, whereas the replication of replicators (information carriers) in a hypercycle largely depends on another replicator. However, we do not intend to insist on the novelty of this small difference. We agree that our results on the HH network are not new. We have included the HH network results in the manuscript for the completeness of all less-than-three-member networks. To avoid confusion, this point (i.e., the coexistence of the HH network is not new) has been explicitly described in the Discussion section of the revised manuscript as follows:

“In addition, the sustainability of HH networks that require larger cross-replication than self-replication (Fig. 3C) is not a new concept because it is fundamentally the same as the cooperative relationship found in hypercyclic networks (17).”

**Comment 1-10:**

In my opinion, the novel results of this study are two folds. First, the authors’ model shows that the pathway of complexification proposed by Takeuchi and Hogweg (2008) is valid in a loosely-compartmentalised system without spatial self-organisation. Second, the authors’ experiments lend support to their modelling results. So, in my opinion, the manuscript is actually answering a different question from that mentioned above. The manuscript states, “One of the remaining challenges is the plausibility of such complexification within a realistic parameter space that is achievable with biologically relevant molecules such as RNA and proteins.” The manuscript tackles this challenge by theoretically generalising and experimentally supporting the pathway of complexification predicted in Takeuchi and Hogeweg (2008).

**Response 1-10:**

Thank you for highlighting the novelty of our study. We agree with your suggestion. As mentioned in the aforementioned responses, we have thoroughly rewritten the manuscript (Abstract, Introduction, and Discussion sections) according to your suggestion, as described above.

**Minor comments:**

**Comment 1-11:**

It is not explained how the equation in Line 541 comes about. Please describe the mathematical justification.

**Response 1-11:**

Based on the comment, we have added the following explanation as supplemental text.

Additionally, we have added a superscript for each coefficient *k* to clarify the RNA (host or parasite) it belonged to.

“**Derivation of eq. 3**

As described in the methods section, the differential equation for host replication is as follows:

$$\frac{dH_{i}}{dt}=H_{i}\left( \sum_{j} k_{ji}^{H}H_{j} \right)\left( 1-\frac{\sum H_{j}+\sum P_{j}}{N} \right)$$

For the two-step experiment conducted in Fig. 9A, replication was stopped before the carrying capacity was reached; thus, the effect of the carrying capacity, $\left( 1-\frac{\sum H_{j}+\sum P_{j}}{N} \right)$, was neglected. Then, we obtained $\frac{dH_{i}}{dt}=H_{i}\left( \sum_{j} k_{ji}^{H}H_{j} \right)$*.* In this equation, the first factor, $H_{i}$, represents the template RNA to be replicated, and the second factor, $\left( \sum_{j} k_{ji}^{H}H_{j} \right)$, represents the total replication enzymes provided by all host RNAs. To estimate $k_{12}^{H}$ and $k_{21}^{H}$, the coefficients of Host^1^_exp_ and Host^2^_exp_, we conducted a two-step reaction, as shown in Fig. 9, in which the replication enzymes were produced from only the first RNA (RNA I), and both RNA I and II were replicated using replicase. For example, for a reaction with host *i* as RNA I and host *j* as RNA II, the rate of host replication in the second reaction should be $\frac{dH_{i}}{dt}=H_{i}\left( k_{ii}^{H}\bar{H_{i}} \right)$ and $\frac{dH_{j}}{dt}=H_{j}\left( k_{ij}^{H}\bar{H_{i}} \right)$, where $\bar{H_{i}}$ represents host *i* concentrations in the first reaction, and thus they are constant values. Solving these equations gives $H_{i}=H_{i}^{t=0}e^{k_{ii}^{H}\bar{H_{i}}}$ and $H_{j}=H_{j}^{t=0}e^{k_{ij}^{H}\bar{H_{i}}}$, where $H_{i}^{t=0}$ and $H_{j}^{t=0}$ represent the concentrations of RNAs *i* and *j*, respectively, before the second reaction. In the two-step replication experiment, we measured the increase in the ratios from 0 to 1 h in the second reaction as fold values (Fig. 9B). We defined the common logarithms of the fold value as $v_{ij}^{h}$, where the subscripts *i* and *j* represent the RNA species used as RNA I and II, respectively. Accordingly, the above equations yield ${v_{ij}^{i}=k}_{ii}^{H}\bar{H_{i}}\log e$ and ${v_{ij}^{j}=k}_{ij}^{H}\bar{H_{i}}\log e$. Therefore, we obtained $k_{ij}^{H}= k_{ii}^{H}\frac{v_{ij}^{j}}{v_{ij}^{i}}$. Similarly, $k_{ij}^{P}= k_{ii}^{H}\frac{v_{ij}^{j}}{v_{ij}^{i}}$ is obtained when the replicator *j* is a parasite.”

**Comment 1-12:**

The replication rate was measured under experimental conditions that were different from those in which evolutionary experiments were done. Specifically, a solution contained only one or two genotypes in the measurements, whereas a solution contained multiple genotypes in evolutionary experiments. Could you discuss whether this could potentially make any difference? For example, the activity of enzymes could be modulated by the presence of other RNA molecules? Could there be RNA-RNA interactions?

**Response 1-12:**

There could be many differences between experiments performed using clonal RNAs and RNA populations during evolution. However, to date, we have not detected any higher-order interactions between RNAs that cannot be explained by experiments performed using clonal RNAs. The note about this point has been newly included in the corresponding results section of the revised manuscript as follows:

“Note that we measured replication coefficients with clonal RNAs in this experiment, whereas during the evolutionary experiment, the RNAs replicated in a population comprised various RNA species. In a population, RNAs may be replicated with higher-order interactions among various RNAs. However, we analyzed the contributions of such higher-order interactions in our previous study and found that the contributions were minor, at least until round 240 (Supplementary Text 2, Figs. S17 and S18 in Mizuuchi et al)(32).”

**Comment 1-13:**

In Line 503-505, the manuscript says, “The coefficients of a new host are randomly chosen from 1.7, 1.8, 1.9, 2.0, 2.1, 2.2, 2.3, 2.4, 2.5, and 2.6. Coefficients of a new parasite were randomly chosen from 0.01, 0.1, 0.5, 1, 2, 3, 4, 5, 6, 7, 8, 9, and 10.” I did not understand exactly what the authors did. Do the coefficients refer to k_ji values? If so, parasites do not replicate anything, so they should not have positive coefficients. Moreover, there are nine distinct k_ji values if the system contains three genotypes (i and j can each take three values, so three times three). How is each of the k_ji values selected from a set of values listed above?

**Response 1-13:**

We believe that the previous use of symbols was confusing. To avoid confusion, we have used more specific symbols for coefficients in the revised manuscript. The coefficient of parasite *j* replication by host *i* is represented by $k_{ij}^{P}$. The coefficient of host j cross-replication with host *i* is represented by $k_{ij}^{H}$. The coefficient of host *i* ’s self-replication is represented by $k_{ii}^{H}$. In addition, the range of parameters for a new host or parasite has also been revised.

Accordingly, $k_{ij}^{P}$ was set between 0 and 10. $k_{ij}^{H}$ and $k_{ii}^{H}$ were selected from 1 to 3. We have used these symbols in the revised manuscript.

**Comment 1-14:**

The equations in Lines 459-460 assume continuous variables. However, I believe that the system is unstable without any stochasticity in the distribution of molecules over compartments. Something is missing in the description of the model, probably around Lines 479-481.

**Response 1-14:**

As the reviewer commented, stochastic distribution of RNAs over the compartment is needed for stable replication. Stochastic distribution is achieved in the fusion-division step, in which two compartments are randomly selected and the internal RNAs were summed up (i.e., compartments were fused). Then, we divided the fused compartment into two compartments and randomly distributed each RNA into two new compartments. If the number of RNA is small, the stochasticity of the RNA distribution increases through this step. To clarify this point, along with other points, we have rewritten the paragraph in the Results section of the revised manuscript as follows (the revised parts are underlined).

“The fusion-division phase (Fig. 2D) mimicked the experimental process to mix the contents of compartments through repeated fusion and division process of the compartment (31). In this simulation process, the following three steps were repeated $A$ times ($A$ is defined as “fusion-division frequency”). First, two compartments were randomly chosen from all compartments. Second, the concentrations of each replicator (i.e., host or parasite) in the two compartments were summed to mimic the compartment fusion. Because the amount of each replicator in a compartment was defined as concentration, not a number, at this stage, we approximated the number of each replicator by rounding off the concentrations. Third, the replicators in the fused compartment were randomly redistributed into two new compartments to mimic the division of the compartment. In this step, we flipped a coin for each replicator, and thus, stochasticity appeared in RNA composition especially when the number of RNAs was small.”

**Comment 1-15:**

In Lines 420-423, the manuscript appears to suggest that the HH network (hypercycle) cannot survive in the presence of parasites in a loose-compartment system. However, as shown by Boerijst and Hogeweg (1991 Physica D), population structure can enable a hypercycle to survive in the presence of parasites. I feel that this result can be extended to a loose-compartment system because the authors have experimentally shown that two cooperating hosts can survive in a loosely-compartmentalised system even if mutations can generate parasites (Mizuuchi et al., 2022 Nat Comm). Thus, if the authors’ model and experiments are consistent with each other, the model is expected to display the survival of a hypercycle in the presence of mutations into parasites. If not, there is something more to learn about.

**Response 1-15:**

We believe that our unclear description has been misunderstood by the reviewer. In Lines 420-423 of the previous manuscript, we did not intend to state that the HH network (hypercycle) cannot survive in the presence of parasites. We aimed to state that the HH network might be unlikely because it soon changes to the HHP network once the parasite appears. To avoid this misunderstanding, we have rewritten the corresponding part (Line 494) as follows in the revised manuscript:

“However, such replication networks might not last long because parasitic replicators would appear soon.”

**Comment 1-16:**

**Minor errors:**

Line 232: “allows” should be “allow”.

Line 424: (39) should be (38).

Line 489: I do not understand this sentence, “all compartments contain all hosts or parasites on average”.

**Response 1-16:**

Thank you for pointing this out to us. We have corrected the first and second errors and have erased the sentence listed in the third point of the revised manuscript.

# Reviewer 2’s comments

**Comment 2-1:**

I have now read and assessed the manuscript titled “Plausible pathway for a host-parasite molecular replication network to increase its complexity through Darwinian evolution”. I think that this paper contains very interesting science, and I hope that some version of it is published in a very good journal. To do so, I think that the Authors need to tackle some major issues that affect the work.

These issues range from the methodological to the interpretation of the results and experiments. I think that some issues must necessarily be tackled in order to attain a version of the manuscript that can be published. On the other hand, some conflicting points seem to arise from a different understanding of the dynamics that happen during the experiments. The Authors seem to write from a “population dynamics” perspective, which is grounded on currently accepted views of Darwinian evolution. It is my opinion that such views are incomplete and, crucially, that the “population dynamics”-limit is reached under conditions that, I think, are not met in this experiment. As I will explain, this causes problems in the interpretation of the experiments.

I acknowledge that these later issues are a matter of opinion and interpretation. It would not be fair for me to discredit, on such basis, the great scientific work done by the Authors. Furthermore, I would expect that most reviewers with a strong biological background would accept the “population dynamics” account by the Authors without hesitation. However, I think that the conflict between interpretations raises some important questions, and that the paper would be greatly improved if these issues were tackled or at least discussed.

Response 2-1:

We are thankful for your time and effort invested in reading and commenting on our manuscript, which has significantly improved it. We are also grateful for your thoughtful comments. We have addressed all methodological issues in the revised manuscript, as described below. Regarding the interpretation issue (i.e., “population dynamics” or “quasispecies dynamics”), we agree that interpretation from a quasispecies viewpoint is necessary for understanding some (but not all) parts of our results. Hence, we have added several sentences in the Results section and a new paragraph that interpreted our results from a quasispecies viewpoint in the Discussion section (see the following responses for details).

**Comment 2-2:**

In this manuscript the Authors combine computational and experimental methods to asses how a small ecosystem might increase its complexity over time. The ecosystems studied consist of hosts (which might cooperate or compete with each-other) and parasites (which benefit from hosts but do not help them in return). The ecosystem dynamics are encoded by logistic equations whose coefficients determine how intense competition and cooperation are. The Authors study numerically the stability of equations for several values of the coefficients, thus finding combinations of parameters for which host-host, host-parasite, host-host-host, and host-parasite-host configurations

are stable. They find that adding a parasite increases the ranges of parameters leading to stable coexistence, and thus determine that the most likely route for the ecosystem to gain complexity is, starting from a single host, to then acquire a parasite, and then yet another host. The Authors speculate that this mechanism could be iterated for larger ecosystems to gain parasitic and host species alternately. In a final, important part of the paper, the Authors compare this route to complexification with results from earlier experiments that they had performed. They conclude that the proposed route was observed in their previous data, and perform new experiments to argue that the final configuration (allegedly, with two hosts and a parasite) is stable.

As pointed out above, several issues exist that affect critical phases of the chain of reasoning presented by the Authors.

On the one hand, the methodology for the numerical experiments is not completely clear, and, in my opinion, leaves the door open to the possibility that the Authors are not assessing the stability of the system correctly. This requires a lot of clarification. Methodological problems also affect the only one simulation presented to show the route to complexification (which I will note HPH for short) in action.

On the other hand, I have some serious issues with interpreting that HPH is what is actually going on in the experiments. These issues are mostly derived from my interpretation of Darwinian dynamics in these experiments. These issues make me doubt the interpretation that their configuration is stable, as the Authors attempt to argue with their new experiments.

In the following, I present bullet points with specific issues. These points are presented in a section-wise manner: Introduction, Methods, Results. Within each section, bullets appear in the same order as in the text.

**Response 2-2:**

Regarding methodological issues, we performed a series of additional simulations, and we have included the data in the revised manuscript. In addition, we have clarified the description that was unclear in the previous manuscript.

Regarding the interpretation of the experimental results, we agree that quasispecies are necessary to explain some of the results. We have rewritten the corresponding parts of the revised manuscript accordingly. See the following responses for further details:

**Introduction**:

**Comment 2-3:**

• Abstract (lines 42-45) and lines 59-61:

◦ I think that it is important to discuss what processes (including Darwinism) led to the complexification of prebiotic chemistry. However, in the way that the Authors frame this issue, it generates the expectation that the paper will make more explicit contributions towards clarifying this crucial step.

**Response 2-3:**

As per our understanding, the reviewer’s concern is the origin of a new RNA species that forms replicator networks. In our perspective, a new member originated from a distinct RNA species that accumulated multiple mutations through Darwinian evolution forming their own lineage. The reviewer’s view is that it could originate from a member of a quasispecies that does not necessarily experience Darwinian evolution. After reading all reviewers’ comments, we now consider that both scenarios are required to explain all experimental results. As described in detail in Responses 2-35, we think Host_1_^exp^ and Host_2_^exp^ at round 39 accumulated multiple mutations through Darwinian evolution forming their own lineages, consistent with our original perspective. However, the host and parasite RNA at round 39 had quasispecies, which represented many small branches in the phylogenic tree (Figs. 8A and 8B). To explain this result, the quasispecies perspective is required. Accordingly, we have added a new paragraph in the Discussion section to explain the possible contribution of quasispecies in our experiment (see Response 2-36 for detail).

**Comment 2-4:**

• In lines 96-98:

◦ Introducing for the first time our different interpretations of the dynamics: Note that competition among RNA species as described by population dynamics (which leads to competitive exclusion) is attained in a certain limit. Among the conditions to reach this limit is that species are well delimited. This is not the case in the presented experiments, in which broad coexistence between RNA-sequence variants is persistently observed—contradicting competitive exclusion. These apparent issues disappear if we adopt a quasispecies interpretation of the experiments.

**Response 2-4:**

We agree that the “competitive exclusion” perspective could not explain the coexistence of various RNA variants (i.e., many small branches) in Figs. 8A and 8B. Nevertheless, we think the “quasispecies” perspective alone is also insufficient to explain the existence of two genetically distant RNA variants, such as Host_1_^exp^ and Host_2_^exp^, which differ by seven mutations and are unlikely to be included in the same quasispecies, as explained in detail in Responses 2-35 and 2-36 below. Therefore, we have employed a combined perspective in the revised manuscript. See Response 2-36 for revised sections.

**Comment 2-5:**

• I think that the following papers might be important for this work:

◦ Seoane LF, Sole R. Information theory, predictability and the emergence of complex life. *R. Soc. Open Sci.*, **5**(2), p.172221 (2018).

◦ Seoane LF, Sole R. How Turing parasites expand the computational landscape of digital life. arXiv preprint arXiv:1910.14339.

◦ Hickinbotham SJ, Stepney S, Hogeweg P. Nothing in evolution makes sense except in the light of parasitism: evolution of complex replication strategies. *R. Soc. Open Sci.*, **8**(8), p.210441 (2021).

**Response 2-5:**

Thank you for suggesting these important references. We have cited these studies in the revised manuscript.

**Methods**:

**Comment 2-6:**

I have two main issues with the computational methods of this paper. On the one hand, I do not feel very satisfied with the number of replicas carried out for each combination of parameters. On the other hand, the description of the simulations is rather obscure. There are some parts that I could not understand. Actually, my attempts to reproduce some critical steps led me to dynamics that might artificially alter the stability that the Authors attempt to study.

Regarding the number of replicas: The model described is relatively simple, and it should be possible to run even thousands of instances for each combination of parameters within a very reasonable time. Furthermore, the Authors write that a huge amount of the simulations do not even make it pass the first round of Replication, Selection, and Fusion-Division. I think that the amount of replicas (10 or even 3 in some cases) is very low for a computational study. Below, I interleave some ideas that might help speed up the simulations.

**Response 2-6:**

We admit that the number of replicas in our original manuscript was small because of the heavy computational load. Most of the computational time was used to solve the differential equations for RNA replication in each compartment. We evaluated the reaction for each of the 3000 compartments in every round. This calculation was necessary because each compartment typically had a different RNA composition. Of course, we believe there is room for reducing the computational cost by approximating or omitting unnecessary calculations. However, by following some of your suggestions and using a more powerful computer, we were able to examine 100 replicas for all main simulations in the revised manuscript. The tendencies of the results were the same as those with 10 replicas. Although the number may still be smaller than other computational studies, we believe that it is sufficient to evaluate the sustainability of host-parasite replicator networks we would like to propose here. Accordingly, we have revised all simulation figures and the corresponding text in the revised manuscript.

**Comment 2-7:**

First, in this regard (and perhaps I am mistaken), might the Authors be using a very slow programming language? Languages such as Python or R, which are often used in implementing computational biological models, are often thousands of times slower than lower-level languages such as C++ or ForTran.

**Response 2-7:**

Following the reviewer’s advice, we have changed the language from Python, which was used in the previous simulation, to C++ in the new simulation. The codes have been uploaded to GitHub (https://github.com/Dokunuma/PrebioDivSim).

**Comment 2-8:**

A list of points that should be addressed follows:

• In lines 459-460:

◦ There are three sums in each equation. Two of these sums (those of hosts) run over the same sets, while the sum over parasites runs over a different set. Perhaps it would help to use different indexes for hosts and parasites.

◦ Similarly, two different sets of *k_ij* appear: One set links hosts to hosts and another one links hosts to parasites. It would make things easier for the reader if the difference between these two sets were explicitly marked by using different symbols. As the equations are written, we could assume that a same number of hosts and parasites exist, and that they have the same coefficients *k_ij*.

◦ A similar comment applies to the left-hand-side of these equations: By labeling both hosts and parasites with *i*, it seems that they belong in a same set—which is not the case.

**Response 2-8:**

Following your suggestions, we have changed the following equations to clarify the meaning of the variables.

$\frac{dH_{i}}{dt}=H_{i}\left( \sum_{j} k_{ji}^{H}H_{j} \right)\left( 1-\frac{\sum_{j} H_{j}+\sum_{h} P_{h}}{N} \right)$,	eq. 1

$\frac{dP_{h}}{dt}=P_{h}\left( \sum_{j} k_{jh}^{P}H_{j} \right)\left( 1-\frac{\sum_{j} H_{j}+\sum_{h} P_{h}}{N} \right)$,	eq. 2

**Comment 2-9:**

• In lines 463 and 469-470:

◦ “*N* is the carrying capacity of each compartment.” and “The sizes of all compartments are the same.”. I hence assume that *N* is the same for all compartments, but this is not explicitly stated and both sentences are far apart (the connection might get lost).

**Response 2-9:**

We have rewritten the sentence as “*N* is the carrying capacity and is the same for all compartments,” in the revised manuscript.

**Comment 2-10:**

• In lines 472-475:

◦ After *C* compartments have been simulated, only *C_S* of them are used. This means that *C-C_S* compartments have been simulated only to be discarded. Since the selection rate is *S=0.25*, this means that the Authors are discarding 3 out of every 4 simulations that they run. If, instead of discarding compartments after the replication phase, they would do it before, then the number of replicas of the simulations could be increased 4-fold with the same current setup (i.e., there would be up to 40 or 12 repetitions for each combination of parameters).

**Response 2-10:**

Thank you for your suggestion. We have performed the procedure suggested in the previous simulation.

**Comment 2-11:**

◦ It is stated that “empty compartments were supplied up to the fixed total number of compartments”. I assume that *C-C_S* empty compartments are supplied, but this is not clear from the text. In my first reading I assumed that *C* new empty compartments had been provided, and that they would be filled from combinations of earlier compartments. I am still not convinced that this second interpretation is not the right one—if it were, it would clarify some of the following points.

**Response 2-11:**

We apologize for the confusion. We supplied C $-$ C_S_ compartments. To clarify this point, we have revised the corresponding part as follows (the revised part is underlined):

“In the selection phase (Fig. 2C), a certain number ($C_{S}$) of compartments was randomly selected, and $C-C_{S}$ empty compartments were supplied to maintain a fixed total number ($C$).”

**Comment 2-12:**

• In lines 477-483:

◦ This paragraph is particularly obscure in the details. I doubt that the exact same experiment would be reproduced by a reader. Many details are missing. Also, depending on some of these details, it might be possible to increase the efficiency of the code (resulting in more replicas for each combination of parameters).

**Response 2-12:**

We apologize for this insufficient description. In the revised manuscript, we have described the procedure in more detail as follows. We have also uploaded the code to GitHub (https://github.com/Dokunuma/PrebioDivSim).

“The fusion-division phase (Fig. 2D) mimicked the experimental process to mix the contents of compartments through repeated fusion and division process of the compartment (31). In this simulation process, the following three steps were repeated $A$ times ($A$ is defined as “fusion-division frequency”). First, two compartments were randomly chosen from all compartments. Second, the concentrations of each replicator (i.e., host or parasite) in the two compartments were summed to mimic the compartment fusion. Because the amount of each replicator in a compartment was defined as concentration, not a number, at this stage, we approximated the number of each replicator by rounding off the concentrations. Third, the replicators in the fused compartment were randomly redistributed into two new compartments to mimic the division of the compartment. In this step, we flipped a coin for each replicator, and thus, stochasticity appeared in RNA composition especially when the number of RNAs was small.”

**Comment 2-13:**

▪ Assuming *C-C_S* empty compartments were provided and a selection rate of *S=0.25*, at the beginning of the fusion-division phase most of the compartments are empty. In my best understanding of this paragraph, two compartments are chosen randomly. It is most likely that these two compartments will be empty—thus the Authors are combining empty compartments very often, which is inefficient. Back to the pipeline, the two selected compartments are combined and then (I think?) returned to the pool. This processes is repeated *A=3000* times.

**Response 2-13:**

It is true that most of the compartments were empty, and we often combined empty compartments. This procedure is certainly inefficient but not time-consuming because the calculation is very simple. Hence, omitting this procedure did not significantly improve the computational cost.

The two selected compartments were combined and divided before returning to the pool. The fusion and division steps were applied successively to the same compartments. Admittedly, our previous description of this procedure was unclear. We have clarified this point in the revised manuscript (the revised version is shown in the Response 2-12).

**Comment 2-14:**

▪ I have simulated a similar, yet simplified process. I initialized *C_S=750* compartments with random numbers and *2250* empty compartments. Then, I mixed the content of randomly chosen couples of compartments by taking the average, a process which I repeated *A=5000* times. After one such process, it was typical that *~280* compartments would remain empty. Then, *C_S=750* of the total compartments would be selected for a next round, carrying over some of the empty compartments. The pool would be filled up with *2250* new additional empty compartments. I have tracked the number of empty compartments over time and found the dynamics in the plots below.

▪ There are two reasons why the number of empty compartments changes over time (eventually, leading to an “all empty” state). On the one hand, each round, a set of empty compartments can be selected and increase the number of empty compartments in the next round. On the other, mixing non-empty with empty compartments tends to dilute the concentrations over time. This last problem should not affect so much the dynamics simulated in the manuscript, as diluted concentrations can come back up during the replication step (but I am not so sure about this, since certain interpretations of the experiments could actually worsen this problem). In any case, I am not so sure that these issues do not change the likelihood of ending up with empty compartments. My main worry is that the implemented dynamics are not only affected by the stability of the equations, but perhaps also by a highly ad-hoc resampling process.

**Response 2-14:**

In our understanding, the reviewer’s concern is that randomness in the compartment selection step could affect the stability of the replicator networks. We believe that while it could affect the stability, the effect should be minor because we performed each simulation multiple times (10 times in the previous manuscript) and evaluated the sustainability of each network based on the trends of the results. However, we agree that 10 was a small sample size. To analyze the effect of random sampling, we performed the stimulations 100 times, which has been indicated in the revised manuscript, as described in the Response 2-6.

**Comment 2-15:**

▪ I assume that this model attempts to replicate the wet-lab experiments from which the empirical data is obtained. It would be useful to have a succinct description of these experiments to see how the different phases of the model match up with the experiments.

**Response 2-15:**

Following the reviewer’s suggestion, we have included a brief description of the experimental procedure at the beginning and in the description of the fusion-division step (see Response 2-12).

**Comment 2-16:**

◦ This paragraph still presents some additional problems:

▪ In line 480-481: What does it mean precisely that “the number of replicators in the compartment was approximated from the concentrations”?

**Responses 2-16:**

This sentence indicates that before the fusion-division process, the amount of RNA in each compartment was defined as a concentration and not as a number, because RNA replication was calculated by differential equations. We then determined the number of RNA by rounding off the RNA concentration because we needed the number of RNA at the division phase to simulate the stochastic distribution of RNAs into the compartment. This explanation has also been added to the revised manuscript, as shown in Response 2-12.

**Comment 2-17:**

▪ In lines 481-483—specially troublesome:

• How are the replicators redistributed randomly into two new compartments? Is this decided in a one by one case for each of the replicators (e.g. for each replicator a coin is flipped)? (Note that the implementation of flipping a coin for each replicator would be very slow. This could contribute towards inefficiency and low number of replicas.)

**Response 2-17:**

Yes. The coin was flipped for each replicator. This point has been clearly described in the revised manuscript, as shown in Response 2-12. As the reviewer noted, this process is inefficient and approximation is possible, but this redistribution process is not a rate-limiting step. Hence, reducing the time for this step would contribute little to the total computational time.

**Comment 2-18:**

• The two compartments into which the replicators are redistributed: are they the same as the ones that got mixed up? Or are they two new randomly selected compartments from the pool? If so, are the two original compartments emptied when the replicators are redistributed to the new compartments? If not, at this step the number of replicators is being artificially multiplied by 2.

**Response 2-18:**

The two compartments into which the replicators were redistributed were the same as those that were mixed in the fusion step. This point has been clarified in the revised manuscript, as shown in Response 2-12.

**Comment 2-19:**

• I have run simulations of several variations of my toy mixture model taking into account the possible answers to these questions. I have observed transitions to “all empty” (as in the plot below) in all cases except when the number of replicators is artificially duplicated.

**Response 2-19:**

We thank the reviewer for evaluating the results. In our simulation, we did not perform artificial duplication of the replicators at the redistribution step. However, in our simulation, replicators replicated before the fusion-division step and were redistributed at the fusion-division step, thereby avoiding becoming “all empty.”

**Comment 2-20:**

• In lines 503-505:

◦ Values of the coefficients are listed. It does not hurt to indicate explicitly which are the *k_ij* for hosts and the *k_ij* for parasites, and (as commented above) to make these

symbols different.

**Response 2-20:**

Following the reviewer’s suggestion, we have changed the sentence and symbol for clarification as follows.

“Third, the coefficients of a new host (a new host *i* replicated by host *j* ($k_{ji}^{H}$)) were randomly chosen from 1 to 3 as a type of double-precision floating-point number. The coefficients of a new parasite *h* replicated by host *j* ($k_{jh}^{P}$) were randomly chosen from 0 to 10 as a double-precision floating-point number.”

**Results**:

**Comment 2-21:**

• In lines 151-152:

◦ I assume that, in this sentence, the Authors refer to the experiment described in section “Parameter estimation of the representative RNAs”, at the end of the manuscript. It would help if this section were mentioned explicitly. This manuscript bases a lot of their results in earlier experiments (from Refs. [29-31]), and it sometimes becomes difficult to track what comes from there.

**Response 2-21:**

Following the reviewer’s suggestion, we have explicitly described the referred section as follows.

“The four values are based on experimental data that were obtained in the later experiment conducted in this study (Table 1, estimated in the “Parameter estimation of the representative RNAs” section below), in which…”

**Comment 2-22:**

• In line 176:

◦ As in the previous point. It would help the reader if explicit references existed to the papers or sections where these parameters were obtained.

**Response 2-22:**

Following the reviewer’s suggestion, we have explicitly described the referred section as follows.

“For these replication coefficients, we used the same values as those used in the HH network (Table 1, estimated in the “Parameter estimation of the representative RNAs” section below), including the two extreme values (0.2, 1,7, 2.0, 2.3, 2.6, and 4.1).”

**Comment 2-23:**

• In lines 187-189:

◦ I am sorry, I don’t understand this sentence.

**Response 2-23:**

To clarify the sentence, we rewrote it as follows.

“The frequency of fusion-division, *A*, was also changed proportionally to *C* to keep the average fusion-division number per compartment constant.”

**Comment 2-24:**

• Fig. 4:

◦ I think that this figure can be eliminated.

**Response 2-24:**

We agree that the result of the HPP network in Fig. 4 presents less information because it just shows “no survival.” However, we believe that Fig. 4 will be useful for readers to visually grasp the trends of all networks. Without Fig. 4, readers must read the main text to understand the results of the HPP network. Therefore, we retained Fig. 4 as the main figure.

**Comment 2-25:**

• In line 221:

◦ Watchout for subscripts.

**Response 2-25:**

We have corrected the mistake in the revised manuscript.

**Comment 2-26:**

• In Fig. 5:

◦ Is panel D necessary?

**Response 2-26:**

The reviewer probably intends to question “panel E” instead of “panel D” in Fig. 5, because panel D is one of the most important panels in this manuscript. If so, we believe that panel E would be informative for readers to grasp the relationship among the replicators in a typical sustainable HHP network because each coefficient is represented as the color depth of arrows in the panel. We think that it is not necessary, but we would like to retain it to aid the readers.

**Comment 2-27:**

• In section “Computer simulation of the transition of the networks”:

◦ I find this section very confusing. On the one hand, I think that it is not well explained and that these simulations might be affected by the issues mentioned before—regarding sampling, division-fusion, etc. On the other hand, I do not understand the results, or I think that the obtained results seem meager.

**Response 2-27:**

In accordance with the reviewer’s comment, we have described the simulation procedure in more detail in the revised manuscript, increased the number of independent runs, and added an analysis of the RNA species in the final populations as described below (see Responses 2-28).

**Comment 2-28:**

◦ In lines 303-305:

▪ These lines are very confusing. I don’t know what is a round, a run, and a time.

**Response 2-28:**

To clarify the meaning of the words the reviewer mentioned and the simulation procedure and results, we have rewritten the paragraph as follows.

**Method section**

**“Evolutionary simulation**

A mutation step was introduced immediately before the replication step to simulate the evolutionary transition shown in Fig. 7. In this step, a new host is generated from existing hosts at a rate of 0.02 per replication, based on the mutation rate of Qβ replicase (36) and the typical RNA size (2000 nt). A new parasite is generated from a parasite at a rate of 0.002 per replication, because of approximately 1/10-fold smaller RNA size. Since we could not find reliable data for the generation rate of a new parasite from a host, we used the value of 0.001, which was smaller than the generation rate of a new parasite from the parasite. To reduce the computational cost, the total number of replicator species was restricted to less than three. In other words, a new species could appear in the mutation step only when the total number of replicator species is one or two. For computational ease, only one type of new replicator can appear in one round for both the host and parasites (i.e., the simultaneous appearance of two types of hosts was not allowed, but the simultaneous appearance of one type of host and one type of parasite was allowed).

When the total number of replicator species was less than three, the mutation process was conducted as follows. First, the number of replicates at the replication step was counted for each replicator in each compartment. Second, the probability of the appearance of a new replicator was calculated by multiplying the replication number with the mutation rate. If the probability defined for each replicator in each compartment was less than a random value $\in[0, 1]$, then a new host or parasite appeared in the compartment. The probability was typically very low, and thus, only one new host or parasite appeared in each round in most cases. However, for simplicity, if two new hosts or two new parasites simultaneously appeared, we assumed that the two new species were the same (i.e., had the same coefficients). Third, the coefficients of a new host (a new host *i* replicated by host *j* ($k_{ji}^{H}$)) were randomly chosen from 1 to 3 as a type of double-precision floating-point number. The coefficients of a new parasite *h* replicated by host *j* ($k_{jh}^{P}$) were randomly chosen from 0 to 10 as a double-precision floating-point number. The simulation was started from a single host that self-replicated with a coefficient of 2.0 and continued for 1000 rounds of serial replication cycles. The number of compartments ($C$) was 3,000, the selection rate ($S$) was 0.25, the fusion-division frequency ($A$) was 5,000, and the carrying capacity ($N$) was 100. The code for the simulation has been uploaded to GitHub (https://github.com/Dokunuma/PrebioDivSim).”

**Result section**

**“Computer simulation of transition of networks**

Next, we investigated the possible evolutionary transitions of networks using computer simulations. We introduced a mutation step into the serial replication cycle immediately before the replication step. During the mutation step, a new host or parasite appeared at a certain rate in one of the compartments if the total number of replicator species in the system was less than three. A new host was generated from existing hosts at a rate of 0.02 per replication, and a new parasite was generated from hosts and parasites at rates of 0.001 and 0.002 per replication, respectively. These generation rates were partially based on experimental data (see Methods for details). A new host or parasite had replication coefficients randomly chosen from 1 to 3 or 0 to 10 as a type of double-precision floating-point number, respectively.

Starting with a single host species ($k_{11}^{H}$ = 2.0), we performed 1000 rounds of serial replication cycles 1000 times. We counted the number of networks that were maintained for more than 100 rounds during the replication cycles and found that HPP, HHP, and HHH networks were maintained in 19, 218, and 14 runs, respectively (Fig. 7A). This finding indicated that the HHP network was easier to maintain than other three-member networks, which was consistent with our sustainability analysis (Figs. 4-6). To investigate the network from which the HHP network was formed, we examined the network immediately preceding the 218 HHP networks. Most (153) of the HHP network was preceded by HP networks (Fig. 7A), supporting the hypothesis that the transition from HP to HHP network is the most plausible route. Fig 7B shows a typical example of the population dynamics in the simulation that reached the HHP network. An HP network was formed around round 520, and then it became an HHP network around round 560.

Next, we investigated the coefficients of 218 HHP networks maintained in the evolutionary simulation. According to the sustainability analysis shown in Fig. 5, the HHP network was sustainable when two hosts showed asymmetric resistance to the parasite, and non-resistant hosts replicated more than resistant hosts (i.e., $k_{11}^{P}$ > $k_{21}^{P}$ and $k_{11}^{H}$ + $k_{21}^{H}$> $k_{12}^{H}$ + $k_{22}^{H}$). We found that 170 out of 218 HHP networks satisfied this condition. This consistency between evolutionary-determined and stable parameter values has been suggested previously to support the reliability of sustainable conditions (35).”

**Comment 2-29:**

▪ I find it very odd that all replicators die off in 97 (out of 100?) runs. I think that the implementation of the dynamics (as discussed in my issues with the Methods section) might be playing a very crucial role.

**Response 2-29:**

We agree that the low survival rate in the simulation seems strange because, in other studies, we performed evolutionary experiments several times, but RNA was never eliminated. RNA maintained a robust presence in the experiments. We believe that this contradiction can be attributed to the much smaller number of compartments in the simulation (3000) compared to that in the experiment (10^8^). With such a small number of compartments, stochasticity in the selection and RNA distribution would accidentally lead to extinction. To support this hypothesis, when we simulated a smaller or larger number of compartments, the survival rates increased (i.e., the disappearance rate decreased) as the number increased. We have added these simulation results and explanations to the revised manuscript as follows:

“During 1000 rounds of replication cycles, all replicators disappeared in 796 of 1000 simulations. This high rate of disappearance was seemingly contradictory to the experimental results, in which we have never experienced disappearance of all RNA species (32). This contradiction can be explained by the differences in the number of compartments. In this evolutionary simulation, we used 3000 compartments, whereas the experiment had approximately 10^8^ compartments. If the number of compartments decreases, the chance of accidental extinction of RNA species should increase. To verify the effect of the compartment number, we performed the same evolutionary simulation with smaller (1000) and larger (5000) number of compartments, in which the disappearance rate increased (936 disappearance in 1000 simulations) or decreased (470 disappearance in 1000 simulations), respectively. This finding supports that the larger disappearance rate in the simulation can be explained by the smaller number of compartments.”

**Comment 2-30:**

▪ Can we sustain the claim that HPH is a pathway to complexification if 97 out of 100 replicas simply died?

**Response 2-30:**

We believe that HHP is a plausible complexification pathway, even if the replicators disappear in most of the cases as long as the majority of the surviving RNAs form the HHP network. However, we admit that the number of sustained runs was too small, and hence, we increased the number of evolutionary simulations up to 1000 with more realistic parameter sets. We observed 204 times survival (796 times disappearance) in this case. This new simulation results have been described in the revised manuscript (see Response 2-29 above).

**Comment 2-31:**

◦ In Fig. 7:

▪ According to the methods (line 498), hosts mutate with a chance 0.5 such that, at each iteration, half of the existing hosts should become a new host. But we do not see mutants popping up at each iteration. On the one hand, such a high mutation rate is not realistic if we wanted to remain in the “population dynamics” limit. On the other hand, what is going on with the simulations at this point? Why don’t we observe the maximum number of species (3) in every round?

**Response 2-31:**

In Fig. 7 in the previous manuscript, the popping up of new mutants were not shown because we represented only replicators with concentrations of more than 3000 (i.e., minor species were omitted). Hence, we did not observe the maximum number of species (3) in every round. Because such a representation may be misleading, we have shown all replicators in the revised Fig. 7B (shown below).

We agree that the mutation rate was unrealistically high; thus, we used more realistic values (0.02 for a host to turn into a mutant host, 0.001 for a host to become a parasite, and 0.002 for a parasite to become a mutant parasite) based on experimental evidence in the revised manuscript. This point has been newly described in the revised manuscript (see Response 2-28 above).

**Figure 7. Computer simulation of the evolutionary transition of replication networks.**

The evolutionary transition was simulated by introducing a mutagenesis step in the serial replication cycle, as shown in Fig. 2, for 1000 rounds. A) The number of networks maintained for more than 100 rounds during replication cycles in 1000 simulations was counted. B) A typical trajectory of the concentrations in one of the simulations that resulted in the formation of the HHP network.

**Comment 2-32:**

▪ Also from the Methods (lines 499-500) and Results (lines 298-300), “the total number of replicator species is restricted to less than three”. Might this be what is limiting, artificially, the appearance of new species and alternative routes to stability? If new mutants are prevented from popping up, we are biasing the system towards HPH for complexification. Indeed, we observe HH coexisting for hundreds of rounds. We even observe HHH for dozes of rounds, if I’m interpreting Fig. 7 correctly. Shouldn’t these configurations be rather unstable?

**Response 2-32:**

We agree that the limit of the total number of replicator species of up to three might have biased the results to some extent. However, we believe that the simulation under this limitation is sufficient to evaluate the stability of the up-to-three-member networks because even under this limitation, a new replicator can appear if a newly formed three-member network is unstable and some replicators are diluted out. Therefore, under this limitation, the replicator network should be transient until it becomes stable. We do not think that this is the best simulation, but we think that it is sufficiently informative to support our conclusion in this study (i.e., a stable HHP network).

In addition, the reviewer wrote that he or she observed the HHH network for dozens of rounds in the comment, but we think this is a misinterpretation. In the previous simulation, the HHH network was hardly found; at least one parasitic replicator (colored red or pink) exited each time. Same findings were observed with the new simulation depicted in the revised manuscript (see Fig. 7A shown above).

**Comment 2-33:**

◦ Some limitations and comments about this simulation:

▪ I understand that the authors limited the species to 3 for computational issues (it becomes unfeasible to study the stability of their model as more species are introduced).

▪ On the other hand, I understand that they needed to limit the number of parameter values that they evaluated in order to asses the stability. However, this later point is not necessary when simulating the dynamics in this section. Actually, as the experiments make very clear (and I will discuss below) it is reasonable that coefficients for new hosts and parasites will not take values over the discrete set of parameters, but over the continuum in which parameters live. Actually, through Darwinian selection, it is more likely that sets of parameters will be observed within the region where dynamics are stable.

▪ If we remove these artificial constraints, the picture in which the HPH route to complexification is preferred does not seem so clear. With such high mutation rates, it is more likely that many more hosts and parasites emerge and decay at every timestep. If the mutation rate were lower, it remains very likely that stable HH ecosystems form following Darwinian elimination of unstable host partners.

▪ All in all, I am not convinced that this simulation strengthens the hypothesis that HPH is the most reliable route to complexification—most of all because it is based on 1 out of 3 simulations versus the 97 simulations that did not even remain stable.

**Response 2-33:**

We agree that the previous conclusion on one out of three simulations versus 97 simulations was not convincing. Thus, we performed 1000 evolutionary simulations with more realistic parameters in the revised manuscript and found that the HHP network was sustained for at least 100 rounds in 218 of 1000 simulations (new Fig. 7A, shown in Response 2-31). We also found that the precedent network that led to the HHP network was mainly the HP network. In this simulation, each replicator used more continuous parameter values instead of several discrete values used in the previous simulation. In addition, we used more realistic mutation rates, as described in Response 2-31. This result was consistent with our hypothesis that a replicator network tends to develop into an HP network and then changes to an HHP network if the replicators avoid extinction. We hope that these new results will resolve the reviewer’s concerns.

**Comment 2-34:**

• In section “Host and parasitic RNAs that may form HP and HHP networks in the previous evolutionary experiments”:

◦ In this section, it shows up my greatest discrepancy with how the Authors interpret their experiments. They seem to adopt a “population dynamics” perspective (and I apologize if I am wrong about this point). In turn, Fig. 8 strongly suggests, in my opinion, that we are looking at a quasispecies dynamic. It is difficult for me to view each sequence as a different species of host or parasite popping up into existence, and doing so feels like attempting to force the data into the computational model.

**Response 2-34:**

We understand the reviewer’s interpretation and agree that the perspective of quasispecies dynamics is needed to explain some parts of the experimental results. However, we believe that the quasispecies dynamic view alone cannot explain all parts of the results. We believe that both views are required to precisely interpret our experimental results, as described in the following responses.

**Comment 2-35:**

◦ What we seem to have, instead, is a cloud of mutants that are accessible from each-other as connected in a genotype-genotype network. Hosts are at distances 3 and 4 from the original RNA sequence, and they are at only 7 mutations from each other. Meaning that both hosts should be relatively accessible from each other. What are the mutation rates of the sequences in this experiment? How many unique sequences are observed in each round? From my experience with similar experiments, I bet that mutation rates are very high and that there are a lot of different unique sequences. I further bet that there, in the population, a lot of the intermediate steps between both hosts are abundantly represented. Meanwhile, the hosts are at a distance 11 in the dendogram. Does this make sense, provided they are only 7 mutation apart? This suggests that a phylogeny is not appropriate to observe this kind of Darwinian process—rather, as I said, a genotype-genotype network seems more appropriate.

**Response 2-35:**

First, we answer the question in the comment.

Question 1: “What are the mutation rates of the sequences in this experiment?”

Answer 1: The mutation rate in the experiment was approximately 9.1 × 10^-6^ per nucleotide (García-Villada and Drake, PLoS Genet. 2012). As the host RNA comprises 2125 nucleotides, the host RNA should accumulate about 0.02 mutations in every replication. This value was used in the evolutionary simulation described above.

Question 2: ”How many unique sequences are observed in each round?”

Answer 2: We do not know the number of unique mutations because they cannot be detected using the sequencing method (Pacbio RS) used here. Using this method, we can detect only dominant mutations that exist in more than 10% of all RNAs in the population due to relatively high sequencing error.

Question 3: “Meanwhile, the hosts are at a distance 11 in the dendrogram. Does this make sense, provided they are only 7 mutation apart?”

Answer 3: Yes, this is possible because the phylogenic tree (dendrogram) is an approximation of phylogenic relationships, which is sometimes inaccurate, especially when multiple sequences are separated at the same distance. For example, the phylogenic tree of four two-digit sequences (AA, AG, AT, and AC), all of which are separated by a Hamming distance of 1, is as follows: In this tree, AA appears at a distance of 3 from AT.

Therefore, we think that the phylogenic tree is sometimes misleading especially for sequences at the same distance, such as quasispecies. The reviewer proposed a genotype-genotype network instead of a phylogenic tree for the representation of such quasispecies. We agree with the idea and have used a type of genotype-genotype mapping in our previous study (e.g., Fig. 2C in Furubayashi et al, eLife, 2020, also shown below), in which the genetic distances of RNAs were represented by the principal coordinate analysis (PCoA).

**Fig. 2c in Furubayashi et al 2020**

However, we chose to use the phylogenic tree in the next (Mizuuchi et al, Nat comm, 2022) and this studies because we noticed that the RNA species were gradually separated as distinct lineages beyond the range of quasispecies. To represent such separating lineages, we think that the phylogenic tree is more intuitive and easier to understand.

The reason we think the RNA species ($\mathrm{Host}_{1}^{exp}$ and $\mathrm{Host}_{2}^{\exp}$ in this case) should be regarded as members of distinct lineages and not the same quasispecies is that the mutation rate (0.02 per RNA per replication) was still too low to include $\mathrm{Host}_{1}^{exp}$ and $\mathrm{Host}_{2}^{exp}$ in the same quasispecies. According to our understanding, quasispecies are populations of genetic variants that show interchangeability with a few random mutations. The size of quasispecies depends on the mutation rate; if the mutation rate is high, the quasispecies should be large. The size of the quasispecies also depends on the population size of the system, because the size of the quasispecies is never larger than the population size. In the experimental system, the population size is 10^9^ at the maximum. Given that the possible point mutations for host RNA with a length of approximately 2000 nt is approximately 6000 (i.e., three possible mutations for each nucleotide), the possible variants at the Hamming distance of 2 and 3 are approximately 3.6 × 10^7^ (i.e., 6000×6000) and 2×10^11^ (i.e., 6000^3^), respectively. Even if a single quasispecies occupies the whole population, it can cover all variants at a Hamming distance of 2 and only a small fraction of the variant at a Hamming distance of 3. Therefore, we think that the $\mathrm{Host}_{1}^{exp}$ and $\mathrm{Host}_{2}^{exp}$, which are separated at a Hamming distance of 7, are unlikely to be included in the same quasispecies. Hence, we think that it is more appropriate to regard the $\mathrm{Host}_{1}^{exp}$ and $\mathrm{Host}_{2}^{exp}$as separated species through the Darwinian selection process.

We think that the $\mathrm{Host}_{1}^{exp}$ and $\mathrm{Host}_{2}^{exp}$would be better represented as a separate lineage with a phylogenic tree, while at the same time, we feel that this representation alone cannot explain parts of the results, such as many small branches of the parasites and host species, as the reviewer has pointed out in the next comment. Hence, we agree that we should discuss the effects of quasispecies. Accordingly, we have rewritten the corresponding sentences and the Discussion section to describe the possible contributions of quasispecies (see Response 2-36 below for details).

**Comment 2-36:**

◦ If this view is adopted, several of the preceding issues disappear. For example, there is no competitive exclusion because each sequence is generating nearby neighbors in the network by frequent mutation. Viable mutants are fairly close to their ancestors, thus they naturally contribute to cross-reproduction as modeled by the *k_ij* between different hosts. Also, coefficients of new mutants might be close to older ones, favoring stable configurations that are selected in a Darwinian fashion. Note that, if we insist on the population dynamics perspective, the experiments show rather stable coexistences between several hosts, which should be unstable according to the stability analysis. Do the Authors believe that there is a parasite in Fig. 8 for each possible host, and that they have been introduced as reported in Sup. Fig. S5? Isn’t it much more parsimonious to assume that we are looking at a quasispecies and that a broad range of parasitic strains is sustained together with a similarly broad range of able strains?

**Response 2-36:**

We are thankful for the reviewer’s comment. We agree that the small branches within the parasite population (Fig. 8A) and branches 1 and 2 (Fig. 8B) are better interpreted as quasispecies. RNA variants that form small branches can frequently interconvert with each other. However, as explained in Response 2-35, we think that the large branches between branches 1 and 2 should be regarded as separate lineages. We believe that both views are necessary to appropriately interpret the experimental results shown in Fig. 8. Therefore, we have added an explanation in the corresponding Results section and a new paragraph in the Discussion section as follows.

**Result section**

“Notably, other than the large branches between branches 1 and 2, several small branches were observed within branches 1 and 2 (Fig. 8B) and also in the phylogenic tree of parasites (Fig. 8A). These small branches are expected to be derived from quasispecies. The interpretation of this result based on quasispecies is discussed later.”

**Discussion section**

“Another important factor in interpreting the experimental results is quasispecies, a population of variants with random mutations. The mutation rate of RNA replication by Qβ replicates is high (approximately 9.1 × 10^-6^ per nucleotides) (García-Villada and Drake, PLoS Genet. 2012), corresponding to approximately 0.02 mutations during every replication of host RNA. This high mutation rate produces a population of genetic variants around an RNA genotype. As shown in Fig. 8A, many small branches were observed within the parasite population. In the host population (Fig. 8B), we also observed many small branches within branches 1 and 2 (Fig. 8B). The existence of many different but closely related genotypes can be explained by quasispecies. $\mathrm{Host}_{exp}^{1}$ and $\mathrm{Host}_{exp}^{2}$were not expected to be characterized as the same quasispecies because they were separated by a Hamming distance of 7, while the ancestors of these host RNAs were expected to be characterized as the same quasispecies at the root of the branches.”

**Comment 2-37**

◦ As I said above, I am very sympathetic to this work and I hope that some version of it gets published in a very good journal. Unfortunately, I have the strong feeling that the Authors are forcing a partial interpretation of Darwinian dynamics that, I think, simply does not apply in this experiment. The limit in which population dynamics applies has no crossover between competing species, while here it is obvious that one host can be reached from the other in a few rounds of the experiment.

**Response 2-37:**

We appreciate the reviewer’s comment, which has broadened our view of our experimental results. As described above, we believe that many small branches within host branches 1 and 2 can be explained by quasispecies dynamics, and large branches between branches 1 and 2 can be explained by Darwinian dynamics. We believe that this is the most reasonable explanation at this stage.

**Comment 2-38:**

• In Fig. 9:

◦ In a final comment regarding the population dynamics versus quasispecies interpretation of the data:

▪ The experiment reported in Fig. 9 runs for 25 rounds. In Fig. 8B, that seems to be the time that it took the original RNA strain to jump from the ancestral branch to branch 2. (This is only apparent, since we do not have access to intermediate samplings of the experiment.) Still in Fig. 8B, we then observe a fast diversification in rounds 33 and 39, which is less time than the 25 previous rounds.

▪ I find it very likely that a similar phenomenon might go on, should the experiment in Fig. 9 be continued for a few more rounds. Furthermore, while the stability is attributed to the HPH configuration, I think that it is more likely that both hosts and the parasite are actually accompanied by an unobserved cloud of mutants, and that their stability stems from quasispecies dynamics. These hosts and parasite, in

principle, already belonged to a rather mature quasispecies, which they might have

been able to recreate.

**Response 2-38:**

We agree that during the experiment shown in Fig. 9C, the quasispecies of the $\mathrm{Host}_{exp}^{1}$, $\mathrm{Host}_{exp}^{2}$, and $\mathrm{Parasite}_{exp}$would appear and might contribute to sustainable replication. We think both interpretations (quasispecies dynamics or Darwinian population dynamics) are required to explain our result. However, we would like to emphasize here that the two different interpretations are not the focus of this study, nor do they affect the conclusion of this study, a plausible route of host and parasite replicator network. As the reviewer noted, this was a matter of interpretation.

Finally, we would like to thank the reviewers for their efforts in reading our manuscript and providing insightful evaluation via comments. We have learned a lot from their comments. We hope that the revised manuscript will address these concerns.
