# Supplementary material for: Plausible pathway for a host-parasite molecular replication network to increase its complexity through Darwinian evolution
Source: PLoS Comput Biol. 2022 Dec 1;18(12):e1010709. doi: 10.1371/journal.pcbi.1010709 (PMC9714742; doi:10.1371/journal.pcbi.1010709)
Supplement: Attachment — Submitted filename: 220924_point-by-point_Kamiura_4.docx [file pcbi.1010709.s022.docx]

Point-by-point responses

**Reviewer #1:**

**Comment 1**

I thank the authors for their revision. The revision has addressed the issues I raised with the positioning of the authors' work in the context of existing work.

Reading the whole manuscript again, I noticed a few additional points that appear to be important enough to raise, even though this is the second review.

**Response 1:**

We are again thankful for the reviewer’s time and effort in evaluating our manuscript. We addressed the points raised by the reviewer in the revised manuscript. The detailed responses are shown below.

**Comment 1-1:**

Lines 98-107 contain duplicated sentences.

**Response 1-1:**

We removed the duplicated parts in the revised manuscript.

**Comment 1-2:**

Line 279-281 says, 'in the parameter region of k^H_22=2.0 (red rectangle in Fig 5D), the region with 100 points increased from left to right (in the direction of increasing k^H_11).' However, this statement does not appear to be correct, according to Figure 5D. The figure seems to show that the number of regions with 100 points barely changes with k^H_11: 8 regions (k^H_11=1.7), 8 regions (k^H_11=2.0), 7 regions (k^H_11=2.3), and 8 regions (k^H_11=2.6). What actually happens is that the range of k^H_12 for which HHP persists when k^H_21=2.0 shifts upwards as k^H_11 increases.

**Response 1-2:**

Thank you for pointing out our mistake. After carefully examining the simulation results and programming code, we found that some numbers in the previous Fig. 5D were assigned to the wrong positions because of a bug in our program. The bug affects only a few data points in HHP and HHP results and thus, there is almost no effect for all the statements in the Result section and the conclusion of this study. In the revised manuscript, we corrected the bug and exchanged HHP and HHH results (shown below). We apologize for the confusion caused to the reviewer based on our mistake. We verified that the other results were not affected by this bug.

These new HHP and HHH data are consistent with the explanation in the Results section, but we found that some points were inaccurate. Thus, we rewrote the explanation as shown below (changes are underlined).

**Explanation of HHP result in the Results section:**

“We examined the asymmetric case in more detail. First, we found that as the self-replication of Host 1 (non-resistant host) increased (i.e., $k_{11}^{H}$ increased), the number of sustainable runs gradually increased. For example, in the parameter region of $k_{22}^{H}=2.0$ (red rectangle in Fig. 5D), the region with more than 90 points increased from left to right (in the direction of increasing $k_{11}^{H}$). By contrast, as the self-replication of Host 2 (resistant host) increased (i.e., $k_{22}^{H}$ increased), the number of sustainable runs decreased. For example, in the region of $k_{11}^{H} = 1.7$ (blue rectangle), the region with 100 points significantly decreased from bottom to top (in the direction of increasing $k_{22}^{H}$). Second, we found that as the cross-replication of Host 2 to Host 1 ($k_{21}^{H}$) increased, the number of sustainable runs increased. For example, in the region of $k_{11}^{H}=2.0 \mathrm{and} k_{22}^{H}=2.0$ (green rectangle), the points increase from left to right (in the direction of increasing $k_{21}^{H}$). In summary, a sustainable asymmetric HHP network requires parameter sets that favor replication of the parasite-susceptible host either by self-replication or cross-replication (a typical condition is schematically depicted in Fig. 5E).”

**New Figure 5. Search for the parameters that allow sustainable HHP networks.**

Symmetric (A) and asymmetric (C) HHP networks. The number of runs in which all three replicators (Hosts 1 and 2, and Parasite 1) were sustained for 100 rounds out of 100 independent simulations in symmetric (B) and asymmetric (D) cases is shown. The magenta square represents the close parameter values of the representative RNAs obtained from the evolutionary experiment. (E) A typical condition for a sustainable HHP network, which contains a parasite-susceptible and parasite-resistant host species; the parasite-susceptible host (Host 1) tends to replicate more efficiently through self- and/or cross-replications. The color depth of the arrows represents the value of the replication coefficients.

**Explanation of HHH result in the Results section:**

“Under the “low self- and high cross-replications” conditions (Figs. 6B and 6C), sustainable parameter sets were frequently found when $k_{33}^{H}$ is the smaller value, 1.7 (Fig. 6B), whereas rarely found when $k_{33}^{H}$ is the larger value, 2.6 (Fig. 6C), indicating that low self-replication also for the new Host 3, which induces inter-dependent replication of all replicators, is important for the sustainability.

A similar trend was also found under the “balanced replication” conditions (Figs. 6D and 6E), the three hosts sustainably replicated with a certain range of parameter space with the smaller $k_{33}^{H}$ values (Fig. 6D), whereas such parameter sets were rarely found with the larger $k_{33}^{H}$ values (Fig. 6E). We found that when the parameters for Host 3 are also “balanced” (i.e., $k_{31}^{H}$ = $k_{32}^{H}$), the HHH network tended to be sustainable under the conditions.”

**New Figure 6. Search for the parameters that allow sustainable HHH networks.**

(A) Scheme of the HHH networks. (B-E) Numbers of runs in which all three hosts are sustained for 100 rounds out of three independent simulations. The parameter values for Hosts 1 and 2 are fixed at two cases that allows sustainable replication in the HH network. (B, C) Conditions I (Fig. 3C, $k_{11}^{H}$ = 1.7, $k_{21}^{H}$ = 2.6, $k_{12}^{H}$ = 2.0, and $k_{22}^{H}$= 1.7). (D, E) Conditions II (Fig. 3D, $k_{11}^{H}$ = 2.0, $k_{21}^{H}$ = 1.7, $k_{12}^{H}$ = 2.0, and $k_{22}^{H}$ = 1.7). For the same reason, we employed a small (1.7) (B and D) or a large (2.6) (C and E) value for the self-replication coefficient of the newly added Host 3 ($k_{33}^{H}$).

**Comment 1-3:**

I am not sure if the statement in Lines 288-289 is accurate. The statement says, 'In summary, a sustainable asymmetric HHP network requires parameter sets that favor replication of the parasite-susceptible host either by self-replication or cross-replication.' This statement could be interpreted as saying that the survival of HHP is facilitated by increasing k^H_11 or k^H_21. Interpreted this way, the statement does not appear to be true. Figure 5D shows that the survival probability of HHP is 0.96 when k^H_11=2.3, k^H_12=2.0, k^H_21=2.0, and k^H_22=2.0. Compared to this parameter set, increasing k^H_11 to 2.6 decreases the chance of survival to 0.08, which contradicts the above interpretation of the statement.

**Response 1-3:**

We admit that the previous statement was inconsistent with the result due to the incorrect Fig. 5D as mentioned in Response 1-2. With the correct Fig. 5D, the statement was almost valid, but we slightly changed it to be more accurate (changes are underlined).

“In summary, as a rough trend, a sustainable asymmetric HHP network requires parameter sets that favor replication of the parasite-susceptible host either by self-replication or cross-replication (a typical condition is schematically depicted in Fig. 5E),”

**Comment 1-4:**

Similar to the above statement, the sentence in Line 358 says, "the HHP network was sustainable when ... non-resistant hosts replicated more than resistant hosts (i.e., k^P_11>k^P_21 and k^H_11+k^H_21 > k^H_12+k^H_22).' The inequality implies that the survival of HHP is enhanced by increasing k^H_11 or k^H_21 or decreasing k^H_12 or k^H_22. This proposition does not seem to be correct either. Figure 5D shows that the survival probability of HHP is 0.96 when k^H_11=2.3, k^H_12=2.0, k^H_21=2.0, and k^H_22=2.0. Compared to this parameter set, decreasing k^H_12 to 1.7 decreases the probability of survival to 0.07. The above results, together with the previous paragraph, appear to suggest that a sustainable HHP network requires parameter sets for which H1 is sufficiently fitter than H2 in the absence of P, but not too fit to drive H2 to extinction before P increases to give H2 an advantage.

**Response 1-4:**

We admit that the previous description was inaccurate for some regions, as the reviewer commented, even for the new Fig. 5D. We rewrote the corresponding part to be more accurate as follows.

“According to the sustainability analysis shown in Fig. 5, as a rough trend, most of the HHP network was sustainable when the parameters satisfied three conditions: 1) two hosts showed asymmetric resistance to the parasite (i.e., $k_{11}^{P}$ ≥ $k_{21}^{P}$), 2) the resistant host replicates the non-resistant host more than itself (i.e., $k_{21}^{H}$≥ $k_{22}^{H}$), and 3) the non-resistant host replicates equal to or more than resistant hosts (i.e., $k_{11}^{H}$ + $k_{21}^{H} \geq$ $k_{12}^{H}$ + $k_{22}^{H}$). We found that 148 of 218 HHP networks satisfied this condition.”

**Comment 1-5:**

Finally, the mutation in the authors' model is restricted such that there can be at most three "species" of replicators in the system. This restriction appears unrealistic, especially in view of the fact that the novelty of the model is said to lie in the availability of the corresponding experimental system, where mutations are not restricted. The assumed restriction on mutations raises an important question: are the modelling results robust if mutations are allowed to occur irrespective of the number of species? I suggest that the authors discuss this question because the assumed restriction seems quite strong. If mutations are allowed to occur irrespective of the number of species, host replicators might evolve towards maximising their self-replication coefficients, resulting in a situation where k^H_11 and k^H22 are nearly equal to each other. In this case, HHP would not be able to persist because H1 could not out-compete H2 in the absence of P. Likewise, parasitic replicators might evolve towards maximising both k^P_11 and k^P_21. In this case, HHP would not persist because H1 is not more susceptible to P than H2 is.

**Response 1-5:**

We admit that the evolutionary simulation that we conducted here has a large limitation. In accordance with the reviewer’s suggestion, we added a new paragraph to explain the limitation and robustness of the evolutionary simulation, as shown in Response 1-6.

**Comment 1-6:**

The following is an additional comment related to the question posed above. The authors' model does not incorporate any genotype-phenotype map and assumes that k^H_22 mutates in the same way as k^H_11 does (i.e., random walks between 1 and 3). By contrast, in the authors' experimental system, the genotype-phenotype map is likely to be complex, and the kinetic parameters of replicators of different "species" certainly do not mutate in the same way. The genotype-phenotype map might play a role in the evolution of HHP because it can impose various trade-offs, e.g., between being resistant to parasites (i.e., decreasing k^P_21) and maximising the self-replication rate (i.e., increasing k^H_22) for host replicators, or between parasitising H1 (i.e., increasing k^P_11) and parasitising H2 (i.e., increasing k^P_21) for parasitic replicators. Such trade-offs, if they existed, could prevent H2 from evolving a replication coefficient as high as that of H1 and, similarly, prevent P from parasitising both H1 and H2, thereby allowing the persistence of HHP. Thus, a complex genotype-phenotype map and consequent trade-offs might play a role in the evolution of HHP in the experimental system, which is not taken into account in the authors' model.

**Response 1-6:**

We agree with the reviewer’s comment. The evolutionary simulation conducted here uses a very simplified model, which has limitations in terms of the maximum number of possible replicators and the lack of genotype–phenotype linkage. To describe these limitations and their possible effects on the outcome, we added a new paragraph to the Discussion section as follows.

“We used a very simplified model in the evolutionary simulation conducted in Fig. 7, which allows for only three types of replicators at a time to save computational costs and does not include the trade-off between the replication coefficients because of the lack of knowledge of the mechanism underlying the trade-off. Owing to these limitations, the result of the simulation should be carefully interpreted. For example, the HHP network was maintained in 218 of 1000 runs in the simulation, but this number might be overestimated. With the limitation of the three types of replicators, once a stable HHP network appeared, no new replicator could invade the network. However, in a more realistic case without such a limitation, a new replicator that has a faster self-replication parameter might invade and destabilize the network. Furthermore, the probability of the appearance of such a faster replicator should depend on the trade-off between the coefficients. To perform a more realistic evolutionary simulation, further effort to minimize the computational cost and understand the trade-off between parameters would be needed.”

**Reviewer #2:**

**Comment 2-1:**

I have now read the revised version of this manuscript, as well as the detailed responses by the

Authors, and the report by the other Referee. There are several points about this manuscript that I find troublesome. Much of the disagreement comes from different interpretations of the experimental data, but I do not think that it is fair to use my leverage as a Referee to attempt to impose a viewpoint. I also feel that these issues will not be resolved by any amount of further reviewing. I add comments about these issues below, hoping that they might improve our understanding of the experiments and evolutionary dynamics in general. Apart from these points in question, I think that the work is mostly technically sound.

**Response 2-1:**

We are grateful for the reviewer’s time and effort in reading and commenting on our manuscript. We also appreciate the reviewer’s fair review and appreciation of the soundness of our study irrespective of our disagreement with respect to the interpretation. We agree with the reviewer’s comment, “these issues will not be resolved by any amount of further reviewing,” but as a final attempt, we rewrote the corresponding part of the Discussion section in the revised manuscript to our best ability as shown in Response 2-3.

**Comment 2-2:**

There are other aspects of the paper with which I take issue, and which, I think, can be solved with further minor editing. My comments on this respect are attached below as well, hoping that they will be addressed before a foreseeable publication of the paper. I hope that my comments are useful in improving the manuscript.

**Response 2-2:**

We are also thankful for these comments. We addressed the concerns in the revised manuscript as explained in the following responses.

**Comment 2-3:**

Major concerns:

The reference indicated by the other Referee (Takeuchi & Hogeweg, 2008) takes away a great part of the novelty that I though this work presented. I was not familiar with that paper before. Honestly, I have had manuscripts rejected for much less overlap with earlier research. However, I feel that rejecting on such a basis would be unfair. Things are re-discovered all the time, and the literature is often too vast to know every piece of work. I do take issue with the fact, pointed out by the other Referee, that the Authors seemed to misrepresent or diminish the findings by Takeuchi and Hogeweg in the earlier version. That could not come from a lack of familiarity with their paper. However, willingly or not, this has now been corrected.

Once that that part of the novelty has been somehow taken away, we are left with the actual contribution of this manuscript: To work out mathematically, and with allegedly realistic parameters, a situation that has purportedly taken place in the reported experiment. Unfortunately, I do not think that what the Authors model is what has happened in their experiment at all. Again, this is a matter of interpretation, and we might not have got sufficient empirical evidence to settle the question. I think that the modeling effort and the discussion is illuminating, and that it should be available for future debate. I think that the mechanism proposed by Takeuchi and Hogeweg, and modeled here, might be operating at some scale in evolutionary dynamics. I also think that some (much more complex) version of this mechanism might be operating within the evolutionary history of this experiment. However, I do not think that the empirical evidence can support that the reductionist view presented here is what has indeed happened in the experiment.

To tackle my earlier issues regarding interpretation, the Authors say that they have now included a discussion in terms of quasispecies. That is not true. The Authors have made some

superficial comments about quasispecies—not a discussion of the experiment from this framework. It would be unfair to ask the Authors to include a discussion with which they are not comfortable— in any case, I think that this was a nice opportunity that has been missed.

Focusing on Fig. 8, very likely we do have a huge cloud of mutants around few, very fit RNA strains. That would be a unique quasispecies. It is a very big simplification to take an individual strain as a representative of each branch, and propose that we have seen a sequence H→HP→HPH. Within each branch, we do have very abundant nearby representatives. The crossed replication coefficients in Fig. 9 are of a similar order of magnitude as the auto-replication coefficients—and this is for representatives across branches, just imagine how large the contribution of nearby mutants might be. What is more likely going on is that vast clouds of host- and parasite mutants sprawl from very early and simultaneously. This would result, almost from the beginning, in large networks including host- and parasite-variants that coexist over time—in a well sustained coexistence, indeed. Then, selection would operate, potentially reducing the abundance of variants into more stable subsets. This picture is radically different from the H→HP→HPH pathway— which appears meager from this perspective. This is what I would expect from a discussion of the experiment in terms of quasispecies. The comments by the Authors do not address any point from this picture.

Let us run some numbers from the data on mutation rate that the Authors have provided. According to empirical data, we would have ~0.02 mutations in each single sequence replication (focusing on hosts only). The experiment has 10^8 compartments. A strain that is present in all compartments would thus generate 2 million strings with mutations after replicating only once (let us call this “one replication step”). (Note that, likely, there are more than one single strain in each compartment. The variety that I calculate is thus a very low estimate.) The resulting mutated strings are likely to have one mutation only, thus they cover a fraction of genotype space at distance 1. Each Replication Cycle of the experiments (Fig. 2B) very likely contained several such replication steps (is there an estimate of this number?). Each such replication step would generate such huge variety (~2 million mutated variants, at the very least), and this novelty will be compounded with each step within one Replication Cycle. Mutations from the second generation are unlikely to walk back, thus their descendants will likely be at distance 2 from the original sequence. It is enough to have 7 replication steps within each Replication Cycle (which seems possible) to be able to explore a neighborhood at distance 7 of the original sequence.

I think that the criterion used by the Authors to argue that their two branches would not　belong to a same quasispecies is flawed. Genotype space is vast, and very unlikely to be covered by any cloud of mutants. This does not mean that parts of that space are not mutually accessible in a few steps. The second host (H_2) is definitely accessible in a very short time from the first host (H_1). How did H_2 evolve? Given Fig. 8B, I see two distinct possibilities: i) The whole branch 2 is actually present throughout the experiment (only not at detectable concentrations after round 24). This would add up to the interpretation that we have a single quasispecies at any time, with variants that can spring to prominence at any moment. ii) Within a few replication steps, H_2 evolved from H_1, thus illustrating the fact that both branches are mutually accessible. This would also point out why dendrograms might not be the best ceoncptualization (since H_2 in this case would evolve from H_1, not from others in branch 2). This reflection is included just as a speculation, to illustrate the rich picture that is left out.

Again, I do not think that we will bridge the gulf that separates or views, and I still think that the scientific work in this paper is sound (specially the experiments) and that the discussion can still be useful (my view might be the incorrect one after all).

However, I wanted to end this part of the review with a final comment. In their response, the

Authors wrote: “However, we would like to emphasize here that the two different interpretations are not the focus of this study, nor do they affect the conclusion of this study, a plausible route of host and parasite replicator network. As the reviewer noted, this was a matter of interpretation.” I agree that the two interpretations are not the focus of the study. I disagree strongly with the statement that they do not affect the conclusion of this study. Very likely, the route to complexification in the experiment is not what the Authors have modeled. The framework adopted does affect the probability that the H→HP→HPH has actually taken place. Take the last sentence of the Introduction: “We confirmed that the most plausible pathway (the addition of a parasite, followed by the addition of a parasite-resistant host) occurred in our previous evolutionary experiment.” I am sorry, this has very likely not happened.

**Response 2-3:**

We value the opportunity to understand this new viewpoint from the reviewer. Thanks to the detailed explanation of the quasispecies viewpoint by the reviewer, we now understood this better than we previously did and have rewritten the corresponding part of the Discussion as follows.

“In this study, we proposed one of the possible processes of complexification in a host-parasite replicator system. In the process, replication networks evolve by successively acquiring new RNAs from a single RNA to an HP network, followed by an HHP network. However, an alternative view for the complexification process is also possible, in which quasispecies play an important role. A quasispecies comprises a steady-state population of mutationally inter-connected genotypes. The mutation rate associated with RNA replication by Qβ replicates is high (approximately 9.1 × 10^−6^ per nucleotide) (36), corresponding to approximately 0.02 mutations during every replication of host RNA. The RNA population was associated with a large quasispecies from the early stage of the evolutionary experiment, and the members of the quasispecies might have formed a replication network, such as an HHP network, from the beginning. In this alternative view, various replication networks, such as an HHP network, existed in the early stage, and some stable networks dominated the population in the later rounds. To understand the validity and contributions of these different complexification processes, further biochemical analyses of many RNAs included in the quasispecies would be needed.”

We also rewrote the last sentence of the Introduction section as follows (“confirmed” was changed to “proposed”).

“We proposed that the most plausible pathway (the addition of a parasite, followed by the addition of a parasite-resistant host) occurred in our previous evolutionary experiment.”

We are still not sure that we fully understood the quasispecies viewpoint of the reviewer. However, as the reviewer agreed, this issue is not the main focus of this study. Therefore, we would like to stop discussing this issue at this point and leave the interpretation of the experimental results to the readers. Fortunately, all reviewer comments will be presented on the website along with the manuscript in *Plos Computational Biology*, and thus, readers can access the reviewer’s interpretation.

As a final comment, honestly, we think that the review process was a valuable opportunity for us to learn about a new concept related to the complexification process. If possible, we would like to directly discuss this issue with the reviewer in the future.

**Comment 2-4:**

Minor concerns:

While the manuscript has improved notably, there are some parts which I still find obscure. I think that the paper would turn out better if they were addressed, if the Authors wished to do so:

• In the abstract, the Authors wrote: “These results provide experimental evidence that a single replicator spontaneously develops into multi-replicator networks through coevolution with parasitic replicators, which might be the first complexification step toward the emergence of life.”

◦ In my first report, I took issue with this sentence. The Authors replied: “As per our understanding, the reviewer’s concern is the origin of a new RNA species that forms replicator networks.”, then proceeded to reply to anything but my main concern.

◦ No, that was not my issue. My trouble with this sentence is that it suggests that the paper

will deal with the emergence of life (as clearly stated in that sentence), and that is not a topic discussed in this paper at all. I feel that this intends to mislead the reader, honestly.

**Response 2-4:**

Thank you for your additional kind advice. Of course, we wish to improve our manuscript to the best of our abilities. We had misunderstood the reviewer’s comment. As the reviewer indicated, we did not discuss the emergence of life. Accordingly, we removed the corresponding part as follows.

“These results provide experimental evidence that a single replicator spontaneously develops into multi-replicator networks through coevolution with parasitic replicators.~~, which might be the first complexification step toward the emergence of life.~~”

**Comment 2-5:**

• The first paragraph of the first subsection within Methods might be improved:

◦ The first sentence does not make much sense.

◦ In line 547, these are “differential” equations.

**Response 2-5:**

In accordance with the reviewer’s suggestion, the first sentence was revised as follows, and “different” was corrected to “differential” in the revised manuscript. Further, the word “selection” was changed to “culling” according to Reviewer 3’s suggestion.

“In this model, the compartmentalized replication cycle consists of repeating three steps: replication, culling, and fusion-division (Fig. 1).”

**Comment 2-6:**

• I think that the Authors have improved much on the description of the technical aspects. But some issues remain:

◦ What are the units of H_i and P_h? The Authors say that they are concentrations: Why not operate with numbers straight away? Logistic equations are perfectly valid if we let these numbers vary over a continuum.

◦ This is important because: The Authors say that H_i and P_h are concentrations, and that, upon fusion, they are added. What does it mean precisely to add up concentrations? Concentrations are intensive quantities. Merging together two substances of a same size that contain a concentration of 3 parts per million of RNA each does not result in a concentration of 6 parts per million.

◦ My intuition is that these equations represent numbers of copies of each host or parasite, not concentrations. In that case, the procedure discussed by the Authors makes sense. The need to round off these numbers would disappear if, instead of flipping a coin for each molecule, they would draw random numbers from binomial distributions. This would not only be faster, it is also not an approximation, but exact within the stochasticity of the process.

**Response 2-6:**

In the previous manuscript, we wrote that H_i_ and P_h_ are concentrations. The unit was “/ unit volume”. However, the volume of all compartments is one, and thus, the concentration used in this study is virtually the same as the number of replicators in a compartment. When merging two compartments, we treated the numbers in each compartment (not just merging the concentrations). We admit that we essentially handled H_i_ and P_h_ as numbers per compartment in agreement with the reviewer’s advice, and we think that the use of “numbers” instead of “concentrations” is more straightforward. Accordingly, we changed “concentration” to “number per compartment” in the revised manuscript.

In the previous manuscript, we wrote “we flipped a coin for each replicator” to emphasize stochasticity, but in the actual calculation process, we used binomial distributions. To avoid confusion, we rewrote the Methods part to be more precise as shown below (changes are underlined).

“First, two compartments were randomly chosen from all compartments. Second, the numbers of each replicator (i.e., hosts or parasites) in the two compartments were summed to mimic the compartment fusion. Third, the replicators in the fused compartment were randomly redistributed into two new compartments to mimic the division of the compartment according to the binomial distribution. In this step, stochasticity appeared in RNA composition, especially when the number of RNAs was small.”

**Comment 2-7:**

• In my first report, I introduced a toy model to suggest that the stochasticity during the process to select compartments for merging and selection could be affecting the stability of

the model.

◦ I am now partly convinced that this is not the case, given that the Authors have provided many more replicas of each numerical experiment.

◦ However, I find it very odd that the Authors first dismiss this possibility, and then use that precise same argument to explain why so many of their experiments in subsection “Computer simulations of transition of networks” end up extinct. Honestly, I am left with the feeling that the Authors do not have a great grip of their own work.

Response 2-7:

We apologize for this. In the previous reply, we wrote the following sentence to explain the reason for the frequent extinction in the evolutionary simulation.

“*With such a small number of compartments, stochasticity in the selection and RNA distribution would accidentally lead to extinction.*”

We believe that this statement might be wrong because if this was true, we would see the same stochastic extinction in the sustainability analysis conducted in Figs. 3–6 as the reviewer commented. We now think that the reason that led to extinction at a higher rate in the evolutionary simulation would be stochasticity in replication coefficients. In the evolutionary simulation, a newly appearing replicator has randomly chosen parameters. As we showed in the sustainability analysis in Figs. 3–6, replicators become sustainable only in a limited parameter space. Therefore, most of the newly appeared replicators would not satisfy the sustainable conditions and thus disappear quickly. Such stochasticity in replication coefficients might be the major reason for the frequent extinction in the evolutionary simulation.

**Comment 2-8:**

• In Fig. 7:

◦ Could you please clarify what the gray trajectories are? Are we actually observing a transition HPP→HPH, instead of HP→HPH?

**Response 2-8:**

The upper gray line (now pink line) in previous Fig. 7 (shown below) is a parasite, and the lower gray line (still gray) is a host. In previous Fig. 7, we determined the name of the network (HP or HHP) based on the RNAs that survived in the next 100 rounds, and thus, these gray lines were not included in the networks (i.e., the gray line did not last for the next 100 rounds).

**Previous Figure 7. Computer simulation of the evolutionary transition of replication networks.**

The evolutionary transition was simulated by introducing a mutagenesis step in the serial replication cycle, as shown in Fig. 1, for 1000 rounds. (A) The number of networks maintained for more than 100 rounds during replication cycles in 1000 simulations was counted. (B) A typical trajectory of the concentrations in one of the simulations resulted in the formation of the HHP network.

However, now we think that this definition is counter-intuitive and also inappropriate. In the 2^nd^ revised manuscript, we determined the networks based on the RNAs with more than 1,000 because if the number of an RNA is less than 1,000, the RNA should not be contained in most of the compartments (total compartment number is 3,000) and thus should not make a major contribution to the RNA sustainability. According to this new definition, the trajectory became HP→HPP→HP→HHP (new Fig. 7C, shown below). The data in Figs. 7A and 7B did not change with this new definition.

**New Figure 7. Computer simulation of the evolutionary transition of replication networks.**

The evolutionary transition was simulated by introducing a mutagenesis step in the serial replication cycle, as shown in Fig. 1, for 1000 rounds. (A) The number of networks maintained for more than 100 rounds during replication cycles in 1000 simulations was counted. (B) Number of networks that preceded the 218 HHP networks shown in A. (C) A typical trajectory of the total number of each replicator in one of the simulations that resulted in the formation of the HHP network. The network composition was determined based on the replicators with more than 1,000. The replicators with fewer than 1,000 were shown as gray lines. The reason why some replicators started from >100 was that they replicated from 1 to >100 in the round that it appeared.

**Reviewer #3:**

**Comment:**

The manuscript by Kamiura and colleagues connects the experimental results from the Ichihashi group to the mathematical/computational theory of the RNA world.

To do this, they construct their own mathematical model that mimics the well-known experimental system developed in the Ichihashi group. The model consists of a population of protocell-like compartments inhabited by replicating RNAs and possibly parasites. Replicators and parasites grow within compartments, and the compartments undergo fusion, fission, and sampling to introduce new resources. The model, partially parametrised with their experimental data, is used to study the population dynamical stability of different combinations of interacting replicators and parasite species. The authors show that some networks of replicators and parasites can stably coexist. They also identify a plausible route for the evolutionary complexification of the system: parasites evolve to exploit replicators, and new replicator lineages evolve that escape the parasites. Interestingly, this complexification through niche construction turns out to be the same as in an independently derived model by Takeuchi & Hogeweg (2008). The authors further study the long-term stability of their results with evolutionary simulations. Finally, they check the experimental validity of their conclusions by re-analising previous data and running a short experiment with a three species system.

I agree with one of the reviewers that the earlier theoretical results from Takeuchi & Hogeweg (hereafter TH) had to be made more central in the manuscript, as they turned out to be supported by the authors’ model and confirmed by their experiments. To their merit, the authors clearly acknowledge this in the revised manuscript.

It happens rarely in Origin of Life research that an independently derived model is so strikingly confirmed by experiments – I think this is worthy of publication. Moreover, the authors study a model which is slightly different from TH. While the model is a lot simpler than TH, it is close to the authors’ experiments, which provides the “bridge” for the comparison between the authors’ experimental work and the theory developed by TH.

This being said, I had some trouble following the manuscript. I see that the paper has already undergone revision, but I have some general points that I feel should be addressed, plus there are a few parts that really confused me.

**Response:**

We are grateful for the reviewer’s time and effort in evaluating our manuscript, which has led to significant improvements in our manuscript. We are also thankful for the positive comment. The concerns raised by the reviewer have been addressed as described in the following responses.

**Comment 3-1:**

General comments:

1) About the paragraph: “Strategy of theoretical model and analysis”. I found the first paragraph impossible to understand without first having read how the model is constructed. Please explain the model first, and then what you plan to do with it. I think it is fine that the authors put the model in relation to the literature on modelling the RNA world, but as it stands now the model is not explained enough to allow understanding the results obtained with it. So I recommend expanding the model explanation section – perhaps going along Fig. 2 (which could be Fig. 1).

Also, am I correct that the RNAs are assumed to be very stable – so there is no decay in the model, and compartments that reach carrying capacity just halt all replication occurring within them (and only compartments merged with empty ones carry on replication)? If so, this should be explained somewhere, as it is the reason why parasites do not take over every compartment in which they are initially present. I wonder if this is also the case in the experimental system.

**Response 3-1:**

In accordance with the reviewer’s suggestion, we swapped Fig. 1 and Fig. 2 and explained the model first in the revised manuscript.

Regarding RNA decay, our model does not include the degradation process of RNA because RNA degradation is not detectable in our experimental system. This point has been added to the Methods section as follows.

“This model does not include RNA degradation because it is negligible in the experimental system (30).”

**Comment 3-2:**

2) Do I understand correctly that 100 instances of the HH network are run each for 100 rounds? If so, I find this problematic. As far as I can tell (though I might be wrong) the only difference between two such instances is some randomness in the composition of the compartments. So if e.g. 20 runs out of 100 survive after 100 time steps – how many survive after 200 time steps? After 1000? The point here is: is long-term coexistence stable whenever a small number of runs survive to 100 time steps? I suspect not, but either way this should be explicitly stated/explained. Also, the plots do not clearly discriminate the runs that go extinct (if any) and those in which the original complexity is lost and a simpler network of species remains.

**Response 3-2:**

We ran the simulation of the sustainability analysis (Figs. 3-6) for 100 rounds, 100 times, and determined whether all replicators existed at the final round. As the reviewer pointed out, the result does not guarantee longer sustainability. This point has now been added in the revised manuscript as follows.

“It should be noted that the sustainability evaluated here was limited to up to 100 rounds, and longer sustainability was not guaranteed. This is also the case for the following simulations.

>Also, the plots do not clearly discriminate the runs that go extinct (if any) and those in which the original complexity is lost and a simpler network of species remains.

We did not include these data in the previous manuscript because the remaining simpler networks were not the focus of this study. However, admittedly, these data can be informative for readers. In accordance with the reviewer’s suggestion, we have now included the number of runs in which each of the simpler networks survived for all networks analyzed in this study, as new Figs. S5–S13 (not shown below because they are too large). To describe these data, we included new sentences in the Results section as follows.

“In all aforementioned analyses, we counted only the numbers of sustainable runs in which all initial members of the network existed after 100 rounds. We also counted the number of runs in which some of the replicators existed after 100 rounds. The data are shown in Fig. S5–S13 for all networks. “

**Comment 3-3:**

3) I think the authors sufficiently frame their results in the context of the theory from TH. However, the differences between models (and between TH model and the authors’ experiments) should be also emphasised. A detailed comparison would highlight what is general and what is specific to the models, and would make for a more insightful discussion.

**Response 3-3:**

In accordance with the reviewer’s suggestion, we added explanations of the difference between our theoretical or experiment models and Takeuchi and Hogeweg’s model in a new paragraph in the Discussion section, as shown below.

“This study focused on the issue of how replicator networks evolve in terms of their complexity, which was similar to the previous theoretical study by Takeuchi and Hogeweg (16). Details of the theoretical and experimental models used here are different from that presented previously. For replication, the model by Takeuchi and Hogeweg (TH model) assumes complex formation among RNA replicators based on their secondary structures and base pair matching, whereas in our experimental system, the RNA replication is catalyzed by a protein enzyme translated from an RNA replicator, and the interaction between the enzyme and RNA replicator is primarily determined by the template specificity of the encoded replication enzyme. Although working molecules are different, a common point for both TH and our experimental models is that the RNA sequence (via translation in our experiment) plays a central role in determining which RNA is replicated. Our theoretical model does not assume any underlying mechanism for the template specificity but only assigns replication coefficients. For spatial structure, the TH model assumes a two-dimensional square grid, and one grid contains at most one RNA molecule, which can interact with RNA molecules in the adjacent grids. In our theoretical and experimental models, we used compartment structures, which can contain many RNA molecules. All RNAs in the same compartment can interact with each other. For the propagation of RNAs, the TH model assumes diffusion of the replicated RNAs into adjacent grids. In our theoretical and experimental models, RNAs disperse into different compartments via occasional random fusion and division among any of the compartments in the system. Considering that a similar complexification process occurred in both the TH and our models, these differences in the replication, spatial structure, and diffusion process are not critical factors for the evolution of complex replicator networks.”

**Comment 3-4:**

Detailed comments:

Line 47: you mean “evolves” here, not “develops”, right?

**Response 3-4:**

In accordance with the reviewer’s suggestion, we rewrote the sentence in the revised manuscript.

**Comment 3-5:**

Line 93: The sentence mentions hypercycles, but reference 22 is about proto-chromosomes (linked replicators). Is this reference in the right place?

**Response 3-5:**

Thank you for pointing this out. This was a mistake, and we have removed the reference from the revised manuscript.

**Comment 3-6:**

95: “… studies have revealed that spatial structures such as compartmentalization repress …”

The sentence sounds like compartmentalisation is a subset of spatial structure, but it should be the other way around.

**Response 3-6:**

We believe that some researchers in this field use “compartmentalization” as a subset of the spatial structure, but we agree that some do not. Here, to avoid confusion, we rewrote the corresponding sentence in the revised manuscript as follows (altered parts are underlined).

“To date, theoretical (23–27) and experimental (28,29) studies have revealed that spatially restricted systems, such as compartmentalization, repress parasitic replicators.

**Comment 3-7:**

101: please change “developed” to “evolved” in the sentence about ecological complexity.

Also, the term “by successive addition” is incorrect. In Takeuchi & Hogeweg’s study, the parasite evolves spontaneously – exactly like in the author’s experimental system.

**Response 3-7:**

In accordance with the reviewer’s suggestion, we rewrote the sentence in the revised manuscript. The “by successive addition” was changed to “by successive appearance.”

**Comment 3-8:**

99 and 104: the term “compartmentalised structure” is used. Takeuchi & Hogeweg’s model is spatially structured. RNAs are implicitly compartmentalised because of local interactions – but I do not understand what the term means.

**Response 3-8:**

We used the phrase “compartmentalized structures” to mean a structure that restricts diffusion, but we agree that this was misleading. In the revised manuscript, we used the more precise phrase “two-dimensional square grid”.

**Comment 3-9:**

105 – 110: These two sentences are somewhat repeated from line 100 – 105. Something went wrong with the formatting?

**Response 3-9:**

Thank you for pointing this out. This was a mistake, and we have removed the duplicated content from the revised manuscript.

**Comment 3-10:**

131: the word “appearance” here should really be “evolution”.

**Response 3-10:**

In accordance with the reviewer’s suggestion, we corrected this sentence.

**Comment 3-11:**

135: I am not convinced by the word “generalised”. What makes your model more general? Wouldn’t you say that your model is analogous to Takeuchi & Hogeweg’s, but follows your experiment more closely?

**Response 3-11:**

We used that word to mean that our model is applicable to our experimental system. We admit that the word was inappropriate and have rewritten the sentence as follows (changes are underlined).

“To this end, we first constructed a theoretical model of a compartmentalized host-parasite replication system, which is conceptually the same as that developed by Takeuchi and Hogeweg but formatted to be similar to our experimental system.”

**Comment 3-12:**

155: I don’t understand the following: “... the parameter spaces that allow for the sustainable replication of all members in the networks for certain generations.”. Do you mean that you run the system’s dynamics for a fixed number of time steps?

**Response 3-12:**

Yes, the reviewer’s understanding is correct. To clarify the meaning, we rewrote this sentence as follows.

“In this study, we evaluated the stability of each network by running a simulation with various parameters for certain time steps (rounds) and measuring how many times all members in the network existed in the last time step.”

**Comment 3-13:**

163: In the sentence “The replication is continued by repeating three steps: replication, selection, and fusion-division”, the word “replication” appears twice, but it has two different meanings. Please use a different word (perhaps the first “replication” could be substituted with “Within-compartment dynamics”). Notice that this sentence re-appears in Material and Methods, where it should also be changed. Moreover, I find the word “selection” here a bit puzzling: aren’t you removing 25% of the compartments without specifically selecting for any property? Perhaps “culling” could be a better word.

**Response 3-13:**

We rewrote the sentences in both the Results and Methods sections as follows. We also changed the word “selection” to “culling” according to the reviewer’s suggestion.

“In this model, the compartmentalized replication cycle consists of repeating three steps: replication, culling, and fusion-division (Fig. 1).”

**Comment 3-14:**

197: a parenthesis is opened but not closed.

**Response 3-14:**

We have corrected this in the revised manuscript

**Comment 3-15:**

243: Sentence: “These results suggest that even if an HPP network is formed during evolution, it will soon return to the HP network.” This is interesting, but no data is shown to support this.

**Response 3-15:**

As shown in Response 3-2, we have added the number of HP networks after 100 rounds in Fig. S7C.

**Comment 3-16:**

343-352 and Fig. 7: it is difficult to extract information from the figure. Fig. 7A is partially a bar chart (frequency of evolved networks) and partially numbers (presumably the frequency of ancestral networks of HHP). Please make the information homogeneous in the figure. In Fig. 7B, how can I infer that HHP descends from HP? Moreover, are the lines representing concentrations over one compartment or multiple ones? There is no caption/legend for the grey lines. Why are the initial concentrations of Host 2 and Parasite = 10^2?

**Response 3-16:**

In accordance with the reviewer’s suggestion, the numbers in previous Fig. 7A were changed to a bar chart in the revised manuscript, as shown below (new Figs. 7A and 7B).

Regarding previous Fig. 7B (new Fig. 7C), based on the previous definition, we determined the existing network from the replicators that survived in the next 100 rounds, and thus, we did not include these previous gray lines in the networks (because the gray line did not last for next 100 rounds). However, we now think that this definition is counter-intuitive and also inappropriate. In the revised manuscript, we determined the composition of the network (e.g., HP, HHP, and so on) based on the replicators with more than 1,000 because if replicators with less than 1,000, the replicator should not be contained in most of the compartments (the compartment number was 3,000); thus, they would not make a major contribution to the sustainability of replicator network. According to this new definition, the trajectory in new Fig. 7C became HP→HPP→HP→HHP (shown below). The data in new Figs. 7A and 7B did not change with this new definition.

The previous Y-axis label “Concentration” was incorrect. The Y-axis represents the total number of replicators in all compartments. This point was corrected in the revised manuscript. The reason why the previous Host 2 (now Host 3) and previous Parasite (now Parasite 2) started from 10^2^ was that Host 2 replicated from 1 to 10^2^ in the first round soon after its appearance. This information has been added to the legend in the revised manuscript.

**New Figure 7. Computer simulation of the evolutionary transition of replication networks.**

The evolutionary transition was simulated by introducing a mutagenesis step in the serial replication cycle, as shown in Fig. 1, for 1000 rounds. (A) The number of networks maintained for more than 100 rounds during replication cycles in 1000 simulations was counted. (B) Number of networks that preceded the 218 HHP networks shown in A. (C) A typical trajectory of the total number of each replicator in one of the simulations that resulted in the formation of the HHP network. The network composition was determined based on the replicators with more than 1,000. The replicators with fewer than 1,000 were shown as gray lines. The reason why some replicators started from >100 was that they replicated from 1 to >100 in the round that it appeared.

**Comment 3-17:**

358: about the sentence: “We found that 170 out of 218 HHP networks satisfied this condition”. It would be interesting to see the evolutionary trajectory (or at least the evolutionary steady states) of the various evolutionary parameters.

**Response 3-17:**

In accordance with the reviewer’s suggestion, we presented the parameters of 218 HHP networks that appeared in the evolutionary simulation as supplemental data, as a list. We could not visualize the data as a figure because the parameter values (four $k_{ji}^{H}$ and two $k_{h1}^{P}$) are highly interdependent and no clear trend other than that described in the main text was found. We did not include the evolutionary trajectory of the parameters in the data because the data contain parameters of many networks that appeared only for a short time, which is probably not informative to readers.

**Comment 3-18:**

414: I do not understand the sentence “These small branches are expected to be derived from quasispecies”.

**Response 3-18:**

In the revised manuscript, we rewrote the entire paragraph to respond to another comment by Reviewer 2, and this sentence has now been removed.

**Comment 3-19:**

415: You state: “The interpretation of this result based on quasispecies is discussed later.”. But later, in the discussion, the results are not really discussed – you simply state that you do not expect Host 1 and 2 to be part of the same quasispecies (more on that below).

**Response 3-19:**

We agreed with the reviewer’s comment. We removed this sentence in the revised manuscript.

**Comment 3-20:**

474: I do not understand the definition of quasispecies is as “a population of variants with random mutations”. A quasispecies is a (steady-state) population of mutationally inter-connected genotypes.

**Response 3-20:**

We agree with the reviewer’s comment and corrected the definition to “a steady-state population of mutationally inter-connected genotypes.” We also rewrote the entire paragraph according to the comments of Reviewer 2 as follows.

“In this study, we proposed one of the possible processes of complexification in a host-parasite replicator system. In the process, replication networks evolve by successively acquiring new RNAs from a single RNA to an HP network, followed by an HHP network. However, an alternative view for the complexification process is also possible, in which quasispecies play an important role. A quasispecies comprises a steady-state population of mutationally inter-connected genotypes. The mutation rate associated with RNA replication by Qβ replicates is high (approximately 9.1 × 10^−6^ per nucleotide) (36), corresponding to approximately 0.02 mutations during every replication of host RNA. The RNA population was associated with a large quasispecies from the early stage of the evolutionary experiment, and the members of the quasispecies might have formed a replication network, such as an HHP network, from the beginning. In this alternative view, various replication networks, such as an HHP network, existed in the early stage, and some stable networks dominated the population in the later rounds. To understand the validity and contributions of these different complexification processes, further biochemical analyses of many RNAs included in the quasispecies would be needed.”

**Comment 3-21:**

480: I do not understand what this sentence means: “The existence of many different but closely related genotypes can be explained by quasispecies”. Do you mean that you expect these closely related genotypes to be part of the same quasispecies?

**Response 3-21:**

We agree with the reviewer’s comment. That is our intended meaning. However, as we wrote in the above response, we have rewritten the corresponding paragraph and removed this sentence in the revised manuscript.

**Comment 3-22:**

481-484: You report Hamming distance = 7 between Host1 and Host2 as a way to argue that they belong to different quasispecies. This is a result and should be moved to the Results section. But aside from this, I do not understand how two sequences separated by 7 nucleotides out of 2041 (the length of Host 1) can be different quasispecies. Can you clarify why Host 1 and Host 2 are not variants within the same quasispecies (with Host 2 better able to escape the parasite)?

**Response 3-22:**

We feel that this point was troublesome and not the focus of this study. Therefore, we removed these sentences from the revised manuscript.

**Comment 3-23:**

533: I do not understand what message this sentence is conveying: “It is of utmost importance how many RNAs participate in a network, what determines the maximum number, and whether the different RNAs fuse to become a single molecule that encodes more information, which may lead to the origin of chromosome (22).”. In particular, I do not understand the connection between RNA networks and evolution of chromosomes.

**Response 3-23:**

To clarify the meaning, we rewrote these sentences as follows (changes are underlined).

“Indeed, we recently reported that after 240 rounds of serial replication cycles, a five-member network that consists of three hosts and two parasites appeared, in which the host RNAs have asymmetric resistance to the two parasites (31). The following points are of utmost importance: how many types of RNAs participate in the next network, what determines the maximum number of members in a network, and whether the RNA members in a network eventually fuse to become a single molecule that encodes more information, which might lead to the origin of a multicistronic RNA genome (48).”
