# Supplementary material for: Plausible pathway for a host-parasite molecular replication network to increase its complexity through Darwinian evolution
Source: PLoS Comput Biol. 2022 Dec 1;18(12):e1010709. doi: 10.1371/journal.pcbi.1010709 (PMC9714742; doi:10.1371/journal.pcbi.1010709)
Supplement: Attachment — Submitted filename: 221101_point-by-point_response.docx [file pcbi.1010709.s023.docx]

Point-by-point responses

**Reviewer #1:**

**Comment:**

I would like to comment on the issue of quasispecies, which was discussed by the other reviewers and authors. If I understand it correctly, the critical question appears to be whether Hosts 1 and 2 belong to the same quasispecies or not. If they belong to the same quasispecies, it appears illegitimate to model the populations of Hosts 1 and 2 as those of two distinct species, potentially undermining the authors' interpretation of their experimental results as embodied in their model, as suggested by another reviewer.

To address the above question, we need an empirical test to judge whether or not two genotypes belong to the same quasispecies. One possible test might be to check whether two genotypes are separated by a sufficiently large Hamming distance, but such a test is problematic because it is unclear how large the distance must be, as discussed by the other reviewers. A better test might be to examine whether two genotypes can coexist with each other without any mutation. If they can, the result is compatible with the interpretation that the two genotypes belong to separate quasispecies. The test is not definite proof, but it is a good start because it is simple. It is also in line with the original motivation behind the concept of quasispecies. In their seminal work on the hypercycle, Eigen and Schuster asked how macromolecules accumulate information through Darwinian evolution. They discovered that the amount of information one quasispecies can accumulate is bounded above (aka information threshold). So, they looked for a way to evolve multiple quasispecies in one system because that would increase the system's information capacity. Thus, they proposed a hypercycle, in which different genotypes coexist with each other, not by mutating into each other as in a quasispecies, but by catalysing each other's replication in a cyclic manner. Therefore, coexistence without mutation would support the existence of multiple quasispecies.

A test similar to the above has been, in fact, already performed by the authors, as depicted in Figure 9C. This figure shows that three genotypes sampled from Hosts 1 and 2 and Parasite can coexist without the initial presence of the other genotypes. The chance of mutations creating Host 1 from Host 2 (or vice versa) in 7 replication steps is about [0.02 * (1/2000)]^7 ~ 1e-35 (0.02 is the mutation rate per genome, and 2000 is the genome size). So, the mutational influx between Hosts 1 and 2 is negligible, at least for the first ten or so rounds of replication. Moreover, the authors might have data to check if any genotypes other than the initial three become prominent within the first 20 rounds of replication. If the initial three genotypes remain to be the majority during this time, it indicates that the mutational influx is negligible for the first cycle of oscillation. That would support the interpretation that Hosts 1 and 2 can survive without mutating into each other. A result like that might be the best evidence one could hope for the quasispecies issue until a better test is proposed in the future.

This is a follow-up to the above discussion. In response to a comment by another reviewer, the authors removed the paragraph discussing whether Hosts 1 and 2 belong to the same quasispecies based on the Hamming distance. Although I agree that the paragraph is not quite tenable, I also find it useful if the paper contains information about the Hamming distance between three representative genotypes of Hosts 1 and 2, and Parasite as well as their genome lengths in one location (I was not able to find this information in the other parts of the manuscript). So, I suggest that the authors put this information back. In addition, I suggest that the authors explain the meaning of the scale of branch lengths in the caption of Figure 8, as I was not able to find this information in the manuscript.

Overall, I think this manuscript makes an important contribution to the field. I agree with another reviewer saying that it is rare in this field to see that a theoretical prediction receives experimental support. I am of the opinion that this manuscript, combining both theoretical and empirical approaches, contains enough body of evidence to warrant publication. So, I recommend the publication of this manuscript in this journal.

**Response:**

We are thankful to the reviewer for the kind advice. We agree that the coexistence of Host 1, Host 2, and Parasite in Fig. 9C can be supporting evidence that Host 1 and Host 2 belong to separated quasispecies. We think that we should have mentioned this experiment in the previous response to another reviewer. But, we understand that even with this evidence, we cannot deny the quasispecies view and thus, would like to find a better experiment to examine it. Probably, the direct visualization of quasispecies with deep and precise sequencing would be required.

In accordance with the reviewer’s suggestion, the information on the Hamming distances between Host 1 and 2, and Parasite were included in the Result section of the revised manuscript as follows. The scale of branch length in the previous manuscript was the Hamming distance normalized by the number of mutated sites, which we think is a little difficult to interpret. In the revised manuscript, we changed the scale to Hamming distance. The explanation is included the explanation in the legend.

“Host1exp and Host2exp are separated by a Hamming distance of 7. Parasite1exp are separated from Host1exp and Host2exp by Hamming distances of 8 and 6, respectively, except for the large deletion.”

**Reviewer #3:**

**Comment**

I have a couple of very minor comments. After these comments are addressed, I do not need to review the paper again, and I am happy to recommend that the article is accepted.

Fig 1b: the second sum sign lacks subscripts.

Line 166, “in which” should be “with which”.

Line 176, “1,7” should be “1.7”, right?

Line 277, 280, 284, the word “point” is used? Can you please change this to “number of surviving simulations” or something similar?

Line 417, I do not understand what “branches derived from quasispecies” means. Do you mean (as I already asked in the previous round) that you expect that these genotypes belong to the same quasispecies? If not, could you describe what of quasispecies theory you are referring to?

To me, these short branches seem just a consequence of the evolutionary process: some mutants survive long enough for you to be able to sequence them – but I cannot see where the quasispecies part is.

**Response:**

We are thankful for the reviewer’s comment, which indicates our mistakes and unclear sentences. We corrected all the points in the revised manuscript.
